# Craniodental and humeral morphology of a new species of *Masrasector* (Teratodontinae, Hyaenodonta, Placentalia) from the late Eocene of Egypt and locomotor diversity in hyaenodonts

**DOI:** 10.1371/journal.pone.0173527

**Published:** 2017-04-19

**Authors:** Matthew R. Borths, Erik R. Seiffert

**Affiliations:** 1 Department of Biomedical Sciences, Heritage College of Osteopathic Medicine, Ohio University, Athens, Ohio, United States of America; 2 Department of Integrative Anatomical Sciences, Keck School of Medicine, University of Southern California, Los Angeles, California, United States of America; Institute of Vertebrate Paleontology and Paleoanthropology Chinese Academy of Sciences, CHINA

## Abstract

Hyaenodonta is a diverse clade of carnivorous mammals that were part of terrestrial faunas in the Paleogene of Eurasia and North America, but the oldest record for the group is Afro-Arabian, making the record there vital for understanding the evolution of this wide-spread group. Previous studies show an ancient split between two major clades of hyaenodonts that converged in hypercarnivory: Hyainailourinae and Hyaenodontinae. These clades are each supported by cranial characters. Phylogenetic analyses of hyaenodonts also support the monophyly of Teratodontinae, an Afro-Arabian clade of mesocarnivorous to hypercarnivorous hyaenodonts. Unfortunately, the cranial anatomy of teratodontines is poorly known, and aligning the clade with other lineages has been difficult. Here, a new species of the phylogenetically controversial teratodontine *Masrasector* is described from Locality 41 (latest Priabonian, late Eocene) from the Fayum Depression, Egypt. The hypodigm includes the most complete remains of a Paleogene teratodontine, including largely complete crania, multiple dentaries, and isolated humeri. Standard and “tip-dating” Bayesian analyses of a character-taxon matrix that samples cranial, postcranial, and dental characters support a monophyletic *Masrasector* within Teratodontinae, which is consistently placed as a close sister group of Hyainailouridae. The cranial morphology of *Masrasector* provides new support for an expanded Hyainailouroidea (Teratodontinae + Hyainailouridae), particularly characters of the nuchal crest, palate, and basicranium. A discriminant function analysis was performed using measurements of the distal humerus from a diverse sample of extant carnivorans to infer the locomotor habits of *Masrasector*. *Masrasector* was assigned to the “terrestrial” locomotor category, a result consistent with the well-defined medial trochlear ridges, and moderately developed supinator crests of the specimens. *Masrasector* appears to have been a fast-moving terrestrial form with a diverse diet. These specimens considerably improve our understanding of Teratodontinae, an ancient member of the Afro-Arabian mammalian fauna, and our understanding of hyaenodont diversity before the dispersal of Carnivora to the continent near the end of the Paleogene.

## Introduction

The modern African terrestrial carnivore fauna is primarily composed of species from Carnivora, but members of that order only appear in the Afro-Arabian fossil record during the latest Oligocene [1–2]. For most of the Paleogene in Afro-Arabia, terrestrial carnivorous niches were occupied by Hyaenodonta, an extinct radiation of placental mammals whose members have also been found in Europe, Asia, and North America. Hyaenodonts were morphologically diverse, ranging from the small, weasel-sized *Proviverra typica* [3] to the wolf-sized *Hyaenodon horridus* [4], and even up to the rhinoceros-sized *Megistotherium osteothlastes* [5]. Coupled with their extensive range in body size is a diversity of cranial, postcranial, and dental adaptations that allowed hyaenodonts to exploit arboreal, mesocarnivorous niches to cursorial, hypercarnivorous niches [6–8].

Unfortunately, the Paleogene Afro-Arabian radiation of hyaenodonts is still not well understood. One reason for this may be that the fossil record of this group is dominated by dental specimens; only five taxa (“*Pterodon*” *africanus*, *Apterodon macrognathus*, *Megistotherium osteothlastes*, and the recently published [9] *Brychotherium ephalmos*, and *Akhnatenavus nefertiticyon*) are known from substantial cranial material, and only a few postcranial elements have been described [5, 10–11]. As such, we know little about the lifestyles of early Afro-Arabian hyaenodonts, aside from the inference that they all were, to some extent, carnivorous based on dental comparisons with modern Carnivora [12]. The Afro-Arabian record stands in contrast to the more complete record of hyaenodont remains from Europe, North America, and Asia [3, 11–18]. The record from North America, in particular, has provided our baseline understanding of early hyaenodont cranial and postcranial morphology [4, 6, 13, 16, 19, 20].

Teratodontinae, originally erected to contain the dentally bizarre early Miocene genus *Teratodon* [21], which possesses massive, bunodont premolars, was first recognized as a clade by Solé et al. [22]. Teratodontinae is a clade of largely Afro-Arabian species, the early members of which appear to have been dietary generalists [9, 22]. In contrast, known Miocene species show great disparity in dental morphology and body size [8, 21, 27]. Borths et al. [9] found that the following teratodontines consistently form a clade to the exclusion of all other hyaenodonts in a majority of the trees generated using parsimony and Bayesian methods—early-middle Eocene *Furodon* and *Glibzegdouia* from Algeria [22]; late Eocene *Brychotherium* from Egypt [9]; early Oligocene *Masrasector* from northern Afro-Arabia [23–25]; early Miocene *Teratodon* from Egypt, Kenya, and Uganda [21, 26]; early Miocene *Anasinopa* from Kenya [21]; and middle-late Miocene *Dissopsalis* from Kenya and south Asia [27]. More broadly, these teratodontines were placed as a sister group of another largely Afro-Arabian clade, Hyainailouridae (Hyainailourinae + Apterodontinae) in a majority of parsimony- and Bayesian-derived consensus trees. Borths et al. [9] proposed that the clade that includes Teratodontinae, Hyainailourinae, and Apterodontinae be called Hyainailouroidea, and that name is used here. Hyainailouroidea may or may not include Eocene Asian “indohyaenodontines” (*Indohyaenodon*, *Paratritemnodon*, and *Kyawdawia*) and early Oligocene African *Metasinopa*, as their positions differed in the analyses of Borths et al. [9] depending on the phylogenetic method employed.

Previously, the species that are now recognized as teratodontines were placed in various positions relative to other hyaenodonts, either as (1) part of a generalist group with European and Asian taxa [27], (2) part of an “Afroasian proviverrine” clade whose interrelationships implied multiple dispersal events between Afro-Arabia and Asia [28]; (3) a sister group of European Proviverrinae and *Arfia* [22]; (4) a sister group of Hyainailourinae [29]; and (5) as members of Hyaenodontidae (the group that includes *Hyaenodon*) by Solé et al. [30], based on the cranial reconstruction of *Dissopsalis* by Colbert [31]. Some of the ambiguity seeded by taxa placed in Teratodontinae may result from the limited cranial material known from the group, and the total absence of referred postcrania—both of which are anatomical regions that Polly [6] and Rana et al. [29] demonstrated were rich sources of character information for understanding the relationships among hyaenodonts. Polly [6] and Solé et al. [30] emphasized the morphology of the nuchal crest and neurocranium as particularly important for distinguishing Hyainailouridae from Hyaenodontidae. Borths et al. [9] described rostral material from the teratodontine *Brychotherium* that significantly improved understanding of early teratodontine cranial morphology; however, the occipital region of that genus is not yet known.

Here, we describe multiple specimens that belong to a new species of *Masrasector*. Other species of *Masrasector* are known from younger deposits in the Fayum succession [23], the Dhofar region of Oman [24], and Bir el Ater in Algeria [25]. The fossils described here were recovered from Locality 41 (L-41), a latest Priabonian (latest Eocene, ~34 Ma) locality in the lower sequence of the Jebel Qatrani Formation in the Fayum Depression of Egypt. The hypodigm includes multiple complete—though distorted—crania, importantly with occipital regions intact; there are also multiple mandibular specimens and three isolated distal humeri. This description makes the L-41 species of *Masrasector* one of the most completely known Afro-Arabian hyaenodonts, and provides an opportunity to further test the hypothesis of a close relationship between Teratodontinae and Hyainailouridae [9, 29]. The isolated distal humeral specimens are well preserved and are integrated into a multivariate morphometric analysis that provides the first evaluation of ecomorphological diversity among Afro-Arabian hyaenodonts within the context of a carnivoran comparative sample.

### Institutional abbreviations

**AMNH**, American Museum of Natural History, New York; **BNHM** Natural History Museum, London; **CGM**, Cairo Geological Museum, Cairo; **DPC**, Duke Lemur Center, Division of Fossil Primates, Durham; **KNM**, National Museums of Kenya, Nairobi; **MCZ**, Museum of Comparative Zoology, Harvard University, Cambridge.

## Materials and methods

### Collecting and permits

Permission to collect and export fossils was granted by the Egyptian Mineral Resources Authority (formerly the Egyptian Geological Survey and Mining Authority) and the Egyptian Geological Museum.

### Geological context

The material described here was collected from Locality 41 (L-41) in the Fayum Depression, Egypt over the course of several decades of excavation. The Fayum area preserves a near-continuous terrestrial record from the early late Eocene through the early Oligocene [32]. Quarry L-41 is the oldest productive vertebrate fossil locality in the lower sequence of the Jebel Qatrani Formation, and was deposited during a period of reversed polarity [33] that Seiffert [34] correlated with Chron C13r. Within Chron C13r, Seiffert [34] argued that L-41 was latest Priabonian (latest Eocene, ~34 Ma) in age based on the presence of a major unconformity above the fossil-bearing layer that might have been due to nearshore erosion associated with the major drop in sea level that occurred in the earliest Oligocene. L-41 is a well-consolidated deposit dominated by clays, and the locality preserves complete or near-complete (though often crushed) crania, dentaries, and isolated postcranial fossils [35]. The fine-grained clays of L-41 are particularly important for preserving the smaller components of the mammalian fauna in the Fayum, such as small primates [36–39].

### Morphological measurements and nomenclature

**Fig 1:**
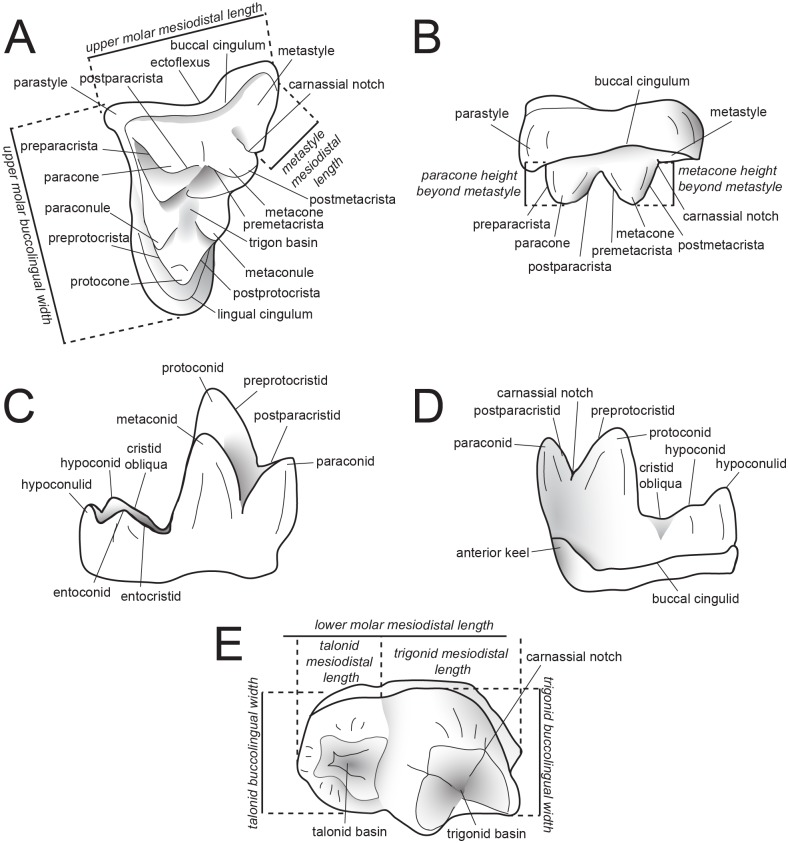
Dental nomenclature used in this study. Upper left M^2^ and lower left M_3_ of *Proviverra typica* (A–E) showing dental terminology and measurements used in this study. (**A**) *Proviverra typica* M^2^ in occlusal and (**B)**, buccal views and M_3_ in (**C)**, occlusal (**D)**, lingual, and (**E)**, buccal views. Measurements are indicated in italics. Modified from [9].

Dental measurements of the specimens were collected from digital photographs using ImageJ [40] following Holroyd [41]. Dental nomenclature also follows Holroyd [41]. Measurements and dental nomenclature are illustrated in Fig 1.

### Nomenclatural acts

The electronic edition of this article conforms to the requirements of the amended International Code of Zoological Nomenclature, and hence the new names contained herein are available under that Code from the electronic edition of this article. This published work and the nomenclatural acts it contains have been registered in ZooBank, the online registration system for the ICZN. The ZooBank LSIDs (Life Science Identifiers) can be resolved and the associated information viewed through any standard web browser by appending the LSID to the prefix “http://zoobank.org/”. The LSID for this publication is: urn:lsid:zoobank.org:pub: ADB4072C-5937-4E33-92F2-DC05EBFA2E65. The electronic edition of this work was published in a journal with an ISSN, and has been archived and is available from the following digital repositories: PubMed Central, LOCKSS. The physical specimens described here with a CGM specimen code are deposited at the Cairo Geological Museum, Cairo, Egypt and specimens described here with a DPC specimen code are deposited at the Duke Lemur Center, Division of Fossil Primates, Duke University, Durham, NC.

### CT scanning and rendering

μ-CT scans were collected at the Duke University Shared Materials Instrumentation Facility using a Nikon XTH 225 ST scanner. Three-dimensional surface models were constructed using ImageJ and Avizo 7.0. Additional surface model manipulation and measurements were conducted in MeshLab v1.3.3 [42]. Digital models of all specimens scanned as part of this study are available on Morphobank (Project P191) <www.morphosource.org>.

### Body mass estimation

Body mass in hyaenodonts is difficult to estimate because there are no living taxa analogous to these large-headed placental carnivores with multiple carnassials [15, 43]. Several studies have used regression equations derived from modern carnivoran body masses and craniodental dimensions to estimate body mass in hyaenodonts (e. g. [17, 28, 29, 44]). In this study we chose to utilize four separate equations to estimate the body mass of the new species of *Masrasector* from L-41. The first equation is based on carnivoran M_1_ mesiodistal length and was utilized by Morlo [15]. Morlo chose to use the average mesiodistal length of the carnassial-bearing lower molars (mm) to calculate body mass. We applied the regression equation of Morlo [15] (body mass = 10 ^ [3.5104 * log10(mesiodistal molar length)−2.6469]) to the length of each individual molar (M_1_, M_2_, and M_3_) and the average length of the molars, yielding four separate body mass estimates. We also followed Egi et al. [28] and utilized body mass estimates described by Van Valkenburgh [44]. Van Valkenburgh [44] calculated separate regression equations for carnivorans of different body mass classes. Based on comparisons between the cranial specimens of the new L-41 taxon and extant carnivorans, we chose to use Van Valkenburgh’s equations for carnivores with a body mass <6 kg. Van Valkenburgh [44] calculated one dental regression equation based on M_1_ mesiodistal length (mm). We applied this regression equation (body mass = 10 ^ [(1.21 * log10(molar length)−0.93]) to each molar length and to average molar length. Van Valkenburgh also calculated a regression equation for body mass based on total skull length (mm) that was estimated from DPC 11990 and DPC 12157 (body mass = 10 ^ [2.55 * log10(skull length)−4.56]) and occiput to orbit length (mm) that was estimated from the occipital condyles to the anterior orbit of the same specimens (body mass = 10 ^ [2.70(log10(occiput to orbit length)−4.55]).

### Morphological character matrix

Two Bayesian analyses were conducted to place the new species of *Masrasector* in a phylogenetic context within Hyaenodonta and to further test hypotheses of hyaenodont interrelationships (e.g. [6, 9, 15, 28, 45]). Of particular interest in this study are the relationships among species included in Teratodontinae by previous analyses (*Dissopsalis*, *Anasinopa*, *Teratodon*, *Masrasector*, *Brychotherium*, and *Glibzegdouia* in [22]; *Furodon* in [9]) and the possibly para- or monophyletic Indohyaenodontinae (*Indohyaenodon*, *Paratritemnodon*, *Kyawdawia*, and possibly African *Metasinopa*), as well as the monophyly of Hyainailouroidea. The character-taxon matrix used in this analysis was modified from [9] and it includes 134 characters and 77 operational taxonomic units (OTUs—3 outgroup taxa and 74 hyaenodonts, all species level OTUs except *Teratodon* and *Lesmesodon*, which are composites of material referred to each of these genera). Inapplicable characters were reductively coded [46]. Eighteen multistate characters were treated as ordered with reference to outgroup morphology following the recommendations of Slowinski [47], and all characters were equally weighted. All specimens were scored in Mesquite [48]. Character descriptions with citations and information on ordering are presented in S1 Table. The nexus file containing the character-taxon matrix is S1 Dataset. Additional information on OTUs including age, formation, and locality are presented in S2 Table.

### Standard Bayesian inference

Bayesian phylogenetic inference analysis was performed using MrBayes 3.2.3 [49]. Morphological data used the M_k_ model [50], data type for the analysis was set to “standard,” and coding set to “variable”. The analysis was run for 10x10^6^ generations. Two runs were performed simultaneously with four Markov Chains. Three were heated (temp = 0.02) and sampled every 5000 generations to avoid autocorrelation. The burn-in period was set as the first 25% of sampled trees, and these were discarded. The resulting posterior probabilities (PP) are listed to the right of the relevant nodes in the Bayesian inference “allcompat” (majority-rule plus all compatible groups) tree. The input file formatted for MrBayes is S2 Dataset.

### Bayesian “Tip-dating”

The analysis was run in MrBayes 3.2.3 [49] following the methods employed by Beck and Lee [51] and building upon the analysis presented by Borths et al. [9]. As in the standard Bayesian analysis, the M_k_ model was employed. The Independent gamma rates (IGR) relaxed clock model [49, 52] was used to infer divergence ages and calculate morphological evolutionary rates [53]. The in-group was constrained to include only Hyaenodonta, excluding *Tinerhodon*, which was recovered outside Hyaenodonta using parsimony analysis and standard Bayesian analysis, both in this analysis and in the previous analysis of Borths et al. [9]. The root of the tree was set with a prior of 120–130 Ma [54, 55] and the prior for the divergence date of Hyaenodonta was set to be between 70 Ma and 62 Ma, a step recommended by Beck and Lee [51] to account for the tendency of tip-dating to recover the ancient divergences observed by Arcila et al. [56]. The analysis was performed over 50x10^6^ generations. The priors that produced the best evidence for convergence across all parameters was clockratepr = normal(0.01, 0.007), and igrvarpr = exp(3). Using these priors, two runs were performed simultaneously with four Markov chains. Three Markov chains were heated (temp = 0.02). A total of 10,000 generations were sampled, and the first 25% were discarded as burn-in. The “allcompat” tree (majority rule plus compatible groups) that results from the analysis includes evolutionary rate estimates for each node and terminal branch. Beck and Lee ([51]:3) advocated for the discussion of the median evolutionary rate rather than the mean evolutionary rate, a protocol followed here. Rates for each node were converted to absolute rates of change per site per Ma by multiplying individual median node rates by the overall median rate for the entire analysis, and then multiplying by 100 to get the median absolute rate in percentage change/Ma. The input dataset for MrBayes is available as S4 Dataset.

### Parsimony analysis

Parsimony analysis is computationally simpler than Bayesian methods, which have only recently been possible to implement on most personal computers. Here, we perform a parsimony analysis to allow for direct comparisons with previously published analyses of hyaenodont relationships [3, 6, 10, 17, 21, 27–30]. The software package Tree Analysis using New Technology (TNT) version 1.1 [57] was used to conduct a maximum parsimony analysis of the character-taxon matrix using the traditional search heuristic algorithm across 10,000 replicates, random addition sequence and tree bisection and reconnection (TBR) branch swapping. Ten trees per TBR replicate were held for the analysis with consistency index (CI) and retention index (RI) values calculated in STATS.RUN for TNT. Bremer [58] support for nodes recovered in the parsimony analysis was calculated in TNT, and node support was also calculated by running 10,000 bootstrap pseudoreplicates [59]. Adams consensus and agreement subtrees were calculated in PAUP 4.0 [60]. The input file for the maximum parsimony analysis is available as S6 Dataset.

### Multivariate morphometric analysis of hyaenodont and carnivoran distal humeri

Three isolated left humeri from L-41 are referred to *Masrasector* in this study. In addition to comparative anatomical interpretations of the specimens, we conducted a discriminant function analysis (DFA) to infer the locomotor preferences of *Masrasector* and other hyaenodonts, using an ecologically diverse comparative sample of carnivorans. While the precise relationship between Carnivora and the major clades in Hyaenodonta remains largely untested [7, 8, 55, 61, 62], the size ranges exhibited by species in Carnivora and Hyaenodonta are broadly comparable, and both clades were shaped by selective pressures that resulted in convergent carnivorous dentitions. As carnivores, hyaenodonts likely evolved comparable locomotor adaptations to those seen among carnivorans today. This analysis (1) tests the hypothesis that carnivorans and hyaenodonts overlap in distal humeral morphospace sufficiently to serve as adequate comparative models for one another; (2) identifies which specimens included in the extant sample best approximate the morphology of the *Masrasector* humeri in the resulting morphospace; and (3) uses the discriminant functions derived from the analysis to reconstruct the likely locomotor category for *Masrasector* as well as the other hyaenodonts included in the analysis. Phylogenetic comparative methods were not used in this analysis because the relationship between Hyaenodonta and Carnivora is currently poorly understood, but the raw data are reported so that future analyses can incorporate phylogenetic comparative methods into an understanding of hyaenodont postcranial diversity.

**Fig 2:**
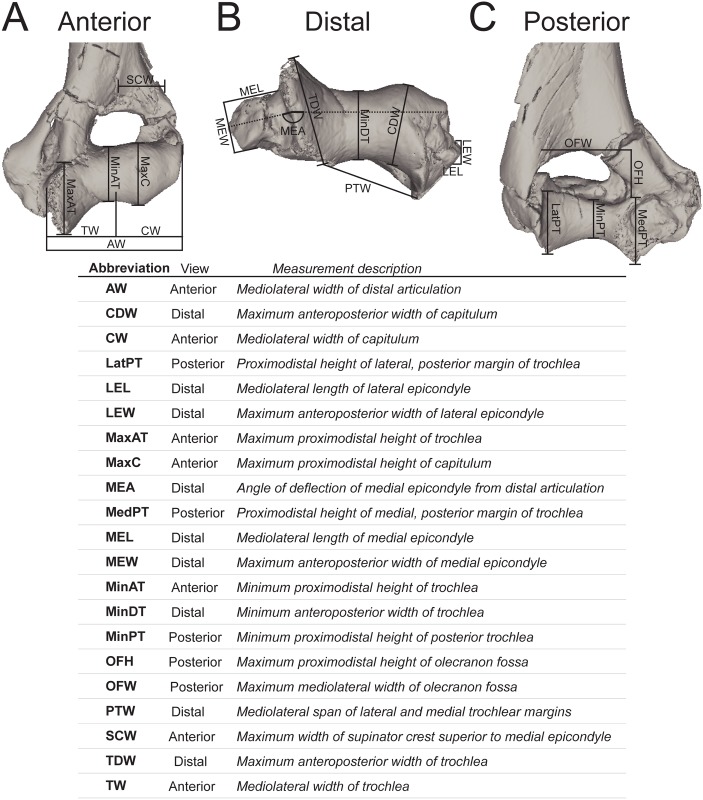
Humerus measurements for DFA. Humerus measurements used in the Discriminant Function Analysis. Collected from digital photographs in (**A)** anterior view, (**B)** distal view, and (**C)** posterior view. Abbreviations for each measurement are listed with the anatomical view they were collected from and a description of the measurement. Measurements illustrated on DPC 10831.

Twenty linear measurements and one angular measurement were collected from photographs of 155 carnivoran humeri representing 55 species and 12 hyaenodont specimens (Fig 2). The photographs were taken with a Nikon D3300 camera with an AF-S DX Nikkor 18–55mm lens that was mounted on a photography copy stand. Images were captured at least 60 cm from each specimen in anterior, posterior, and distal views. In anterior view, the specimens were oriented with the plane of the lens parallel to the plane formed by the anterior border of the deltopectoral crest, and the anterior-most points of the capitulum and trochlea; in posterior view, parallel to the posterior-most point of the medial and lateral trochlear margin and the proximal-most point of the humeral head; and in distal view, perpendicular to the anterior deltopectoral crest or parallel to the anterior and posterior points of the trochlea.

Linear measurements were recorded to the nearest 0.01 mm and angular measurements to the nearest 0.01 degree using ImageJ [40]. The measurements were selected to capture the morphology of the distal humerus with some apparent redundancies (e.g., the measurements “MaxAT” and “MedPT”) so some measurements can be removed in future analyses of more fragmentary fossil material that does not record the complete distal humerus. Each linear measurement was divided by the geometric mean of all measurements for that specimen [63] and the results were transformed by their z-scores. Each measurement used in the DFA is equal to ((measurement/geometric mean)-mean of measurement)/standard deviation). Comparing variance from the mean rather than the direct measurements, the angle of the medial epicondyle could be incorporated into the discriminant function analysis without adjusting for like units.

The comparative sample draws from all major carnivoran clades and broadly samples the locomotor diversity of Carnivora. Each species was placed in a stereotypical locomotor category: “Arboreal,” “Scansorial,” “Terrestrial,” “Fossorial,” or “Semiaquatic.” These categories follow Samuels et al. [64]. “Arboreal” species rarely forage or shelter on terrestrial substrates and habitually climb and forage in the trees (e.g., kinkajou, red panda, binturong). “Scansorial” species climb effectively and regularly exhibit this behavior, but do not forage exclusively in trees (e.g., margay, raccoon, coati). “Terrestrial” species primarily forage and shelter on the ground, rarely climbing or swimming or excavating complex burrows (e.g., hyenas, skunks, dogs). “Fossorial” species habitually build and shelter in burrows that are part of extensive networks (e.g., badgers, meerkats). “Semiaquatic” species primarily swim while foraging and sheltering near water (e.g., river otter, mink). Each species was assigned to a locomotor category based on categories assigned by Van Valkenburgh [65] and Samuels et al. [64], and in consultation with Nowak [66] and Myers et al. [67].

Mammals are capable of a wide variety of locomotion and it is often difficult to identify a discrete category for a species. It is particularly difficult to stereotype many carnivorans, which may vary their locomotor preferences by habitat, foraging patterns, and sheltering options [68, 69]. For example, the grey fox (*Urocyon cinerargenteus*) is capable of climbing trees to escape predators and to forage, but it also forages capably in open environments [70], making it more appropriately categorized as “Scansorial” in some environments and “Terrestrial” in others. The locomotor categories utilized in this study are not mutually exclusive, even when a species is in its typical habitat. For example, semiaquatic mammals frequently excavate burrows along the banks of waterways, making river otters and mink both semiaquatic and fossorial [66]. River otters use their hind limbs and lumbar extension and flexion to generate thrust in the water, while their forelimbs are used to steer, or are tucked against the body while swimming [71]. The fossorial behaviors of river otters rely on large muscle attachments anchored to their humeri to support aggressive scratch digging [72, 73]. These qualifications should be kept in mind when interpreting the discriminant function categorical data presented here that are limited to distal humeral morphology.

When possible, left humeri were photographed for measurement, and when the sex of the specimen was known, at least one specimen of each sex was collected. Specimen measurements were kept distinct, rather than averaged into species means, because some species are only represented by a single specimen in the dataset (e.g., *Cryptoprocta ferox*) and the fossil specimens are also single samples.

Discriminant function analysis was conducted in the statistical package IBM SPSS 22 to determine the locomotor classification of the hyaenodont humeri. Using the Classify, Discriminant protocol, the prior probability that each specimen belonged to a given locomotor category was set to equal and the analysis used the within-group covariance matrix. The discriminatory effectiveness of the discriminant functions was evaluated by considering the accuracy of each successful classification of a specimen with a known locomotor behavior. Leave-one-out cross-validation was used to further test the predictive utility of the discriminant functions. The resulting discriminant function scores were visualized in PAST 3.04 [74] and Euclidean distances between the DF scores of each specimen were also calculated in PAST 3.04.

## Results

### Systematic paleontology

### Systematic hierarchy

### Hyaenodonta Van Valen, 1967 [75] sensu Solé et al., 2015 [30]

### Hyainailouroidea Borths, Holroyd, and Seiffert, 2016 [9]

### Teratodontinae Savage, 1965 [21]

#### Emended diagnosis of Teratodontinae

Modified from Solé et al. [22]. Defined here by the node that represents the common ancestor of *Furodon* and *Teratodon*. Differs from Hyainailourinae by having distinct hypoconids and hypoconulids and wide and deep talonid basins on lower molars, rather than having talonid basins that are narrower than trigonids, with indistinct hypoconids and hypoconulids; lower molar entocristids lingually close the talonids rather than leave the talonids open lingually; connate metaconids on at least M_1_ and M_2_ (metaconids and talonids are greatly reduced to absent on M_3_ in some Miocene teratodontines) rather than metaconids forming indistinct ridges; divergent metacones and paracones on M^1^ and M^2^, rather than almost entirely fused; M^1, 2^ metacones and paracones subequal in height, or metacone taller, rather than paracones taller than metacones; internal choanae open just distal to M^3^, with palatines lateral to pharyngeal passage rather than internal choanae open far distal to M^3^ with palatines forming long, closed narial tube; nuchal crest narrows slightly from apex to foramen magnum rather than tapering to form a narrow nuchal wedge above foramen magnum. Differs from Apterodontinae by having more buccolingually compressed rather than connate M^1–2^ paracones and metacones; M^1–2^ paracones and metacones subequal or metacones taller than paracones rather than paracones taller than metacones; M^1–2^ metastyles mesiodistally elongate and buccolingually compressed rather than mesiodistally short; retaining connate M_1_ metaconid rather than a vestigial ridge or no metaconid; having palatines diverge distal to M^3^ rather than forming elongate narial tube.

#### Included genera

*Anasinopa* Savage, 1965 [21]; *Brychotherium* Borths et al., 2016 [9]; *Buhakia* Morlo et al., 2007 [26]; *Dissopsalis* Pilgrim, 1910 [76]; *Furodon* Solé et al., [22]; *Glibzegdouia* Crochet et al., 2001 [77]; *Masrasector* Simons and Gingerich, 1974 [23]; *Teratodon* Savage, 1965 [21].

#### Biogeographic and temporal range

Early–middle Eocene (late Ypresian) of Afro-Arabia to late middle Miocene of Afro-Arabia and Asia.

### Genus *MASRASECTOR* Simons and Gingerich, 1974 [23]

#### Type species

*Masrasector aegypticum* Simons and Gingerich, 1974 [23]

#### Other included species

*Masrasector ligabuei* Crochet et al., 1990 [24]

#### Emended diagnosis

Small hyaenodont with dental formula I^3^/_?_, C^1^/_1_, P^4^/_4_, M^3^/_3_. Differs from *Brychotherium* by being smaller; protocone tall and prominent rather than low-crowned on P^4^; metastyle short rather than tall and buccolingually compressed on P^4^; M^1, 2^ paracone and metacone elliptical in cross section, rather than buccolingually compressed and blade-like; M^1, 2^ paracone and metacone about the same height, rather than metacone taller and mesiodistally longer than the paracone; short metastyle on M^2^ that is the mesiodistal length of the metacone, rather than a metastyle that is equal in mesiodistal length to the paracone and metacone base; M_1, 2_ trigonids only twice rather than three times the height of the talonid. Differs from *Glibzegdouia* by being smaller; metaconids are lower than the paraconids on all molars, rather than only on M_2,– 3_; entoconids distinct on M_1, 2_ rather than entoconids indistinct and blending with the entocristids; smooth connection between the entocristid and the base of the metaconid on the lower molars, rather than having a deep notch formed along the entocristid; anterior keels extend to bases of the protoconids rather than only the paraconids; talonids occupy ~50% or less of the mesiodistal length of each molar, rather than ~60% of molar length. Differs from *Furodon* by having P_4_ relatively shorter with equilateral preparacristid and postparacristid rather than P_4_ preparacristid inclined distally; trigonid height relatively closer to talonid height rather than trigonid substantially taller than talonid; deep, basined talonid enclosed lingually by entocristid rather than shallow and open lingually. Differs from *Anasinopa* by being smaller; M^1, 2^ trigon basin mesiodistal length and buccolingual width subequal rather than buccolingually wider than mesiodistal length; hypoconid short on P_4_, rather than tall and close to half the height of the protoconid; tall rather than low and mesiodistally elongate lower molar metaconids, particularly on M_3_; talonid basined rather than lingually open on M_3_. Differs from *Dissopsalis* by being smaller; M^1, 2^ paracones and metacones are similar in height, rather than M^1, 2^ metacones much taller and mesiodistally longer than the paracones; talonid forms a mesiodistally long basin on M_3_, rather than being reduced with indistinct cusps; lower molar postparacristids and preprotocristids form an oblique angle relative to the long axis of the mandibular corpus, rather than forming an angle that nearly parallels the long axis of the mandibular corpus. Differs from *Teratodon* by being smaller; shallow rather than deeply embayed ectoflexus on M^2^; premolars buccolingually narrow rather than massive and bulbous. Differs from *Metasinopa* by being smaller; lower molar metaconids pronounced and connected to paraconids, rather than small; defined lower molar talonid cusps rather than indistinct cristids surrounding the talonid basin.

### *Masrasector nananubis*, sp. nov

urn:lsid:zoobank.org:act:3260E11C-B977-494D-805E-F62EB1709C9E

Figs 3, 4, 5, 6, 7, 8, 9, 10, 11, 12, Tables 1 and 2

**Fig 3:**
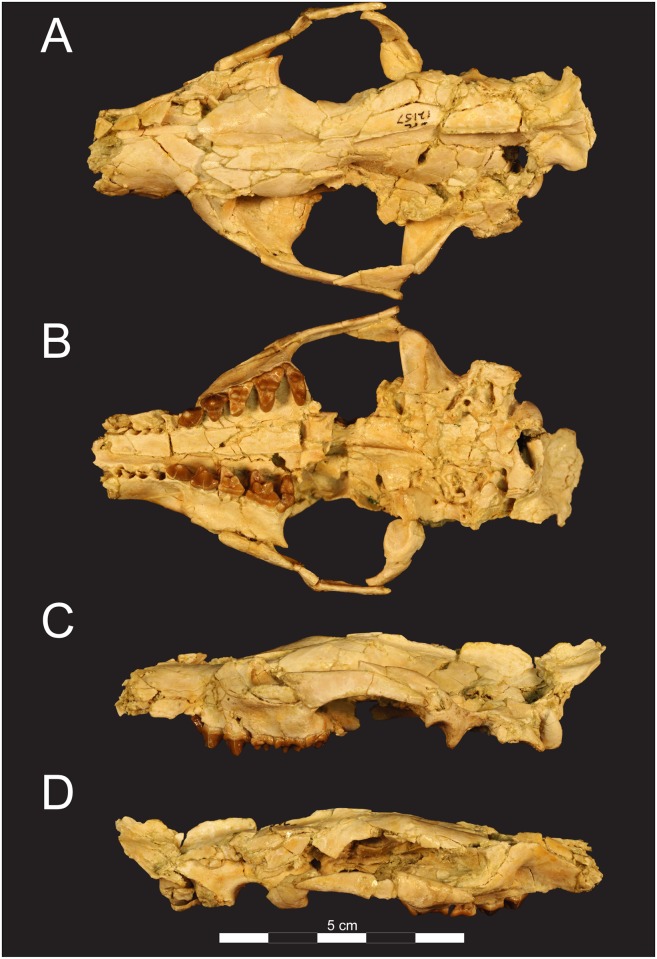
Cranium of *Masrasector nananubis* (DPC 12157). Cranium of *Masrasector nananubis* nov. sp. (DPC 12157 with alveoli for P^1, 2^, complete P^3^–M^3^) in (**A)** dorsal view, (**B)** ventral view, (**C)** left lateral view, (**D)** right lateral view. Rostrum points left in (**A)**, (**B)**, and (**C)** and right in (**D)**.

**Fig 4:**
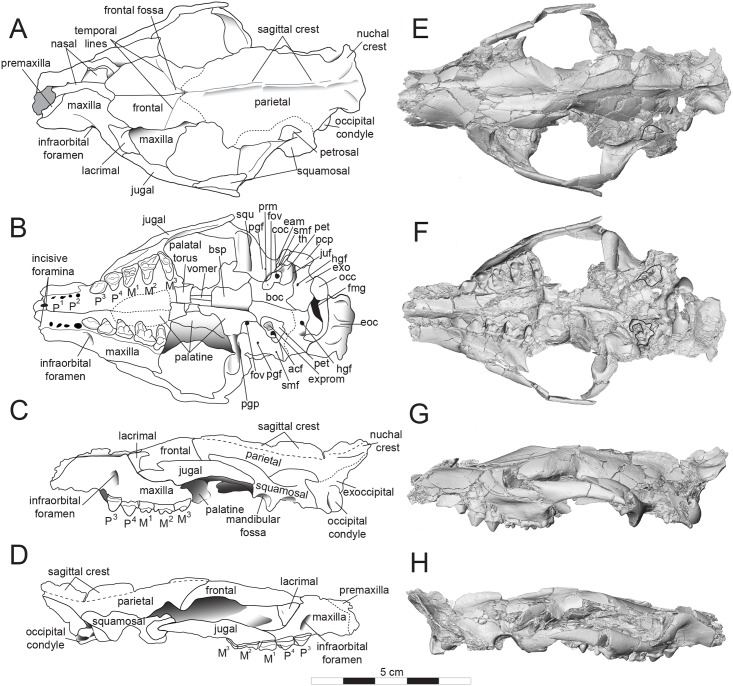
Sketch and model of *Masrasector nananubis* cranium (DPC 12157). Sketch of cranium *Masrasector nananubis* sp. nov. (DPC 12157 with alveoli for P^1, 2^, complete P^3^–M^3^) in (**A)** dorsal view, rostrum points left, (**B)** ventral view, rostrum points left, (**C)** left lateral view, rostrum points left, (**D)** right lateral view, rostrum points right. Dotted lines indicate uncertain sutures or root of sagittal crest. Digital model of cranium in (**E)** dorsal, (**F)** ventral, (**G)** left lateral, and (**H)** right lateral views. The digital model was generated in Avizo and is available on Morphosource. Abbreviations: **bsp**, basisphenoid; **eam**, external auditory meatus; **eoc**, external occipital crista; **exo**, exoccipital; **exprm**, exposed promontorium; **fmg**, foramen magnum; **fov**, foramen ovale; **hgf**, hypoglossal foramen; **juf**, jugular foramen?; **occ**, occipital condyle; **pcp**, paracondylar process; **pgf**, post glenoid foramen; **pgp**, postglenoid process; **prm**, promontorium; **smf**, stylomastoid foramen; **squ**, squamosal; **th**, tympanohyal.

**Fig 5:**
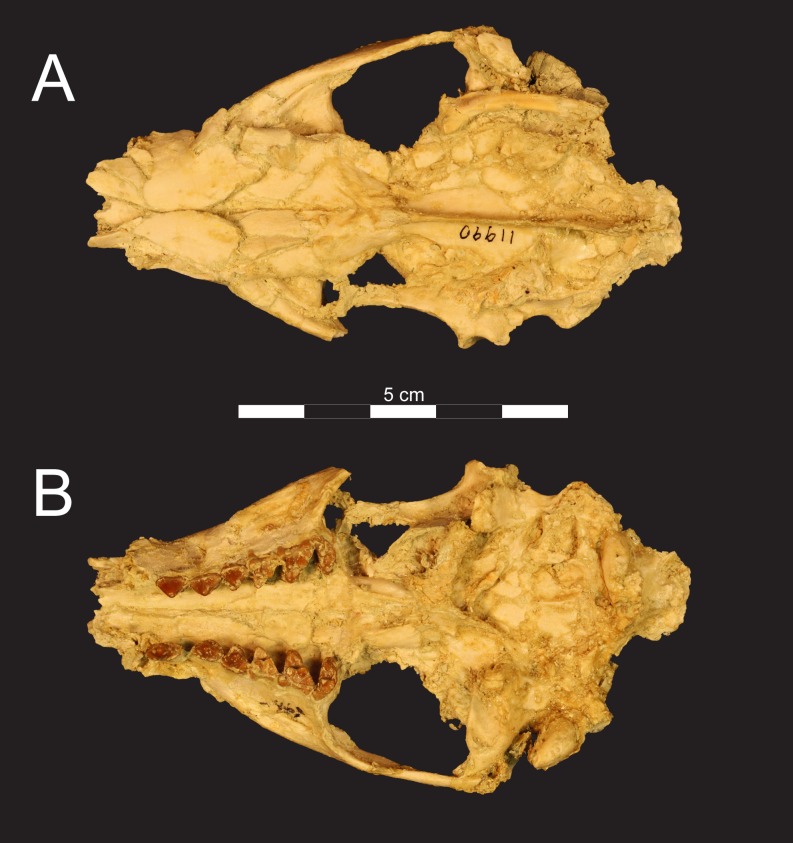
Cranium of *Masrasector nananubis* (DPC 11990). Cranium of *Masrasector nananubis* sp. nov. (DPC 11990 with P^2^–M^3^) in (**A)** dorsal view, rostrum points left, (**B)** ventral view, rostrum points left.

**Fig 6:**
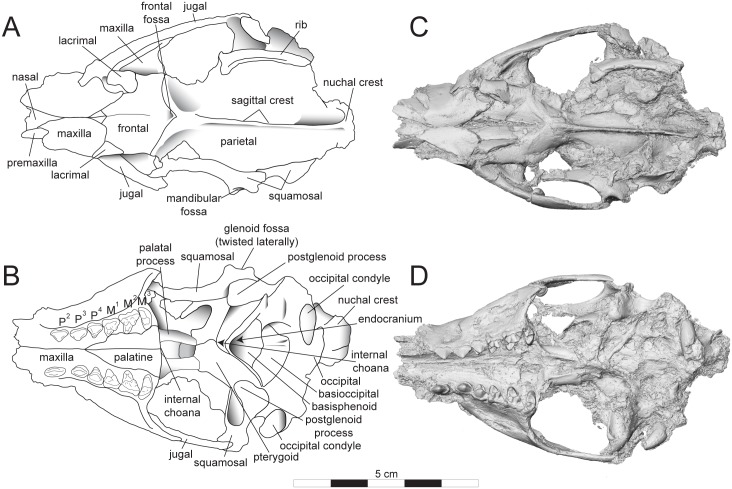
Sketch and digital model of *Masrasector nananubis* cranium (DPC 11990). Sketch of cranium of *Masrasector nananubis* sp. nov. (DPC 11990 with P^2^–M^3^) in (**A)** dorsal view, rostrum points left, (**B)** ventral view, rostrum points left. Digital model in (**C)** dorsal, and (**D)** ventral view is an isosurface rendering of the specimen generated using Avizo and is available on Morphosource.

**Fig 7:**
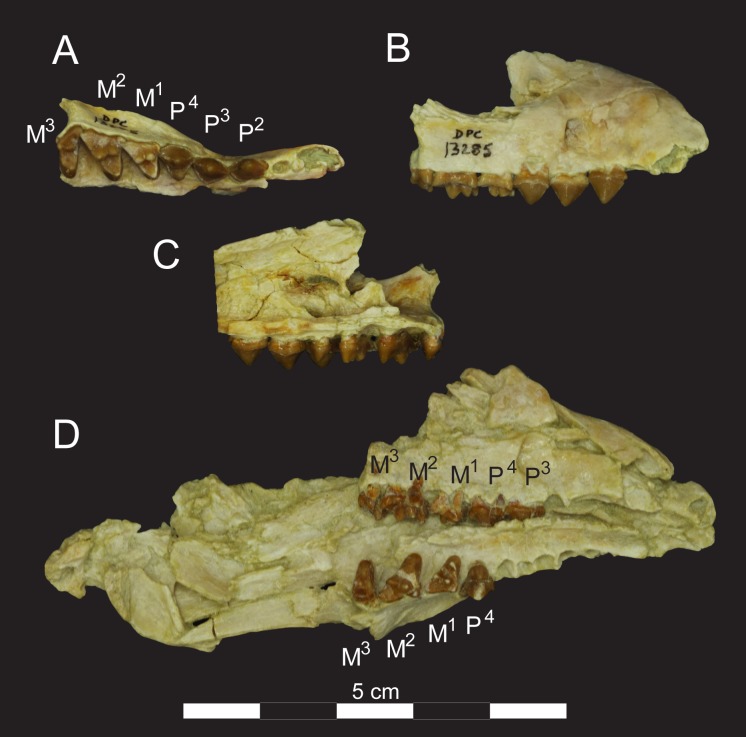
Right maxilla (DPC 13285) and rostrum (DPC 8276) of *Masrasector nananubis*. Right maxilla of *Masrasector nananubis* sp. nov. (DPC 13285 with P^2^–M^3^) in (**A)** occlusal view, buccal side to top and mesial to right, (**B)** lateral/buccal view, mesial to right, (**C)** lingual/medial view, mesial to left, (**D)** rostrum of *Masrasector nananubis* sp. nov. (DPC 8276), rostrum points right, left dentition in occlusal view and right dentition in buccal view.

**Fig 8:**
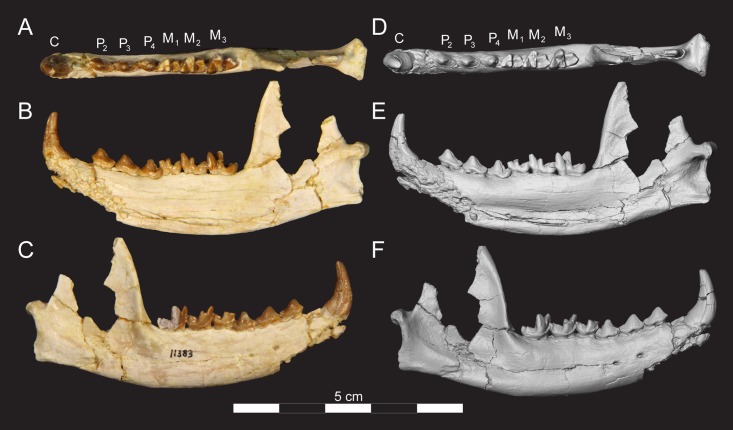
Right dentary of *Masrasector nananubis* sp. nov., (CGM 83736 holotype). DPC 11383 with C, alveoli for P_1_, P_2_–M_3_ in (**A)** occlusal view, buccal to top, mesial to left, (**B)** lingual view, mesial to left, (**C)** buccal view, mesial to right. Digital model in (**D)** occlusal, (**E)** lingual, and (**F**) buccal view is an isosurface rendering of the specimen generated using Avizo and is available on Morphosource.

**Fig 9:**
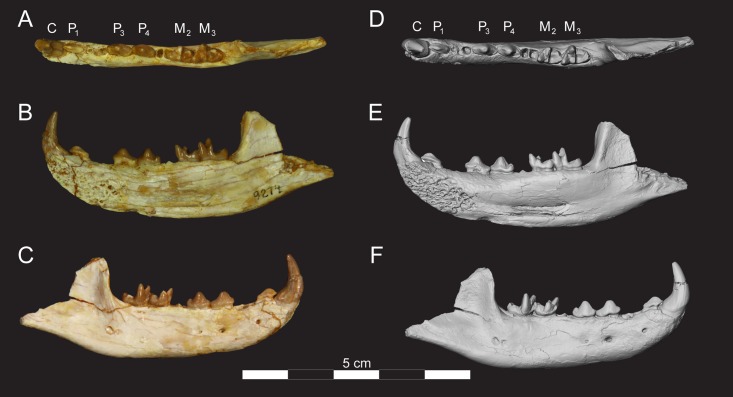
Right dentary of *Masrasector nananubis* sp. nov. (DPC 9274). DPC 9274 with P_1_, alveoli for P_2_, P_3_–P_4_, alveoli for M_1_, M_2_–M_3_ in (**A)** occlusal view, buccal to top, mesial to left, (**B)** lingual view, mesial to left, (**C)** buccal view, mesial to right. Digital model in (**D)** occlusal, (**E)** lingual, and (**F**) buccal view is an isosurface rendering of the specimen generated using Avizo and is available on Morphosource.

**Fig 10:**
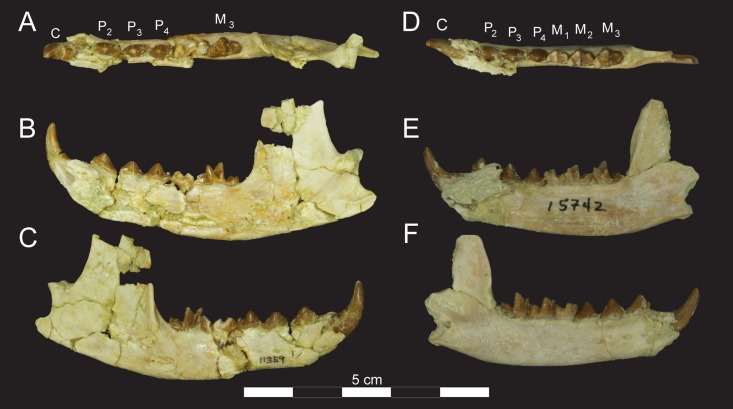
Right dentaries of *Masrasector nananubis* sp. nov. (DPC 11359 and DPC 15742). DPC 11359 with P_2_–P_4_, M_3_ in (**A)** occlusal view, buccal to top, mesial to left, (**B)** lingual view, mesial to left. Portions of M_1_–M_2_ are preserved but fragmentary, (**C)** buccal view, mesial to right. DPC 15742 with P_2_–M_3_ in (**D)** occlusal view, buccal to top, mesial to left, (**E)** lingual view, mesial to left, (**F)**, buccal view, mesial to right.

**Fig 11:**
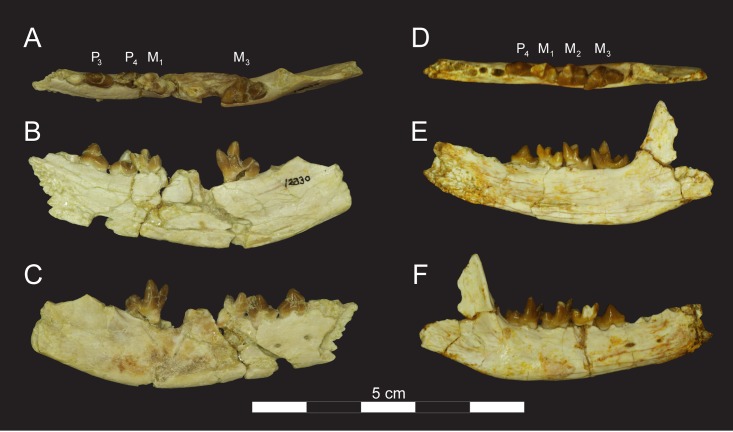
Right dentaries of *Masrasector nananubis* sp. nov. (DPC 12330 and DPC 12524A). DPC 12330 with P_3_–M_1_, M_2_ in (**A)** occlusal view, buccal to top, mesial to left, (**B)** lingual view, mesial to left, (**C**) buccal view, mesial to right. DPC 12524A with alveoli for P_2, 3_, P_4_–M_3_ in (**D**) occlusal view, buccal to top, mesial to left, (**E)** lingual view, mesial to left, (**F)** buccal view, mesial to right.

**Fig 12:**
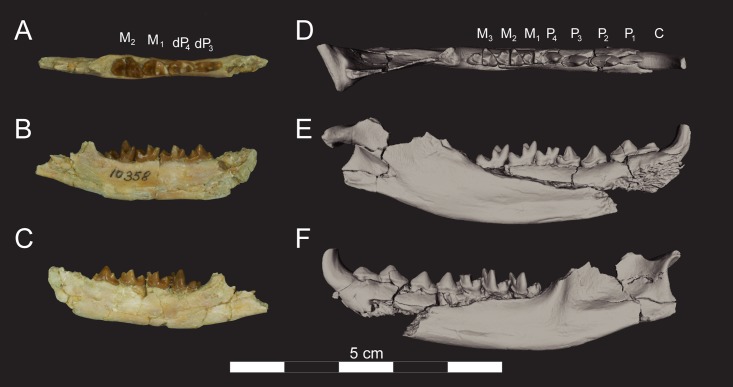
Left dentaries of *Masrasector nananubis* sp. nov. (DPC 10358 and DPC 15211). DPC 10358 with dP_3, 4_, M_1, 2_ in (**A)** occlusal view, buccal to top, mesial to right, (**B)** lingual view, mesial to right, (**C)** buccal view, mesial to left. Digital model of DPC 15211 generated in Avizo with C, P_1_–M_3_ in (**D)** occlusal view, buccal to top, mesial to right, (**E)** lingual view, mesial to right, (**F)** buccal view, mesial to left. Model is available on Morphosource.

**Table 1:** Average measurements of the upper dentition of *Masrasector nananubis*.

Tooth	n	Length				Width		Paracone Height		Paracone Length		Parastyle Length		Metacone Height		Metacone Length		Metastyle Length		Protocone angle	
		Average	Std. Dev.	Min. Ob.	Max. Ob.	Average	Std. Dev.	Average	Std. Dev.	Average	Std. Dev.	Average	Std. Dev.	Average	Std. Dev.	Average	Std. Dev.	Average	Std. Dev.	Average	Std. Dev.
P^2^	3	5.16	0.51	4.67	5.69	2.45	0.28	3.09	0.22	4.54	0.43	–	–	–	–	–	–	0.84	0.01	–	–
P^3^	7	4.98	0.25	4.63	5.4	3	0.63	2.72	0.36	4.24	0.17	–	–	–	–	–	–	0.78	0.2	95.84	3.4
P^4^	7	4.6	0.31	4.07	4.91	4.5	0.71	2.24	0.63	3.17	0.87	0.6	0.11	–	–	–	–	0.96	0.23	92.95	1.38
M^1^	8	4.72	0.6	4.22	6.11	4.56	0.63	1.36	0.43	1.72	0.59	0.98	0.13	1.3	0.44	1.43	0.29	1.89	0.56	95.02	2.55
M^2^	8	5.87	0.57	5.31	7.14	5.67	0.73	1.51	0.32	1.56	0.19	1.16	0.26	1.67	0.45	1.86	0.21	2.47	0.64	95.63	2.96
M^3^	8	2.71	0.26	2.22	3.04	6.31	1.08	1.1	0.35	1.6	0.14	1.7	0.37	–	–	–	–	0.75	0.18	91.67	3.2

Average (±standard deviation); **Max. Ob.,** maximum observed; **Min. ob**., minimum observed

**Table 2:** Average measurements of the lower dentition of *Masrasector nananubis*.

Tooth	n	Length				Max Trigonid Length		Max Talonid Width		Max Trigonid Width		Max Talonid Width		Protoconid Height		Talonid Height	
		Average	Std. Dev.	Min. Ob.	Max. Ob.	Average	Std. Dev.	Average	Std. Dev.	Average	Std. Dev.	Average	Std. Dev.	Average	Std. Dev.	Average	Std. Dev.
dP_3_	1	5.27	–	–	–	4.16	–	0.82	–	1.51	–	1.21	–	2.62	–	0.99	–
dP_4_	1	4.98	–	–	–	2.88	–	1.74	–	2.11	–	2.11	–	2.55	–	2.05	–
C	5	4.84	0.59	4.13	5.62	–	–	–	–	3.64	0.59	–	–	8.56	1.78	–	–
P_1_	2	4.53	0.14	4.43	4.63	3.35	0.13	1.18	0.29	1.74	0.00	1.25	0.06	1.48	0.02	0.68	0.19
P_2_	4	5.41	0.11	5.32	5.56	3.97	0.18	1.46	0.23	2.53	0.29	1.80	0.21	3.04	0.36	1.28	0.10
P_3_	6	5.21	0.27	4.94	5.68	3.95	0.32	1.32	0.20	2.39	0.28	1.90	0.17	3.16	0.68	1.43	0.33
P_4_	7	4.88	0.33	4.48	5.31	3.66	0.27	1.23	0.21	2.52	0.24	1.93	0.15	3.39	0.33	1.89	0.22
M_1_	6	4.45	0.37	3.92	4.78	2.41	0.29	1.96	0.21	2.78	0.33	2.42	0.13	3.02	0.54	2.21	0.23
M_2_	6	5.28	0.35	4.82	5.70	2.72	0.24	2.46	0.13	3.40	0.28	2.76	0.17	3.83	0.27	2.03	0.19
M_3_	7	6.20	0.38	5.64	6.67	3.36	0.34	2.88	0.25	3.84	0.34	2.47	0.33	4.64	0.53	2.12	0.44

Average (±standard deviation); **Max. Ob.,** maximum observed; **Min. ob**., minimum observed

#### Etymology

Meaning “little Anubis,” from Greek νάνος (nannos), meaning little, and Anubis (Ἄνουβις) the Greek name for the jackal-headed ancient Egyptian god of embalming who protected tombs and led souls through the underworld.

#### Holotype

CGM 83736 (formerly DPC 11383), right dentary with canine, P_2_–M_3_.

#### Referred material

CGM field number 96–161, rostrum fragment with P^3^–M^2^; CGM field number 95–281, dentary with P_4_–M_1_; CGM field number 95–109, isolated M^2^; DPC 7704, left dentary with P_2_–M_3_; DPC 8276, rostrum and palate with P^3^–M^3^; DPC 9274, right dentary with canine, P_1_, alveoli for P_2_, P_3_–P_4_, alveoli for M_1_, M_2, 3_; DPC 10358, left dentary with dP_3_, dP_4_, M_1, 2_; DPC 11383, right dentary with C, alveoli for P_1_, P_2_–M_3_; DPC 11359, right dentary with canine, P_1–4_, M_3_; DPC 11990, cranium with P^2^–M^3^; DPC 12157, cranium with alveoli for P^1, 2^, P^3^–M^3^; DPC 12330, right dentary with P_3_–M_1, 2_; DPC 12524A, right dentary with alveoli for P_2, 3_, P_4_–M_3_; DPC 13285, rostral fragment with P^2^–M^3^; DPC 15211, left dentary with C, P_1_–M_3_; DPC 15742, right dentary with canine, P_2_–M_3_; DPC 10831, left distal humerus; DPC 15436, left distal humerus; DPC 11670, left distal humerus.

#### Type locality

Jebel Qatrani Locality 41 (L-41), Jebel Qatrani Formation, Fayum Depression, Egypt. L-41 is approximately 14.5 km west of Qasr el-Sagha Temple, and 2 km north of the contact between the Qasr el-Sagha Formation and the Jebel Qatrani Formation

#### Age

Late Eocene, latest Priabonian, ~34 Ma [34].

#### Geographic distribution

Type locality only.

#### Diagnosis

Differs from *M*. *aegypticum* by being smaller; P_3_ width buccolingually narrow, rather than P_3_ width more than half its mesiodistal length; hypoconulids developed rather than indistinct on lower molars; lower molar talonids buccolingually narrower than trigonids, rather than buccolingually broad with width subequal to mesiodistal length; entocristids buccolingually narrower. Differs from *M*. *ligabuei* by being smaller; protocone on M^1^ rises to same plane as divergence between paracone and metacone, rather than projecting beyond notch between paracone and metacone; narrow trigon basin, rather than subequal to buccolingual width of protocone; long M_3_ talonid compared to trigonid, rather than trigonid ~60% of mesiodistal length of M_3_; thin rather than mesially projecting anterior keels on lower molars.

### Description of the cranium

The description of the cranium is based on three cranial specimens from L-41. Specimens from L-41 are remarkable for their completeness, but are usually heavily deformed by post-depositional processes. DPC 8276 preserves the palate and rostrum (Fig 7). The entire specimen is dorsoventrally crushed and the right portion of the rostrum is internally rotated so the right tooth row is in buccal view when the left tooth row is in occlusal view. DPC 11990 preserves the entire cranium but is crushed dorsoventrally (Figs 5 and 6). The dentition and right and left portions of the rostra are slightly internally rotated. DPC 12157 was only slightly crushed dorsoventrally, preserving portions of the left palate, rostrum, cranial vault, and basicranium (Figs 3 and 4). Minor reconstruction took place in preparing DPC 12157, the least distorted cranium of *Masrasector nananubis* known.

#### Rostrum

The premaxilla is fragmentary in all specimens, but it can be reconstructed as a thin splint of bone that traced the lateral portion of the external nasal aperture and followed the curvature of the root of the canine to the lateral margin of the nasal bones (Fig 4). The rostrum is narrow and tubular between the root of the canine and the mesial root of P^3^. Dorsal to the mesial root of P^3^ is the large infraorbital foramen (~3.2 mm diameter), which is close in diameter to the buccolingual width of P^3^. From the distal root of P^3^ the palate and maxilla flare laterally. The maxilla does not form any portion of the orbital margin. The nasals are slightly retracted over the nasal aperture. The nasal-maxilla sutures parallel the median internasal suture to about the position of the infraorbital foramen where the nasals, like the palate, flare laterally. The nasal-frontal suture is V-shaped with the most distal point of the nasal at the midline, at the same anteroposterior point as the distal root of P^4^. The suture trends laterally and rostrally to a point dorsal to the mesial root of P^4^. The medial aspect of the maxilla is visible on DPC 13285. The maxillary recess is deep and the crista semicircularis, which defines the rostral extent of the maxillary recess, extends from a point dorsal to the distal root of P^4^ to the mesial root of P^3^.

#### Palate

The incisive foramina are elliptical, with their long axes parallel to the palatal suture and a mediolateral width subequal to that of the alveolus of the distal root of P^1^. The palatal processes of the maxillae are narrow between the incisors and the mesial root of P^3^, and then flare laterally posterior to the mesial root of P^3^. The palatine-maxilla suture is placed at the same anteroposterior point as the protocone of P^4^. The palatal processes of the maxilla project slightly beyond the protocones of the molars to the distal margin of the hard palate. The maxilla is embayed between the protocones of the maxilla to accommodate the trigonids of the lower molars. The horizontal plates of the palatine bones are punctated by many small palatal foramina. Along the distal edge of the horizontal plate, each palatine forms a thick palatal torus and the perpendicular plates of the palatines frame the channel of the internal choana. Posterior to the hard palate, the palatines are unfused, leaving the thin vomer visible. The palatines form a broad wall perpendicular to the palate for the origin of the pterygoid musculature.

#### Orbit

The orbit is framed by the frontal along its dorsal margin, the lacrimal along its anterior margin, and the jugal along its ventral margin (Fig 4). The frontal has a slight eminence defining the posterior aspect of the orbit, but no distinct postorbital process is present. The frontal-lacrimal suture extends from within the orbit onto the face, along the facial process of the lacrimal. The lacrimal projects from the anterior orbital margin dorsal to the mesial root of M^1^ to the mesial root of P^4^ (~3.0 mm). The lacrimal is also mediolaterally wide, with a broad maxilla-lacrimal suture tracing the anterior origin of the zygomatic arch (~7.3 mm). The lacrimal foramen is just inside of the anterior orbital margin. The maxillary foramen is enclosed by the maxilla ventral to the lacrimal and inside the orbit. The orbital mosaic and foramen that perforate the cranium medial to the zygomatic arch are obscured except for the right ethmoid foramen. The ventral margin of the orbit is formed by the jugal, which forms a long suture with the maxilla that parallels the palate. The jugal is dorsoventrally wide distal to the zygomatic process of the maxilla, as dorsoventrally wide as the mesiodistal length of M^2^. The jugal almost reaches the glenoid fossa. The root of the zygomatic process of the squamosal is rostrocaudally wide, forming a broad dorsal surface over the glenoid fossa. The posterior margin of the squamosal bears a thin ridge that curves rostrally as it twists into the zygomatic process and contributes to the squamosal-jugal suture. The zygomatic process of the squamosal extends in a rostral direction to approximately the same transverse plane as the origin of the sagittal crest. The overlapping jugal and squamosal maintain a consistent dorsoventral width for the zygomatic arch along its rostrocaudal length.

#### Cranial vault and occipital region

The frontals each have distinct V-shaped sutural surfaces for the nasals and the frontal processes of the maxillae that trend posteriorly to meet the lacrimal. The frontal forms broad portions of the dorsal and medial parts of the orbit, bulging slightly at the postorbital peak. There is a slight depression rostral to the sagittal suture and the origin of the sagittal crest (a “frontal fossa”). The outline of the frontal-parietal suture is difficult to follow. The posterior and lateral margins of the frontal rise to distinct temporal lines that trace the broad origin of the anterior portion of the temporalis muscle, which originates from the sagittal suture along the parietals and sweeps rostrally to the postorbital peak of the frontal. The sagittal crest is prominent, about the same dorsoventral height as the crown of P^3^. On the posterior portion of the skull, the sagittal crest maintains a constant height but the parietals curve ventrally toward the paroccipital processes. The sagittal crest meets the nuchal crest caudally. The nuchal crest is broad and fan-like, spreading laterally above the nuchal line. The nuchal crest is depressed ventrally in all specimens, but it appears to have a slight caudal inclination, based on the angle formed with the dorsal rim of the foramen magnum. Lateral to the sagittal crest are shallow depressions and the lateral margins of the nuchal crest curve posteriorly, wrapping toward the occipital condyles. The lateral border of the nuchal crest continues as a thin line to the paroccipital process. The posterior surface of the nuchal crest (dorsal portion of the supraoccipital) has a shallow fossa dorsal to the foramen magnum for the insertion of suboccipital musculature. The external occipital crista runs from the median apex of the nuchal crest to the dorsal margin of the foramen magnum. The occipital condyles are ellipsoidal with a thin mediolateral diameter and long dorsoventral axis. The occipital condyles frame the lateral portions of the foramen magnum, the ventral margin of which is traced by thick crests that meet at a point rostral to its dorsal margin (Figs 4 and 6).

#### Basicranium

Crushing and distortion of the morphologically complex basicranial region complicates interpretation, and clarification of most details must await the recovery of unbroken and undistorted material. There is no clear evidence for the presence of an ossified auditory bulla enclosing the petrosal. Broken remnants of the petrosal are preserved and are best seen on DPC 12157, particularly on the left side. Much of the promontorial housing is missing, and a conspicuous “foramen” (created by breakage of the promontorium and cochlea) that is visible in ventral view appears to be an approximately transverse cross-section through the base of the first turn of the cochlea, partially continuous with remnants of the fenestra cochleae posteriorly and fenestra vestibuli dorsally and anteriorly. A thin splint of bone protrudes ventromedially from the posterior aspect of the squamosal roof of the external auditory meatus over a broad gutter for the facial nerve (CN VII) and is likely to represent the tympanohyal. Breakage of the floor of the gutter has exposed part of the lateral semicircular canal. On the endocranial surface of the petrosal, the bony walls of the internal acoustic meatus are largely broken, but posterodorsally it is clear that there is a moderately developed subarcuate fossa. Dorsolaterally, the petrosal bears a broad flat sutural connection to the overlapping squamosal. In ventral view the moderately pneumatized mastoid process of the petrosal appears to be at least partially exposed on the posteroventral corner of the cranium, posterior to the external auditory meatus and anterior to lateral extensions of the exoccipital. The strangest aspect of the ear region is the transversely broad and dorsoventrally deep gutter, extending dorsally (and possibly somewhat posteriorly) posterior to the cochlear housing, and suturing laterally with the mastoid process of the petrosal, that we interpret as being bordered posteriorly by a long lateral process of the exoccipital (paroccipital apophysis sensu [30]). Due to distortion, it isn’t clear what the orientation of these processes would have been in life. It is possible that this elongate gutter represents an exceptionally large jugular foramen and/or a fossa for muscle insertion. Between the paroccipital process and the base of the occipital condyle is the hypoglossal foramen.

The postglenoid process of the glenoid fossa curves deeply in a rostral direction and defines the caudal surface of that fossa, which is mediolaterally as wide as the mesiodistal length of M^1–2^. The rostrocaudal length of the glenoid fossa is approximately half its mediolateral width. The preglenoid process has a much lower relief than the arching postglenoid process. Medial to the postglenoid process is the foramen ovale, which is enclosed by the pterygoid bone proximate to the pterygoid process.

### Comparisons with crania of other hyaenodonts

Like *Brychotherium*, *Masrasector nananubis* has an elongate rostrum with retracted nasal bones that broaden laterally dorsal to the distal root of P^3^. *Brychotherium* also has a large facial process of the lacrimal that extends beyond the anterior margin of the orbit (dorsal to the mesial root of M^1^) to the mesial root of P^4^. The frontal bone of *M*. *nananubis* has more deeply excavated temporal lines than *Brychotherium*, but both taxa share low frontal peaks rather than pronounced postorbital processes. The low rostral profile and blunted postorbital processes are also shared with *Dissopsalis*. *Dissopsalis carnifex* is the only other teratodontine known from substantial cranial material. Like *M*. *nananubis*, the glenoid fossa of *Dissopsalis* is mediolaterally broad with a tall postglenoid process. The nuchal crest of *Dissopsalis carnifex* was reconstructed by Colbert [31], but appears to be based on *Hyaenodon* material and is not grounded in fossil material from *Dissopsalis*. Given the closer phylogenetic relationship between *Dissopsalis* and *Masrasector* the nuchal crest of *Dissopsalis* may not have resembled the low crest that trends toward the mastoid process possessed by *Hyaenodon* and reconstructed by Colbert [31].

The nuchal crest and basicranium are well preserved in *Apterodon macrognathus* and *Pterodon dasyuroides*. As illustrated by Solé et al. [30], both taxa share dorsoventrally tall, broad zygomatic arches, elongate neurocrania, indistinct postorbital processes, and fan-like nuchal crests with lateral margins that trend toward the foramen magnum, all characters used to define Apterodontinae and Hyainailourinae as part of a larger group called Hyainailouridae. *M*. *nananubis* shares all of these hyainailourid cranial features, though the morphology of the nuchal crest is not as mediolaterally narrow dorsal to the foramen magnum as it is in *P*. *dasyuroides* and *A*. *macrognathus*; instead, a portion of the lateral nuchal crest forms a ridge that connects to the mastoid process. This nuchal morphology is similar to the nuchal crest morphology of *Sinopa* and *Tritemnodon*, which both have fan-shaped nuchal processes that taper medially. The nuchal crest morphology of *Masrasector* contrasts with that of *Eurotherium* and *Hyaenodon*, which have nuchal crests that slope gradually ventrally, instead of being concave laterally, from the median apex of the crest, and form a broad nuchal shelf that trends toward the mastoid process. *Masrasector* further resembles *Kerberos* and *Pterodon* in having elongate and flattened processes of the exoccipital that form the posterior wall of a large fossa (an enlarged jugular foramen and/or a fossa for muscle insertion) posterior to the promontorium of the petrosal. This is unlike the condition in *Hyaenodon* and *Cynohyaenodon*, in which the posterior margin of the petrosal crowds the exoccipital, leaving little space between the structures.

*M*. *nananubis*, *Sinopa*, and *Tritemnodon* also have similar palatine morphology, with all having a posterior palatine torus formed at the posterior-most extent of the hard palate, and choanae that open rostral to the middle region of the cranium. *M*. *nananubis* differs from these Eocene North American taxa in having the hard palate extend slightly beyond the distal edge of M^3^. *Apterodon*, *Pterodon dasyuroides*, *Hyaenodon*, and *Eurotherium* all have caudally extensive narial tubes formed by partially sutured palatines that diverge anterior to the glenoid fossa.

The hard palate of *M*. *nananubis* is perforated by multiple foramina rather than two distinct greater palatine foramina. *Dissopsalis* also seems to have multiple small palatal foramina, as does *Apterodon macrognathus* and *Pterodon dasyuroides*. In contrast, *Hyaenodon* has two greater palatine foramina along the palatine-maxillary suture on the hard palate. The basicranial morphology of *Hyaenodon* also differs from *M*. *nananubis* by having evidence of an ectotympanic bulla [4] and a prong-like paroccipital process that is distinct from the mastoid process, while the latter structures are elongate in *M*. *nananubis*.

### Description of upper dentition

The crowns of the incisors of *Masrasector nananubis* are not preserved in any of the referred specimens, but the alveoli of the incisors are preserved in DPC 8275. The alveoli indicate there were three mesiodistally compressed incisors. I^1^ and I^2^ are comparable in diameter, and the diameter of I^3^ is about twice that of the more mesial incisors. It had an elliptical cross section at the alveolar margin like I^1^ and I^2^, but it is mesiodistally broader.

The upper canine is not preserved in any specimen, though the collapsed alveolus is preserved in DPC 13285. The canine is buccolingually compressed, and its mesiodistal length is subequal to the mesiodistal length of P^2^ (C = ~5.6 mm) and close to the same buccolingual width of P^2^ (C = ~2.7 mm).

The crown of P^1^ is also not preserved, but the alveoli indicate that the tooth is two-rooted. The distal root has a larger diameter, and both parallel the distal trend of the root of the canine. The mesial root of P^1^ is very close to the distal edge of the canine. The remaining upper dentition is preserved in multiple specimens. P^2^ has two roots and the crown forms an equilateral triangle in buccal view, with the preparacrista and postparacrista forming a ~70 degree angle at the apex of the tooth. The cristae are slightly buccolingually compressed and slope to the base of the tooth, which has thin lingual and buccal cingula. The buccolingual width of the tooth is about half its mesiodistal length. P^3^ is set at a slight angle (~23 degrees) to the mesiodistal axis of P^2^, following the lateral flare of the palate. Like P^2^, the crown of P^3^ forms an equilateral triangle in buccal view, and is buccolingually wide at its base. P^3^ differs from P^2^ by having a slight thickening of the lingual cingulum at the base of the paracone, although it is not a defined protocone. The distal point of P^3^ contacts P^4^ lingual to the termination of the P^4^ preparacrista. The paracone of P^4^ is similar to the paracones of P^2^–P^3^ in both buccolingual width and the symmetry of the pre- and postparacristae. The buccal cingulum on P^4^ is slightly thicker than the buccal cingulum of the other premolars. The postparacrista terminates in a very small notch, separating it from a metastyle that varies in mesiodistal length among specimens, from a minuscule cusp that is little more than a buccolingually compressed portion of the buccal cingulum and postprotocrista (DPC 13285) to a pronounced sectorial metastyle that is about one-third the length of the postparacrista (DPC 8276). The protocone is a bulbous, distinct cusp that is slightly less than half the height of the paracone. There is no lingual cingulum around the base of the protocone. The lingual face of the protocone bows lingually as it curves to the apex of the protocone. The trigon basin is narrow, but there is space between the protocone and paracone.

The metastyle of P^4^ contacts M^1^ lingual of the parastyle. P^4^ and M^1^ are mesiodistally subequal in length. The parastyle of M^1^ is a broad mesial shelf, rather than a buccolingually compressed cusp. The paracone and metacone on M^1^ are subequal in height and mesiodistal length, and both cusps are buccolingually compressed, with ovoid cross sections near the separation of the cusps. The paracone and metacone are fused at their bases, and separate halfway between the base and the apices of the cusps. The buccal and lingual faces of the paracone and metacone are distinguished by shallow grooves on their buccal and lingual surfaces, even where they are fused. The mesiodistally broad protocone projects to the same height as the paracone/metacone notch. The apex of the protocone is slightly mesial of the separation, aligned buccolingually with the apex of the paracone. A very small paraconule and metaconule are present, though these cusps appear worn away on the specimens. The preprotocrista connects with the parastyle but the postprotocrista does not connect with the metastyle. Instead, the postprotocrista ends at the lingual face of the metacone. The pre- and postprotocristae define the mesial and distal margins of a broad trigon basin that is as mesiodistally long as it is buccolingually wide. The metastyle is short, about the same mesiodistal length as the mesiodistal length of the metacone. The carnassial notch formed between the metastyle and postmetacrista is a shallow inflection rather than a deep incision. The metastyle is not buccolingually compressed into a narrow blade. Instead, the cusp slopes gently buccally from the metastylar blade to the buccal cingulum. The buccal cingulum does not form a deep ectoflexus on M^1^. Instead, the cingulum forms a wide shelf from the buccal edge of the metastyle, along the bases of the paracone and metacone, to the mesial point of the parastyle. The metastyle of M^1^ contacts the parastyle of M^2^ on the lingual face of the parastyle.

The parastyle of M^2^ is more prominent and cusp-like than the parastyle of M^1^, rising to the same height as the protocone and the divergence of the paracone and metacone. The parastyle connects to the buccal cingulum and preprotocrista. Along the preprotocrista is the low and rounded paraconule that forms a slight undulation in the preprotocrista before that crest rises to the apex of the protocone. The lingual surface of the protocone slopes buccally and the apex of the cusp defines the lingual extent of the trigon basin, which is mesiodistally as long as the trigon basin of M^1^, but buccolingually wider than that of M^1^. The apex of the protocone projects higher than the divergence of the paracone and metacone, but is lower than the paracone and metacone apices. The postprotocrista slopes distally to the base of the metacone, interrupted by the metaconule, which is shorter and smaller than the paraconule in its buccolingual and mesiodistal diameter. The paracone and metacone are buccolingually compressed, though the metacone is more compressed than the paracone. As on M^1^, shallow grooves define the intersection of the paracone and metacone on their fused buccal and lingual faces. The two cusps diverge halfway between their bases and apices. The paracone is slightly lower than the metacone and the paracone points mesially. The apex of the metacone projects perpendicular to the alveolar margin, and the premetacrista and postmetacrista have subequal mesiodistal lengths. The postmetacrista meets the metastylar blade in a deeper carnassial notch than that on M^1^. The metastyle is only slightly longer than the mesiodistal length of the metacone. As on M^1^, the metastyle has a low relief, and gently slopes from the metastylar blade to the buccal cingulum, forming a depression between the base of the metacone, the metastyle, and the buccal cingulum. The buccal cingulum is buccolingually broad and does not form a deep ectoflexus. Instead, the buccal cingulum rises to form a small, rounded mesostyle buccal to the bases of the metacone and paracone. Mesially the buccal cingulum curves slightly lingually before joining the parastyle, and distally the buccal cingulum forms a shallow, lingual curve to the base of the metastyle.

The metastyle of M^2^ contacts the parastyle of M^3^ just lingual to the parastylar cusp. The parastyle of M^3^ is a prominent cusp that rises to the same height as the protocone. The mesial edge of the parastyle is compressed into a thin, sharp crista that connects with the preprotocrista. A small paraconule and metaconule close the trigon basin mesially and distally. As on M^1–2^ the paraconule is the larger of the conules. The protocone projects lingually as far as the protocone of M^2^ and rises to the same height as the small M^3^ paracone. The trigon basin is mesiodistally narrower than the trigon basin of M^2^. The M^3^ paracone is of the same absolute height and mesiodistal length as the paracone of M^2^, but no metacone is present. Instead, the buccal cingulum connects with the postprotocrista along the distal margin of the paracone, rising slightly along the distal base of the paracone. M^3^ has two prominent roots and a thickened ridge on the lingual root dorsal to this “metacone” rise of the buccal cingulum.

### Comparisons with upper dentitions of other teratodontine hyaenodonts

*Masrasector nananubis* is similar to *M*. *ligabuei*, known from an isolated M^1^, and Holroyd [78] argued that the L-41 taxon and *M*. *ligabuei*, from the Taqah locality in Oman, belong to the same species. Holroyd’s hypothesis was bolstered by early proposals that L-41 correlated biostratigraphically with Taqah, the locality where *M*. *ligabuei* was found [24, 79]. However, there are morphological distinctions between the taxa that can be appreciated with the expanded sample of *M*. *nananubis* material collected over the last 20 years. In addition to morphological differences between the L-41 and Taqah *Masrasector* material, recent work has suggested that Taqah correlates more closely with the middle Rupelian, and Quarry G and Quarry V in the Fayum, both from higher in the Jebel Qatrani Formation and likely several million years younger than the latest Priabonian age that has been proposed for L-41 [34]. While temporal differences between the localities do not distinguish the species, it does increase the likelihood that there should be morphological differences between *Masrasector* at each locality. The mesiodistal length of the *M*. *ligabuei* M^1^ (~5.40 mm) falls between the average mesiodistal length of M^1^ (~4.72 mm) and M^2^ (~5.87 mm) in *M*. *nananubis*. The cusps are heavily worn in *M*. *ligabuei*, but the paracone and metacone are close to the same height as they are in *M*. *nananubis*. The protocone of *M*. *ligabuei* projects to the same height as the worn paracone and metacone, above the divergence between the cusps, unlike the protocone on M^1^ in *M*. *nananubis*, which only rises to the height of the paracone/metacone split. The protocone of *M*. *ligabuei* is broad, with the mesiodistal length of the protocone greater than the buccolingual width of the protocone. In *M*. *nananubis*, the mesiodistal length and buccolingual width of the M^1^ protocone are subequal.

Solé et al. [25] referred three isolated teeth from Bir el-Ater in Algeria to *Masrasector*, a referral we support based on the dentition of *M*. *nananubis*. UON 84–360 is an upper molar that Solé et al. [25] identified as either M^1^ or M^2^ and is similar in size to the molars of *M*. *nananubis*. The buccolingual width of UON 84–360 falls within the standard deviation for both M^1^ and M^2^ of *M*. *nananubis*, making it difficult to assign the tooth based on size, but based on the relative expression of the metacone, which has only a slightly wider base than that paracone, we tentatively identify this tooth as an M^1^. Unfortunately, both the parastyle and metastyle are not preserved in the Bir el-Ater *Masrasector* M^1^ and the cusps are heavily worn, making precise comparisons to *M*. *nananubis* difficult, but the protocone of *M*. *nananubis* seems to be more mesially oriented than the protocone of the Bir el Ater *Masrasector*. The protocone of the Bir el-Ater *Masrasector* also proportionally projects farther buccolingually than the M^1^ protocone of *M*. *nananubis*. Solé et al. [25] also referred an isolated M^3^ from Bir el-Ater (UON 84–361) to *Masrasector*, an assignment we can confirm based on *M*. *nananubis*. The Bir el-Ater M^3^ is not complete but seems to have a mesiodistally broader trigon basin than *M*. *nananubis*.

*Brychotherium ephalmos* is a teratodontine from L-41 known from much of the upper dentition. Like *M*. *nananubis*, *Brychotherium* has divergent paracones and metacones, distinct paraconules and metaconules, and a pronounced buccal cingulum. However, *Brychotherium* is larger than *Masrasector nananubis* (*B*. *ephalmos* M_3_ mesiodistal length = 9.6 mm; *M*. *nananubis* = 6.2 mm) and has many features that indicate it was more specialized for carnivory than *M*. *nananubis*, including a pronounced sectorial metastyle on P^4^ rather than a low metastyle that is of the same thickness as the buccal cingulum (as it is in *M*. *nananubis*). The protocone of the P^4^ of *Brychotherium* is low and pointed rather than bulbous as it is in *M*. *nananubis*. M^1, 2^ in *Brychotherium* exhibit many features that exaggerate the effective shear of the molars, such as long metastyles that are subequal to the mesiodistal length of the paracone and metacone. The shorter metastyles of *M*. *nananubis* are only as mesiodistally long as the metacone. *Brychotherium* also has more buccolingually compressed paracones and metacones than *M*. *nananubis*, and the metacone is taller and mesiodistally longer than the paracone. *M*. *nananubis* has more elliptical paracones and metacones and the apices of the cusps are close to the same height.

*Teratodon* is a Miocene teratodontine with molars that are closer in size to those of *M*. *nananubis* than are those of *B*. *ephalmos*. Both *Teratodon* and *Masrasector* share short molar metastyles that are approximately the mesiodistal length of the metacone, and broad and basined talon basins. *Teratodon* differs from *M*. *nananubis* by having molars that are mesiodistally longer than their buccolingual width, a deep ectoflexus shaped by a subequal parastyle and metastyle, and massive and bulbous premolars that are buccolingually wider than they are mesiodistally long.

*Glibzegdouia* is a middle Eocene teratodontine that is larger than *M*. *nananubis*. The upper dentition of *Glibzegdouia* is only known from a broken M^2^, which has a deeper ectoflexus than that of *M*. *nananubis*, and a relatively longer metastyle and buccolingually wider parastyle than the shorter metastyle and narrower parastyle of *M*. *nananubis*. The paracone and metacone of *Glibzegdouia* are elliptical in cross-section, like those of *M*. *nananubis*, rather than buccolingually compressed and blade-like as the paracone and metacone are on the molars of *Brychotherium*.

*Furodon* is also a middle Eocene teratodontine that is larger than *M*. *nananubis*. The upper dentition of *Furodon* is known from an isolated M^1^ that has a mesiodistally elongate metastyle that is subequal to the mesiodistal length of the paracone and metacone bases. M^1^ on *M*. *nananubis* has a much shorter metastyle that connects to a broad, shelf-like buccal cingulum that is unlike the narrow buccal cingulum of *Furodon*. The M^1^ paracone and metacone are more buccolingually compressed in *Furodon* than they are in *M*. *nananubis*.

Compared to the upper dentitions of the Miocene *Anasinopa* and *Dissopsalis*, *M*. *nananubis* has M^1, 2^ paracones and metacones that are subequal in height. The Miocene teratodontines have metacones that are much taller and more buccolingually compressed than the metacones of *M*. *nananubis*. Though, as in *M*. *nananubis*, the M^1, 2^ paracones in *Anasinopa* and *Dissopsalis* are conical near their bases rather than compressed with elliptical cross-sections. The M^1, 2^ protocones of *Anasinopa* and *Dissopsalis* are shifted more mesially than they are in *M*. *nananubis*.

### Description of the dentary

The dentary is deep, ~2.5 times the height of the trigonid of M_3_. The mandibular symphysis was unfused and the rugosity for the symphysis reaches from the mesial edge of the canine to the mid-point of P_3_. Three mental foramina are preserved. The most anterior mental foramen is ventral to the distal root of P_1_, the second mental foramen is ventral to P_3_, and the posterior mental foramen is ventral to P_4_. The ventral margin of the mandibular corpus is convex under the premolars and molars, bowing gently from the canine to an inflection point ventral to the apex of the coronoid process. Ventral to the coronoid process, the mandibular corpus bears a concave curve that ends at the rounded angular process. The angular process is traced medially by the ventral margin of a shallow fossa for the insertion of the medial pterygoid muscle. The mandibular foramen is horizontally positioned ventral to the apex of the coronoid process and positioned midway between the mandibular condyle and angular process in the dorsoventral plane. The mandibular foramen is framed by the lingula, which traces a deep “V” around the foramen. The mandibular canal is ~2 mm in diameter. The mandibular condyle sits dorsal to the alveolar margin at M_3_ when the dentary is placed with its most ventral point on a horizontal plane; however, it is approximately in line with the overall curvature of the alveolar margin. The mandibular condyle is dorsoventrally narrow and mediolaterally wide; its width is ~1.5 times the mesiodistal length of M_3_. Dorsal to the mandibular condyle, the posterior margin of the coronoid process is convex and bows medially. The coronoid process is not preserved completely in any specimens, testament to the deep masseteric fossa and mediolaterally thin bone that forms the process, but it is possible to build a composite coronoid from dentary specimens. The posterior margin curves to the apex of the coronoid process. The coronoid process is ~1.25 times taller than the dorsoventrally widest point of the mandibular corpus. The anterior margin of the coronoid process gently curves in a medial direction and slopes steeply to the alveolar margin. The coronoid process meets the mandibular corpus at a ~106 degree angle. A lateral ridge arises from the anterior margin of the coronoid process, originating about halfway from the apex of the coronoid process and tracing onto the mandibular corpus, blending into the corpus ventral to the distal root of M_3_. The expansion of the anterior coronoid ridge forms a broad attachment surface for the anterior fibers of the temporalis muscle. The masseteric fossa is demarcated by the anterior coronoid ridge and by a weaker posterior coronoid ridge. The ventral margin of the masseteric fossa is not deeply defined, but grades subtly from the fossa to the ventral margin of the mandible.

### Comparisons with dentaries of other teratodontines

*Masrasector aegypticum* is known from a dentary fragment that preserves a portion of the coronoid process [23]. Like the anterior margin of the coronoid process of *M*. *nananubis*, the coronoid process of *M*. *aegypticum* rises steeply from a point just distal to M_3_, and the masseteric fossa is sharply defined by the anterior ridge but is less clearly demarcated along the ventral margin of the fossa. The dentary corpus of *M*. *aegypticum* is dorsoventrally shallower than that of *M*. *nananubis*.

The dentary corpus of *Masrasector nananubis* is almost horizontal compared to the dentary corpus of *Teratodon* (KNM Ru 14769) [8]. The larger *Teratodon* has a distinct dentary inflection ventral to P_4_. The trend of the dentary corpus of *M*. *nananubis* is closer to the gradual ventral inflection of the corpus of *Anasinopa* (BNHM M19081C). The dentary of *Anasinopa* primarily differs from the dentary of *M*. *nananubis* by having a lower coronoid process. The anterior margin of the coronoid process of *Anasinopa* arises immediately posterior to the talonid basin of M_3_ and slopes at an obtuse angle rather than a steep, almost perpendicular angle, as it does in *M*. *nananubis*. The depth of the dentary corpus ventral to the alveolar margin of M_3_, is about one-and-one-half times the height of the crown of M_3_ in *Anasinopa* and the depth of the corpus is relatively shallower still in *Dissopsalis*. This contrasts with the deep dentary corpus of *M*. *nananubis*, which is about two times deeper than the height of the crown of M_3_. Like *M*. *nananubis*, the masseteric fossa is relatively shallow in *Anasinopa*, and the ventral margin of the fossa is not demarcated by a ridge or furrow, but instead gently grades from the masseteric fossa to the ventral margin of the dentary. *Brychotherium ephalmos* is closer in size to *M*. *nananubis* than *Anasinopa* and *Dissopsalis*, but like the Miocene teratodontines, it has a relatively narrow dentary corpus that is only slightly deeper (dorsoventrally) than the trigonid height of M_3_. The dentary of *Masrasector nananubis* is similar to the dentary of *Furodon*, with both taxa sharing a bowed ventral margin of the dentary, a steeply sloped anterior margin of the coronoid process, and a slight inflection near the angular process.

### Description of lower dentition

The lower incisor crowns are unknown. The canine is a tall and prominent tooth, the tallest in the tooth row, and it projects over half the height of the coronoid process. The distally recurved tooth is compressed buccolingually, though it remains conical rather than blade-like and does not bear a distinct sectorial distal cristid. The base is mesiodistally longer than all other teeth except M_3_. The lingual face of the canine has a broad, flattened facet and the enamel forms a lingual peak that is traced by the alveolar margin (see DPC 9274).

P_1_ contacts the distal aspect of the canine, actually overlapping with the distal edge of its base. P_1_ has two roots. The anterior root is positioned ventral to the apex of the low protocone. The distal root is ventral to the small talonid. Both roots sweep distally to parallel the much larger root of the canine. The low crown of P_1_ is set buccal to the midline of the canine. P_1_ is the shortest of the lower teeth. From the apex of the protoconid, the postprotocristid gently slopes to an inflection that defines a very short talonid. P_1_ is about twice as long as it is buccolingually wide.

P_2_ is mesiodistally longer than P_1_. P_2_ contacts P_1_ and its preprotocristid rises steeply from the contact to the apex of the P_2_ protoconid. The buccolingually compressed postprotocristid slopes more gradually to the distal-most point of P_2_, giving the tooth an asymmetrical profile in buccal view. A thin buccal and lingual cingulid traces the base of P_2_.

The dP_3_ (preserved in DPC 10358) is mesiodistally elongate with a distinct, low paraconid cusp. The protoconid is much taller than the paraconid and is mesiodistally longer. The protoconid is equilateral in buccal view and buccolingually compressed into a thin, sectorial cusp. A prominent distal cusp contacts the postprotocristid, forming a deep sectorial notch; it is taller than the paraconid. A small talonid basin is formed on dP_3_; a lightly worn hypoconulid and entoconid rim the talonid, but both are much lower than the hypoconid. The entocristid closes the talonid lingually.

The dP_4_ (preserved in DPC 10358) is molariform with a large, well developed trigonid and basined talonid. The protoconid is the tallest of the trigonid cusps and the paraconid is slightly shorter. The preprotocristid and postparacristid meet at a deep carnassial notch. The buccal margins of the preprotocristid and postparacristid are heavily worn, evidence of carnassial shear with dP^3^. The metaconid is slightly shorter than the paraconid. The distal face of the protoconid slopes distally to the cristid obliqua, which is buccolingually compressed and rises distally to the peak of the hypoconid (the tallest cusp on the talonid). The hypoconulid is not well developed; instead the distal margin of the talonid curves lingually from the hypoconid to the entoconid. The entoconid is positioned on the distolingual corner of the talonid. The entocristid slopes to the base of the metaconid, closing the lingual margin of the talonid, though the entocristid is not as tall as the cristid obliqua.

In contrast to dP_3_, P_3_ has a prominent but bulbous protoconid that lacks the sectorial profile of the dP_3_ protoconid. Like dP_3_, P_3_ has a small paraconid, but the paraconid of P_3_ is blunt and relatively short compared to that on dP_3_. The outline of P_3_ comes closer to forming an equilateral triangle in lingual view than does the crown of P_2_. The preprotocristid and postprotocristid are buccolingually compressed. The postprotocristid meets a short, buccolingually compressed cristid obliqua that forms the buccal edge of the short talonid basin. Like P_2_, P_3_ is traced by a thin buccal and lingual cingulid.

P_4_ is the same height as P_3_, and is shorter than the molars. The paraconid is very small with a slight postparacristid, though the entire cusp is almost indistinguishable from the buccal and lingual cingulids that together rim the tooth. The protoconid is symmetrical in lingual view; the preprotocristid and postprotocristid slope to the apex of the protoconid at the same angle. The postprotocristid terminates at the cristid obliqua, which forms the buccal margin of the shallow talonid basin. The talonid of P_4_ is rimmed by a distinct hypoconid, hypoconulid, and entoconid; of these, the hypoconid is the tallest cusp and the entoconid the lowest. The entocristid is prominent and partially closes the talonid lingually. The entocristid blends into the lingual cingulid directly lingual to the notch formed by the postprotocristid and cristid obliqua.

M_1_ is a buccolingually broad tooth when compared to the molariform dP_4_. The difference in width is particularly marked at the trigonid of M_1_. On dP_4_, the postparacristid and preprotocristid nearly parallel the trend of the mandibular corpus. On M_1_ the postparacristid and preprotocristid form a ~45 degree angle relative to the mandibular corpus. On M_1_ the metaconid is more mesially positioned than on dP_4_, almost directly lingual to the protoconid, rather than slightly distal to the protoconid. The base of the metaconid of M_1_ abuts the base of the paraconid, which projects mesially. On its buccal face there is a small anterior keel that braces the buccal portion of the talonid of P_4_. The tallest cusp of the trigonid is the protoconid. The paraconid is shorter than the protoconid, and the metaconid is slightly shorter than the paraconid. The talonid basin is slightly less than half the entire mesiodistal length of the tooth. The cristid obliqua rises steeply from the base of the protoconid to the apex of the hypoconid. The hypoconid apex is close to the distobuccal corner of the talonid basin. The hypoconid slopes to the small hypoconulid at the distal-most point of the tooth. The entoconid is taller than the hypoconulid. The entocristid on M_1_ slopes more steeply to the base of the metaconid than the entocristid does on dP_4_.

M_2_ is mesiodistally longer than M_1_. Compared to M_1_, M_2_ is taller, the protoconid is more buccolingually compressed, the paraconid is proportionally shorter, and the metaconid is much lower than the paraconid. The hypoconid is the largest cusp of the talonid, and the hypoconulid of M_2_ is relatively large when compared with that of M_1_, and there is a distinct buccal inflection between the base of the hypoconid and the hypoconulid. The entoconid is also a better defined cusp on M_2_ than on M_1_. The entocristid slopes from the apex of the entoconid to the base of the trigonid at a more gradual angle than that on M_1_, and a slight lingual space is left open between the trigonid and entocristid. On the buccal surface of M_2_, a thin buccal cingulid traces from the anterior keel bracing the buccal portion of the talonid of M_1_ to the distal edge of the base of the protoconid.

M_3_ is the tallest postcanine tooth in the lower tooth row, and the longest tooth mesiodistally. When compared to M_2_, the paraconid of M_3_ is closer in height to the protoconid, the metaconid is lower relative to the apex of the paraconid, the trigonid is relatively tall, the talonid relatively narrow, and the hypoconulid relatively small. The hypoconulid is the distal-most point of the tooth and occupies a more centralized position along the distal talonid margin. The entoconid is not prominent. Instead, the entocristid slopes from the hypoconulid to the base of the trigonid, leaving a slight lingual opening along the lingual portion of the talonid. As on M_2_, a thin buccal cingulid traces from the buccal face of the paraconid to the base of protoconid.

### Comparisons with lower dentitions of other teratodontines

The lone premolar known from *Masrasector aegypticum* is a heavily worn P_3_. Compared to P_3_ in *Masrasector nananubis*, that of *M*. *aegypticum* is buccolingually wide and more than half its mesiodistal length, whereas that of *M*. *nananubis* is about half as wide as it is long. The molars of *M*. *aegypticum* are also broad and robust compared to those of *M*. *nananubis*. The trigonids of *M*. *aegypticum* are worn, but appear to be closer to the maximum height of the talonids. The talonids are traced by buccal cingulids that run from the paraconid all the way to the distal talonid, unlike the buccal cingulids of *M*. *nananubis* that terminate at the base of the protoconid. The talonid cusps are buccolingually broader in *M*. *aegypticum*, especially the entoconid. The hypoconulid is very small, squaring the distal margin of the talonid in occlusal view. Overall, *M*. *aegypticum* is larger than *M*. *nananubis*.

Solé et al. [25] identified an isolated P_3_ referred to *Masrasector* (UON 84–397) from Bir el-Ater. Like that of *M*. *aegypticum*, the Bir el-Ater P_3_ is heavily worn, but enough of the base is preserved to observe the tooth is similar in mesiodistal length to the P_3_ of *M*. *nananubis*. The Bir el-Ater P_3_ is buccolingually wider than the P_3_ of *M*. *nananubis* and, like *M*. *aegypticum*, the buccolingual width of the Bir el-Ater P_3_ is over half its mesiodistal length, indicating a more robust premolar than is found in *M*. *nananubis*.

*Masrasector ligabuei* is known from a right M_3_ that is between the ranges of M_2_ and M_3_ in *M*. *nananubis*. Holroyd [78] hypothesized that the specimen from Oman was incorrectly identified by Crochet et al. [24]. Based on comparisons to an even more expansive collection of *M*. *nananubis* dentaries than Holroyd [78] had to work with, we support Crochet’s original interpretation of the *M*. *ligabuei* lower molar as being an M_3_, because the cristid obliqua parallels the mesiodistal axis of the tooth, rather than angling lingually from the apex of the hypoconulid, and also because the hypoconulid is well defined and occupies the distal-most point on the ovoid talonid basin. The M_3_ of *M*. *ligabuei* differs from those *M*. *nananubis* by having a trigonid that occupies more of the mesiodistal length of the tooth (~60% as opposed to ~50%). The anterior keel of M_3_ is better developed in *M*. *ligabuei* and the hypoconid is buccolingually compressed, while M_3_ in *M*. *nananubis* has a less projecting anterior keel on the paraconid and the hypoconid is more lingually expansive, crowding the talonid basin. All three species of *Masrasector* share the prominent, basined talonid basin, a connate metaconid that is lower than the paraconid, entocristids that connect an indistinct entoconid to the base of the metaconid, and relatively low trigonids compared to the talonids.

*Brychotherium ephalmos* shares with *Masrasector nananubis* the presence of small, connate molar metaconids that are smaller than the paraconids and progressively smaller on each successive molar. Like *M*. *nananubis*, the protoconid of the P_4_ of *Brychotherium* forms roughly an equilateral triangle in lingual view, but the buccal cingulid of *M*. *nananubis* is more extensive and the talonid basin is buccolingually broader than the talonid of *Brychotherium*, which also has a tall, buccolingually compressed hypoconid. On the M_3_ of *Brychotherium*, there is a greater disparity in height between the trigonid and talonid than there is in *M*. *nananubis*, and the postprotocristid and preprotocristid are closer to parallel to the long axis of the mandibular corpus.

*Glibzegdouia tabelbalaensis* shares many features with *Masrasector*, including the retention of a connate metaconid and large, basined talonid. The M_1_ metaconid of *Glibzegdouia* is taller than the paraconid, while it is shorter than the paraconid on all molars in *M*. *nananubis*. *Glibzegdouia* has a well-defined entoconid that slopes to a distinct notch at the base of the metaconid; the entoconid in *M*. *nananubis* does not have a distinct peak, and the entocristid blends into the trigonid without a clear notch in the entocristid. Like *M*. *nananubis*, *Glibzegdouia* has a short anterior keel, but the keel only runs along the base of the paraconid. In *M*. *nananubis*, the anterior keel is part of the buccal cingulid and runs to the base of the protoconid.

The Miocene teratodontines *Anasinopa*, *Dissopsalis*, and *Teratodon* share with *Masrasector nananubis* the presence of a low metaconid on at least M_1_ and M_2_. Of the Miocene teratodontines, *Anasinopa* is most similar to *M*. *nananubis* in having premolars that are buccolingually half their mesiodistal length and a metaconid on all molars. The morphology of the talonid basins of *Anasinopa* differs from that of *M*. *nananubis* by having almost no distinction between the hypoconulid and entoconid and lacking an entocristid on M_3_, leaving the talonid open lingually. *Teratodon* has lower molars that are also similar to those of *Masrasector*, but its premolars are massive, with buccolingual widths that are more than half their mesiodistal lengths. The molars of *Teratodon* are mesiodistally shorter than the premolars, and they share many features with *M*. *nananubis* such as a small anterior keel, a lingually closed talonid on M_3_, a paraconid that is only slightly lower than the protoconid, and a prominent metaconid on M_3_. M_1_ and M_2_ in *Dissopsalis* are similar to the same teeth in *M*. *nananubis*, with the metaconid retained and the talonid basined. The talonid cusps are not as well-defined in *Dissopsalis* as they are in *M*. *nananubis*, and the angles formed by the preprotocristid and postparacristid relative to the dentary corpus is closer to the angle formed between these structures on dP_4_ in *M*. *nananubis*. The M_3_ of *Dissopsalis* lacks a metaconid and the talonid is reduced to a small hypoconid, and each more mesial molar is quite different. In general, the differences between subsequent molars in *M*. *nananubis* are not as dramatic as those between subsequent molars of *Dissopsalis* and, to a lesser degree, *Anasinopa*.

### Body mass estimates

Table 3 contains all estimated body mass values based on dental and cranial length measurements. The average body mass of *Masrasector nananubis* based on all equations applied to each element is ~1.05 kg with an extensive range in body mass estimates from 0.43 kg to 2.04 kg When average body mass is estimated, excluding body mass estimates based on M_1_, which was not the largest carnassial in the tooth row as it is in carnivorans, average body mass rises to ~1.16 kg. The estimated body mass for *M*. *nananubis* is within the range of modern carnivorans like *Mephitis mephitis* (striped skunk; 0.7–6.3 kg; [44]) and *Genetta genetta* (small-spotted genet; 1.0–3.0 kg; [66]).

**Table 3:** Body mass estimates (kg) for *Masrasector nananubis*.

Equation and element	Raw length (mm)	Body Mass estimate (kg)
Morlo M_1_	4.45	0.43
VV M_1_	4.45	0.72
Morlo M_2_	5.28	0.78
VV M_2_	5.28	0.88
Morlo M_3_	6.20	1.36
VV M_3_	6.20	1.07
Morlo M avg.	5.31	0.79
VV M avg.	5.31	0.89
VV Cranium	81.25	2.04
VV Occ. to Orb.	55.75	1.50

**Morlo**, calculation based on methods used by Morlo [15]; **VV**, calculation based on methods used by Van Valkenburgh [44]; **Occ.**, occipital condyle; **Orb.**, anterior orbital margin.

### Description of distal humeri from L-41

**Fig 13:**
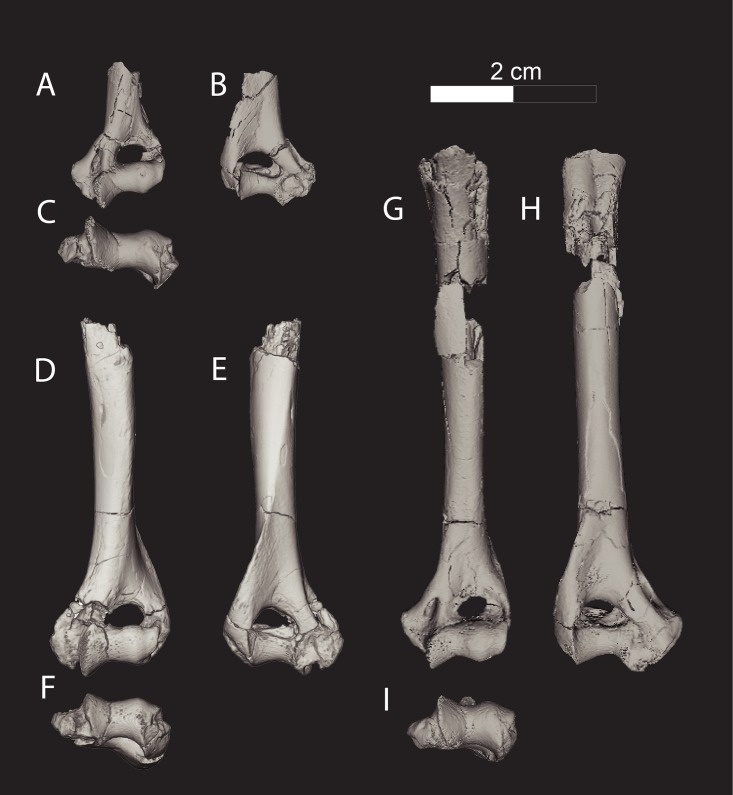
Left humerus specimens referred to *Masrasector*. DPC 10831 in (**A)** anterior view, (**B)**, posterior view, (**C)**, distal view, medial to left. DPC 15436 in (**D)** anterior view, (**E)** posterior view, (**F)** distal view, medial to left. DPC 11670 in (**G)** anterior view, (**H)** posterior view, (**I)** distal view, medial to left. Digital models were generated in Avizo and are available on Morphosource.

Three small distal humeri have been found at L-41 that are similar in overall morphology to small hyaenodont humeri known from other continents (Fig 13; Table 4). Based on their comparable size and morphology, the simplest explanation is that they belong to the same taxon. The only other mammals known from L-41 that are of the approximate size class of the humeri are macroscelideans, hyracoids, and primates, but no living or extinct members of these clades are known to exhibit morphology similar to that seen in the distal humeri described here. The mediolateral width of the distal articulation of the humeri described here ranges between ~13.23 mm and ~14.53 mm. Based on comparisons of the relative sizes of skulls and the distal humeri of extant carnivorans (particularly *Genetta genetta*, *Mephitis mephitis*, and *Fossa fossana*), as well as the abundance of *Masrasector nananubis* at L-41, we refer these humeri to that species.

**Table 4:** Descriptive humeral measurements for L-41 cf. *Masrasector*.

Measurement (mm)	DPC 10831	DPC 11670	DPC 15436
Specimen length	17.6	62.8	42.9
Maximum ML width	14.5	13.2	14.2
ML Articular width	8.4	9.8	9.8
ML med. epicondyle width	3.8	3.9	3.1
PD med. epicondyle height	4.9	5.7	5.1
ML Max. brachial flange width	2.8	2.5	3.0
PD Capitulum diameter	4.0	5.2	5.1

**ML,** Medio-lateral; **med.**, medial; **PD**, Proximo-distal

The proximal part of the humerus is not known, though DPC 11670 preserves most of the shaft. The humerus is gracile, the proximodistal length about five times the mediolateral width of the distal articulation. The shaft has a circular cross-section near the middle of the shaft that then becomes triangular near the origin of the supinator crest. The supinator crest arises on the dorsal aspect of the humerus at about 1.25 mediolateral distal humerus widths from the distal point of the humerus (in absolute terms ~22 mm from the distal humerus). The supinator crest wraps from the posterior face of the shaft onto the lateral epicondyle as it traces the humerus distally, forming a shallow fossa between its lateral edge and the widening shaft of the humerus, which is the site of origin for the brachioradialis muscle and the carpal and digital extensor musculature. The average maximum mediolateral width of the supinator crest is ~2.55 mm. The supinator crest narrows as the distal humerus widens to accommodate the distal articulation, and the supinator crest merges into the mediolaterally narrow and anteriorly curved lateral epicondyle. The lateral epicondyle laterally braces the rounded capitulum. The main body of the capitulum is almost spherical, with its mediolateral, proximodistal, and anteroposterior diameter being subequal. Between the rounded, proximodistally tallest portion of the capitulum and the lateral epicondyle, the articular surface has a proximodistally shorter articular surface. Medial to the rounded capitulum, the distal articulation narrows around the trochlea for the ulna, and the proximal margin of the trochlea slopes medially and distally. The distal portion of the trochlea projects distally beyond the distal-most point of the capitulum. The anteroposterior depth of the trochlea is subequal to the greatest anteroposterior depth of the capitulum.

The medial epicondyle is proximodistally elongate (~4.86 mm to ~5.68 mm), subequal in proximodistal length to the mediolateral width of the trochlea. In distal view, the medial epicondyle is aligned with the long axis of the distal articulation. In anterior view, the proximal and distal margins of the medial epicondyle form approximately a 90-degree angle with respect to each other, rather than being rounded and bulbous. The medial face of the medial epicondyle trends from its medial-most point in the same mediolateral plane as the proximal-most point of the capitulum toward the distal trochlea at approximately a 45-degree angle. The medial epicondyle is perforated by an elliptical entepicondylar foramen that has its long axis running parallel to the proximomedial margin of the medial epicondyle. Proximal to the capitulum is an elliptical supracondylar foramen. The relative width of the foramen varies between the specimens, with DPC 11670 retaining thin sheets of bone that encircle a relatively restricted foramen, while the supracondylar foramen in DPC 10831 is mediolaterally wider and stretches between the medial and lateral struts of bone that connect the shaft of the humerus to the distal, condylar portion of the humerus. In distal view, the articular surface forms a broad surface between the medial and lateral epicondyles that is about the same width as the deep olecranon fossa. In posterior view, a variably developed dorsoepitrochlear fossa is preserved for the attachment of the ulnar collateral ligaments and the joint capsule of the elbow is framed between the posterior, medial margin of the trochlea and the medial epicondyle.

There are subtle differences between the three humeri, particularly between DPC 10831 and the other two humeri in the sample. The capitulum of DPC 10831 is slightly more ellipsoidal, with a wider mediolateral axis than proximodistal axis. The dorsoepitrochlear fossa is also deeper and better defined on DPC 10831 than on either of the other specimens. The medial epicondyle of DPC 11670 has a slight posterior deflection in distal view compared to the other two humeri though the mediolateral width of all three medial epicondyles is constrained to a narrow (<1 mm) range (~3.14 mm to ~3.91 mm).

### Comparisons to other hyaenodont humeri

**Fig 14:**
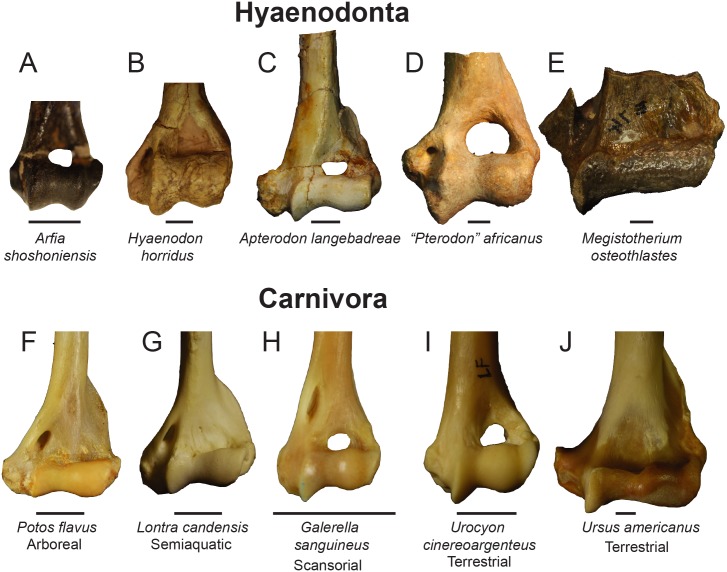
Comparative sample of distal humerus specimens of Hyaenodonta and Carnivora. Comparative sample of distal humerus specimens of Hyaenodonta (**A–E**) and Carnivora (**F–J**) in anterior view (all left specimens except **E,** which has been flipped). (**A**) *Arfia shoshoniensis*, AMNH 15515, (**B**) *Hyaenodon horridus*, AMNH 1381, (**C)** *Apterodon langebadreae*, BNHM M85318, (**D)** “*Pterodon” africanus*, BNHM M8886, (**E)** *Megistotherium osteothlastes*, BNHM UB20577, (**F)** *Potos flavus* (kinkajou), Arboreal, AMNH 46513, (**G)** *Lontra canadensis*, northern river otter, Semiaquatic, MCZ 62635, (**H)** *Galerella sanguineus*, slender mongoose, Scansorial, AMNH 187750, (**I)** *Urocyon cinereoargenteus*, gray fox, Terrestrial, MCZ 64169, (**J)** *Ursus americanus*, black bear, Terrestrial, MCZ 59938. Specimens not to scale. Black horizontal lines = 10 mm.

Fig 14 shows a sample of hyaenodont distal humeri. Only four Afro-Arabian taxa have humeri referred to them: *Apterodon langebadreae*, from the late Eocene or early Oligocene of Dur At-Talah, Libya [11]; *Apterodon macrognathus* from the early Oligocene of the Fayum, Egypt [80, 81]; “*Pterodon*”*africanus* from the early Oligocene of the Fayum, Egypt [80, 81]; and *Megistotherium osteothlastes* from the middle Miocene of Gebel Zaltan, Libya [5]. Ginsburg [82] hypothesized that the distal humerus Savage [5] referred to *Megistotherium* may actually be the distal humerus of a large amphicyonid, noting the articulation of the specimen is ursid-like. We agree it is ursid-like, but the postcrania of large hyainailourines are poorly understood and all are based on isolated referred material (like the L-41 *Masrasector* humeri) and we will retain Savage’s [5] original assignment until better associated hyainailourine material is discovered. The humeri referred to each of the hyaenodont taxa from Afro-Arabia are much larger than the humeri from L-41 referred to *Masrasector*.

The smallest referred Afro-Arabian hyaenodont humerus belongs to *Apterodon langebadreae*, which is ~42 mm wide at its distal end, and about three times the maximum mediolateral width of the L-41 *Masrasector* humeri. The distal humerus of *Apterodon macrognathus* is similar to that of *A*. *langebadreae*. Compared to the L-41 humeri, the distal humerus of *A*. *langebadreae* has a much larger, more rounded medial epicondyle that projects relatively far medially, and it has no entepicondylar foramen; the medial epicondyles are also all aligned with the mediolateral axis of the distal articulation. The capitulum of *A*. *langebadreae* is mediolaterally more elongate and ellipsoid than those of the L-41 humeri, and the shaft of *A*. *langebadreae* is triangular in cross section and bows anteriorly more than the humeri from L-41. The trochleae of the L-41 humeri and *A*. *langebadreae* project beyond the capitulum and slope slightly medially and distally along the proximal margin of the trochlea.

The bear-like distal humerus of *Megistotherium osteothlastes* differs from the L-41 humeri by having a columnar capitulum with little demarcation of the lateral trochlear margin relative to the articular surface of the capitulum. The deep olecranon fossa of *Megistotherium* is unperforated, and the base of the medial epicondyle indicates that it would have been posteriorly directed, rather than medially directed as in the L-41 specimens. Overall, the morphology of the distal humerus of *Megistotherium* resembles those of some other large mammals like ursids and even rhinocerotids and hippopotamids. The Afro-Arabian humeral specimens that the L-41 specimens most closely resemble are referred to “*Pterodon*” *africanus*. Though much larger (maximum mediolateral width ~60 mm) the distal humerus of “*P*.” *africanus* shares with the L-41 specimens a spherical capitulum, distally projecting trochlea, and relatively small medial epicondyle that only expands a short distance medial to the entepicondylar foramen. Besides differing in size, the humeri of “*P*.” *africanus* differ from the L-41 humeri in having a rounded supracondylar foramen, and a very narrow supinator crest that does not flare broadly proximal to the lateral epicondyle.

*Kyawdawia*, a late middle Eocene “indohyaenodontine” from Myanmar, is represented by a humerus [28]. Compared to the L-41 humeri, the distal humerus of *Kyawdawia* is larger and more robust, with a relatively large medial epicondyle and a shallow olecranon fossa rather than a supracondylar foramen. The supinator crest of *Kyawdawia* is not preserved, but Egi et al. [28] suggested that this structure was wide and laterally flaring. If correct, this morphology would contrast with the relatively narrow supinator crest of the L-41 humeri. The distal extent of the deltopectoral crest of *Kyawdawia* overlaps extensively with the origins of the supinator crest. The shafts of the L-41 specimens are circular in cross section, rather than triangular, and the deltopectoral crest is limited to the proximal portions of the shaft. Overall, the distal humerus of *Kyawdawia* appears to share more morphological similarities with *Apterodon* than with the L-41 specimens.

The North American hyaenodont record contains several specimens with associated craniodental and postcranial material [4, 6, 13] and many distal humeral elements that have been referred to hyaenodonts [20, 83]. An isolated humerus from the early Eocene of North America referred to *Arfia shoshoniensis* is similar in size to those from L-41 and it bears a deep inflection between the capitulum and trochlea and a reduced medial epicondyle that is aligned with the mediolateral axis of the distal humerus. The humeral shaft is also gracile, with the deltopectoral crest forming only a slight anterior eminence on the proximal shaft. North American *Hyaenodon*, including *Hyaenodon horridus* from the early Oligocene, is known from multiple, associated humeri and, though larger than the L-41 specimens, it shares a spherical capitulum and proximodistally tall medial epicondyle. Unlike the proximal trochlear margin of the L-41 specimens, the anterior and proximal margins of the trochleae of *Arfia* and *Hyaenodon* trend medially and proximally, sloping slightly upward from the capitulum to the medial edge of the trochlea.

### Phylogenetic results

#### Summary of the position of *Masrasector nananubis* across phylogenetic methods

Across all phylogenetic methods, *Masrasector nananubis* is resolved as closely related to the other known species of *Masrasector*: *M*. *ligabuei* and *M*. *aegypticum*. Across all phylogenetic methods, all *Masrasector* species are nested within Teratodontinae. Using Bayesian inference, *Masrasector* is a clade, though there is variability in the relationships among the included species. Using maximum parsimony analysis, *Masrasector* is not consistently resolved as a clade, instead forming a polytomy in the strict consensus tree with the clade that contains the teratodontines *Furodon*, *Anasinopa*, *Brychotherium*, and *Dissopsalis*.

#### Standard Bayesian phylogenetic inference

**Fig 15:**
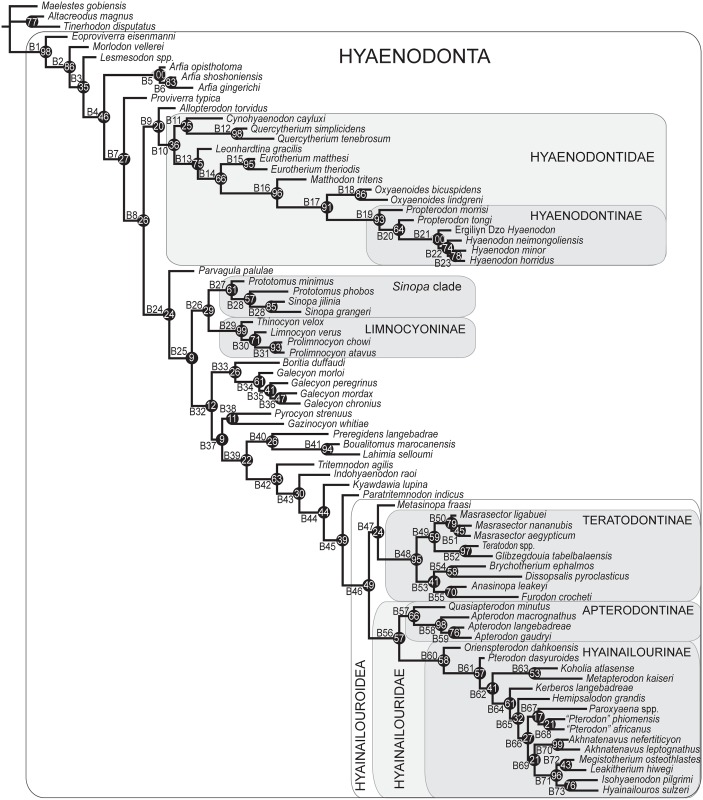
Standard Bayesian consensus tree. “Allcompat” consensus (majority rule plus compatible groups) tree. B# corresponds to the node to the right of the label and are used in the results and discussion sections. Posterior probabilities (PP) are placed in the center of the black circle over each node.

The “allcompat” (majority-rule plus compatible groups) topology recovered by the standard Bayesian analysis is shown in Fig 15 with the posterior probability (PP) of each node enclosed in the black circle over each node. The original tree file output by MrBayes is S3 Dataset. *Masrasector* is monophyletic (B50, PP = 0.79), and *Masrasector nananubis* is placed as the sister taxon of *Masrasector aegypticum* (B51, PP = 0.45). *Masrasector* is the sister clade to *Teratodon* + *Glibzegdouia* (B50; PP = 0.59) and the sister clade to (*Masrasector* (*Teratodon* + *Glibzegdouia*)) contains the remaining teratodontines *Dissopsalis*, *Brychotherium*, *Anasinopa*, and *Furodon*. Overall Teratodontinae has strong support as a clade (B48, PP = 0.95). There is very weak support for *Metasinopa* as the sister taxon of Teratodontinae (B47, PP = 0.24).

Hyainailourinae is moderately supported (B60; PP = 0.58) as is Apterodontinae (B57; PP = 0.66) and these major Afro-Arabian clades are united as sister clades within Hyainailouridae with moderate support (B56; PP = 0.57). Teratodontinae and Hyainailouridae are sister clades in the clade Hyainailouroidea (B46; PP = 0.49), a clade that unites almost all Afro-Arabian hyaenodonts. The only remaining Afro-Arabian hyaenodonts, *Lahimia* and *Boualitomus*, are resolved as sister taxa (B41; PP = 0.94) with European *Preregidens* weakly supported as their sister taxon (B40; PP = 0.26). Intervening between the *Lahimia* + *Boualitomus* clade and Hyainailouroidea is a succession of North American (*Tritemnodon*; B63; PP = 0.63) and south Asian taxa (*Indohyaenodon*, *Kyawdawia*, and *Paratritemnodon*), with *Paratritemnodon* resolved with weak support as the most proximate sister taxon to Hyainailouroidea (B45; PP = 0.39).

*Eoproviverra* is recovered as the sister taxon to all other hyaenodonts (B1; PP = 0.98) and *Morlodon* (B2; PP = 0.86), *Lesmesodon* (B3; PP = 0.35), *Arfia* (B4; PP = 0.46), and *Proviverra* (B7; PP = 0.27) are successive, more deeply nested taxa along a hyaenodont stem that unites the major clade that includes Hyaenodontidae (B9; PP = 0.20) and the clade that includes Hyainailouroidea (B24; PP = 0.24). Hyaenodontidae (the clade that includes the common ancestor of *Cynohyaenodon* and *Hyaenodon*) is weakly supported (B10; PP = 0.36). Nested within Hyaenodontidae is Hyaenodontinae, a clade with robust support (B19; PP = 0.93). One of the earliest diverging branches in clade B24 supports a clade (B26; PP = 0.29) that contains the robustly supported Limnocyoninae (B29; PP = 0.99) and moderately supported *Sinopa* clade (B27; PP = 0.61). The next most deeply nested clade (B33; PP = 0.26) recovers *Boritia* as the sister taxon of *Galecyon* (B34; PP = 0.61). More deeply nested than the *Galecyon* + *Boritia* clade is *Pyrocyon + Gazinocyon* (B38; PP = 0.11), a clade with very weak support that in turn is very weakly supported (B37; PP = 0.09) as the sister clade of clade B39, the clade that contains all Afro-Arabian hyaenodonts included in the analysis except for *Tinerhodon* which was recovered outside of Hyaenodonta as the sister taxon of *Altacreodus* (PP = 0.77).

#### Tip-dating Bayesian inference

**Fig 16:**
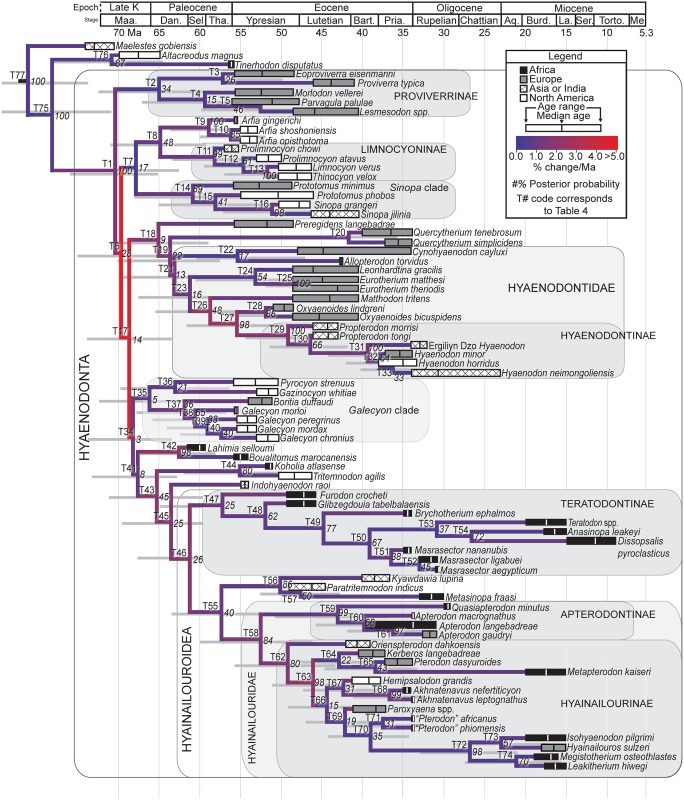
Tip-dating consensus tree. “Allcompat” (majority rule plus compatible groups) consensus derived from the post-burnin sample of trees recovered by tip-dating Bayesian analysis. T# corresponds to S3 Table and the results and discussion section. Posterior probability (PP) shown in italics to the right or below relevant node. Divergence dates represent median divergence date for clades and taxa. The vertical line over each geological age range (see S2 Table for sources of age information) is the median estimated age for the taxon based on the tip-dating analysis. Branch colors correspond to legend for % change/Ma along branches. More rapidly changing branches include more red and more slowly changing branches include more blue. Most rapidly evolving lineages are near the base of Hyaenodonta. Continental area of origin for each taxon is shown by the shading the proposed age range: **black**, Afro-Arabia; **grey**, Europe; **checked**, Asia or India; **white**, North America. **Aq.**, Aquitanian; **Bart.**, Bartonian; **Burd.**, Burdigalian; **Dan.**, Danian; **La.**, Langhian; **Late K**, Late Cretaceous; **Maa.**, Maastrichtian; **Me.**, Messinian; **Pria.**, Priabonian; **Sel.**, Selanian; **Ser.**, Serravallian; **Tha.**, Thanetian; **Torto.**, Tortonian.

The “allcompat” topology recovered through tip-dating Bayesian inference Fig 16 and statistics related to the topology, including rates of evolution and divergence estimates, are presented in S3 Table and the original tree file with additional statistical information is S5 Dataset. Note that in the raw tree S5 Dataset the ages are offset by 11.44 Ma. Overall median clock rate for the analysis is 0.005995. The overall median clock rate was multiplied by the relative median rate of each branch then that value was multiplied by 100 to calculate % change/Ma for each branch in the analysis. As in the standard Bayesian analysis *Masrasector* is monophyletic, though the clade is more weakly supported (T51, PP = 0.38). In the tip-dating topology *M*. *nananubis* is the sister taxon of the two Oligocene *Masrasector* species (T52, PP = 0.45) rather than the sister taxon of *M*. *aegypticum*. The branch leading to *Masrasector* is reconstructed as having a relatively slow rate of evolution (T51; 0.90% change/Ma).

The tip-dating analysis recovers the same taxa within Teratodontinae that were recovered by standard Bayesian analysis (T47; PP = 0.25; 1.15% change/Ma), but the relationships within the clade differ and the tip-dating topology better conforms to the order of appearance of teratodontines in the fossil record of Afro-Arabia. Early or middle Eocene *Furodon* is the sister taxon of all other teratodontines and contemporaneous *Glibzegdouia* is moderately supported as the sister group of other teratodontines (T48; PP = 0.62). In the tip-dating analysis, *Brychotherium* is the sister taxon (T49; PP = 0.77) of the clade that includes *Masrasector* and the early Miocene teratodontines *Teratodon*, *Anasinopa*, and *Dissopsalis* (T50; PP = 0.67).

Overall, the topology recovered using tip-dating analysis is similar to the standard Bayesian topology with major clades like Hyainailouridae (T62; PP = 0.80) and Hyaenodontidae (T21; PP = 0.13) recovered with most of the same taxa. Where the topologies differ, the tip-dating analysis tends to reduce the length of ghost lineages implied by the standard Bayesian topology. For example, early Eocene *Koholia* was deeply nested within Hyainailouroidea in the standard Bayesian topology, but the tip-dating analysis resolved *Koholia* as more likely to have diverged earlier in the evolution of hyaenodonts. In the tip-dating analysis, *Paratritemnodon* and *Kyawdawia* are sister taxa (T56; PP = 0.86) and the clade that these south Asian OTUs form is the sister clade to Hyainailouridae (T58; PP = 0.84). *Indohyaenodon* is weakly supported (T45; PP = 0.25) as the sister taxon to Hyainailouroidea (T46; PP = 0.26). *Lahimia* and *Boualitomus* are again recovered outside of Hyainailouroidea, though they do not form a larger clade with *Preregidens*. Instead, *Lahimia + Boualitomus* (T42; PP = 0.98) forms a very weakly supported clade (T41; PP = 0.08) with the large clade that unites *Koholia*, *Tritemnodon*, *Indohyaenodon* and Hyainailouroidea (T43; PP = 0.45).

The tip-dating topology resolves a series of short, rapidly evolving branches near the origin of Hyaenodonta. The median age for the origin of Hyaenodonta is 70.5 Ma, and the 95% HPD (highest posterior density) for the age of the origin of the group ranges between 76.1 Ma (Late Cretaceous) and 65.4 Ma (earliest Paleocene). The earliest diverging clade in Hyaenodonta (T2; PP = 0.34) unites many of the European hyaenodonts recovered by the standard Bayesian analysis as early diverging successive sister taxa to the rest of Hyaenodonta. Clade T6 is supported by the most rapidly evolving branch in the analysis (8.91% change/Ma). In the tip-dating topology *Arfia* (T9; PP = 1.0), the *Sinopa* clade (T14; PP = 0.89), and Limnocyoninae (T11; PP = 0.99) form a weakly supported clade (T7; PP = 0.17) that is the sister clade to the larger hyaenodont clade that includes Hyaenodontidae and Hyainailouroidea (T17; PP = 0.14). The branch that supports Hyaenodontinae and Hyainailouroidea (T17) is the second-most rapidly evolving lineage in the analysis (4.5% change/Ma). Like in the standard Bayesian analysis, Hyaenodontidae is weakly supported (T21; PP = 0.13), but Hyaenodontinae, nested within Hyaenodontidae, has robust support (T29; PP = 1.0).

#### Maximum parsimony

**Fig 17:**
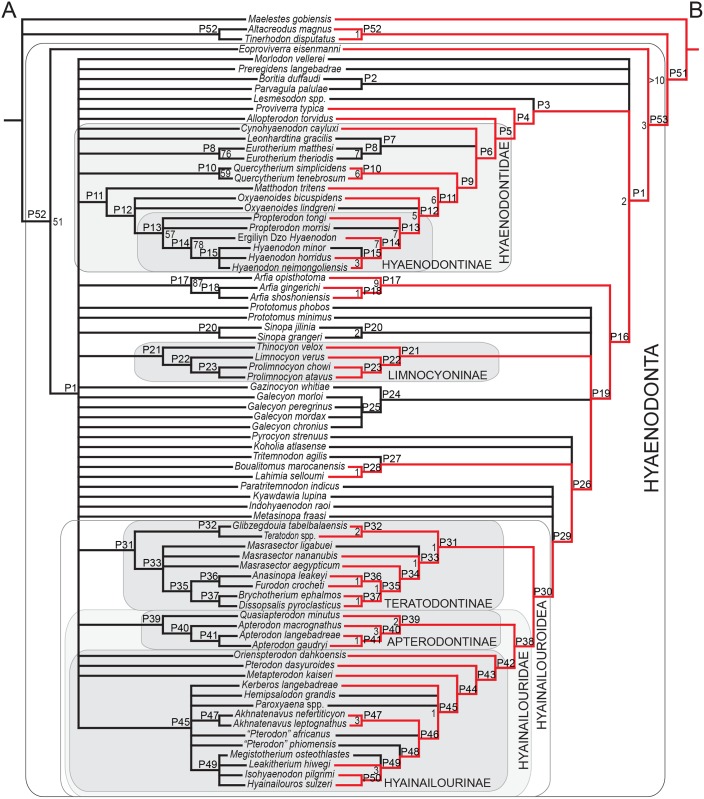
Parsimony consensus trees. Consensus trees of 1262 most parsimonious trees (MPTs), each with 1066 steps, a consistency index score (CI) of 0.181, and a retention index score (RI) of 0.617. P# next to each node is used in the discussion of the consensus trees. **A**, strict consensus tree with Bootstrap support great than 50% indicated next to the relevant node; **B**, Adams consensus tree with an agreement subtree for all MPTs indicated in red. The agreement subtree shows relationships recovered in all MPTs. Bremer support values are shown next to relevant nodes. Major clades discussed in this study are enclosed with the clade name by rounded boxes.

Maximum parsimony analysis recovered 1262 most parsimonious trees (MPTs), each with 1066 steps, a consistency index score (CI) of 0.181, and a retention index score (RI) of 0.617. The results are summarized in Fig 17 as a strict consensus tree and as an Adams consensus tree with the agreement subtree illustrated with the Adams consensus tree. The agreement subtree shows the relationships recovered in all MPTs.

*Masrasector* is not consistently resolved as monophyletic across all MPTs in the maximum parsimony analysis (P33) with the younger *Masrasector aegypticum* resolved in the Adams consensus tree and the agreement subtree as more closely related to the teratodontines *Anasinopa*, *Furodon*, *Brychotherium*, and *Dissopsalis* than the older species *Masrasector nananubis* and *Masrasector ligabuei*. The clade Teratodontinae (P31), which includes all species of *Masrasector*, is recovered in all MPTs, along with Apterodontinae (P39), a large clade of hyainailourines (P45), Limnocyoninae (P21), a clade that includes Hyaenodontinae (P11).

In the strict consensus tree, Teratodontinae is part of a polytomy with many other lineages of hyaenodonts (P1), but the Adams consensus tree and agreement subtree, show there is a consistent structure found in all MPTs that is disrupted by “wild card” taxa that include the European “proviverrine” taxa *Preregidens langebadreae*, *Boritia*, *Parvagula*, the North American and European taxa *Prototomus* and *Galecyon*, the North American taxon *Pyrocyon*, the Afro-Arabian taxa *Koholia*, *Metasinopa*, and the “indohyaenodontines” *Indohyaenodon*, *Paratritemnodon*, and *Kyawdawia*. Teratodontinae is recovered in the Adams consensus and agreement subtree as the sister clade of Hyainailouridae (Apterodontinae + Hyainailourinae) with all three clades–Teratodontinae, Apterodontinae, and Hyainailourinae–forming Hyainailouroidea. The European, North American, and Asian clade Hyaenodontidae is also resolved in the Adams consensus and agreement subtree.

### Results of morphometric analysis of distal humeri

**Fig 18:**
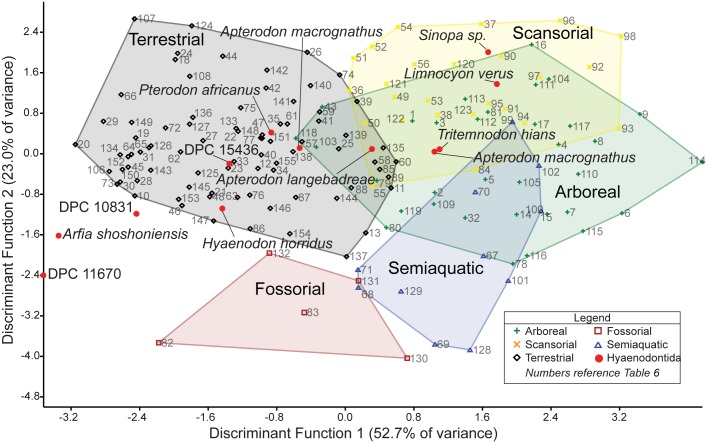
Locomotor categorization of hyaenodont humeri on DF1 and DF2. The results of discriminant function analysis for DF1 (explains 52.7% of variance) plotted against DF2 (explains 23.0% of variance). The analysis is based on 20 linear measurements (see Fig 2) collected for 155 carnivoran specimens (55 species) and 12 hyaenodont specimens (8 species). Numbers correspond to specimen numbers in Table 5, S4 and S5 Tables. Refer to Table 6 and S5 Table for classification of the hyaenodontan sample. Refer to Table 7 for measurements loadings. Each point represents a specimen, not a species mean. **Green plus**, Arboreal carnivoran specimens; **Yellow X**, Scansorial carnivoran specimens; **Black diamond**, Terrestrial carnivoran specimens; **Red squares**, Fossorial carnivoran specimens; **Blue triangles**, Semiaquatic carnivoran specimens; **Red dots**, hyaenodontans with unassigned locomotor behaviors.

**Fig 19:**
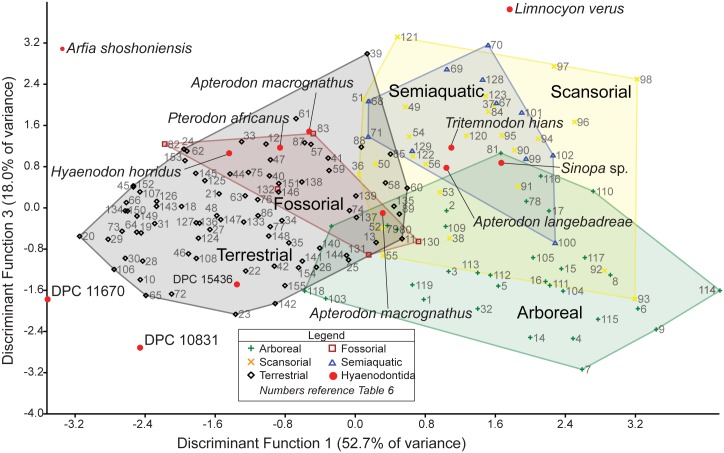
Locomotor categorization of hyaenodont humeri on DF1 and DF3. The results of discriminant function analysis for DF1 (explains 52.7% of variance) plotted against DF3 (explains 18.0% of variance). The analysis is based on 20 linear measurements (see Fig 2) collected for 155 carnivoran specimens (55 species) and 12 hyaenodont specimens (8 species). Numbers correspond to specimen numbers in Table 5, S4 and S5 Tables. Refer to Table 6 for classification of the hyaenodontan sample. Refer to Table 7 for measurements loadings. Each point represents a specimen, not a species mean. **Green plus**, Arboreal carnivoran specimens; **Yellow X**, Scansorial carnivoran specimens; **Black diamond**, Terrestrial carnivoran specimens; **Red squares**, Fossorial carnivoran specimens; **Blue triangles**, Semiaquatic carnivoran specimens; **Red dots**, hyaenodontans with unassigned locomotor behaviors.

The sample of modern Carnivora used in the discriminant function analysis of distal humeri is shown in Table 5 and the measurements collected from each specimen are shown in Fig 2 and listed with specimen numbers in S4 Table. The numbers for each specimen correspond to Fig 18, which shows the DF scores for each specimen along the axes defined by Discriminant Functions 1 and 2 and Fig 19, which shows the DF scores for each specimen along DF 1 and 3; the results for each specimen are listed in S5 Table. The hyaenodont sample, including the isolated humeri referred to *Masrasector* from L-41, are listed in Table 6 along with the probable assignment of each hyaenodont humerus to locomotor category. Table 7 shows the structure matrix for the analysis with the eigenvalue of each measurement for each DF. The relative loadings for each measurement for each DF is the absolute value of the eigenvalue, and the most heavily loaded variables for DF 1–3 are discussed below. Table 8 shows the confusion matrix based on the discriminant function analysis, with the percentage of accurately predicted specimens, the categories assigned to incorrectly categorized specimens, and the results of the leave-one-out cross-validation analysis. S1 Appendix shows and discusses the results of the DFA performed using species means rather than individual specimen measurements.

**Table 5:** Comparative sample of extant carnivoran humeri.

Numbers	Species	Common Name	Family	Locomotion (Secondary)
1–3	*Ailurus fulgens*	Red panda	Ailuridae	Arboreal (Scansorial)
4–6	*Arctictis binturong*	Binturong	Viverridae	Arboreal
7–9	*Arctogalidia trivirgata*	Small-toothed palm civet	Viverridae	Arboreal
10–13	*Atilax paludinosus*	Marsh mongoose	Herpestidae	Terrestrial (Semiaquatic)
14	*Bassaricyon gabbii*	Northern olingo	Procyonidae	Arboreal (Scansorial)
15–17	*Bassariscus astutus*	Ring-tailed cat	Procyonidae	Arboreal (Scansorial)
18–20	*Canis latrans*	Coyote	Canidae	Terrestrial
21–23	*Canis lupus*	Gray wolf	Canidae	Terrestrial
24–27	*Civettictis civetta*	African civet	Viverridae	Terrestrial (Scansorial)
28–31	*Crocuta crocuta*	Spotted hyaena	Hyaenidae	Terrestrial
32	*Cryptoprocta ferox*	Fossa	Eupleridae	Arboreal (Scansorial)
33–35	*Cynictis penicillata*	Yellow mongoose	Herpestidae	Terrestrial (Fossorial)
36–38	*Eira barbara*	Tayra	Mustelidae	Scansorial (Arboreal)
39–41	*Eupleres goudotii*	Falanouc	Eupleridae	Terrestrial
42	*Leopardus pardalis*	Ocelot	Felidae	Terrestrial (Scansorial)
43	*Leopardus wiedii*	Margay	Felidae	Arboreal (Scansorial)
44–48	*Fossa fossana*	Malagasy civet	Eupleridae	Terrestrial
49–51	*Galerella sanguineus*	Slender mongoose	Herpestidae	Scansorial (Terrestrial)
52–53	*Genetta genetta*	Common genet	Viverridae	Scansorial (Arboreal)
54–56	*Genetta maculata*	Rusty spotted genet	Viverridae	Scansorial (Terrestrial)
57–60	*Gulo gulo*	Wolverine	Mustelidae	Terrestrial (Fossorial)
61–63	*Herpestes ichneumon*	Egyptian mongoose	Herpestidae	Terrestrial (Fossorial)
64	*Hyaena brunnea*	Brown hyaena	Hyaenidae	Terrestrial
65–66	*Hyaena hyaena*	Striped hyaena	Hyaenidae	Terrestrial
67–69	*Lontra canadensis*	Northern river otter	Mustelidae	Semiaquatic (Fossorial)
70–71	*Lontra longicaudus*	Neotropical otter	Mustelidae	Semiaquatic (Fossorial)
72–73	*Lycaon pictus*	African wild dog	Canidae	Terrestrial
74–77	*Lynx rufus*	Bobcat	Felidae	Terrestrial (Scansorial)
78–80	*Martes americana*	American marten	Mustelidae	Arboreal (Scansorial)
81	*Martes pennanti*	Fisher	Mustelidae	Arboreal (Scansorial)
82–83	*Meles meles*	Eurasian badger	Mustelidae	Fossorial
84	*Melogale moschata*	Chinese ferret-badger	Mustelidae	Scansorial (Fossorial)
85–88	*Mephitis mephitis*	Striped skunk	Mephitidae	Terrestrial (Fossorial)
89	*Mungos mungo*	Banded mongoose	Herpestidae	Terrestrial (Fossorial)
90–93	*Nandinia binotata*	African palm civet	Nandiniidae	Scansorial (Arboreal)
94–98	*Nasua nasua*	South American coati	Procyonidae	Scansorial (Terrestrial)
99–102	*Neovison vison*	American mink	Mustelidae	Semiaquatic (Fossorial)
103–105	*Paguma larvata*	Masked palm civet	Viverridae	Arboreal (Scansorial)
106–108	*Panthera leo*	Lion	Felidae	Terrestrial
109–112	*Paradoxurus hermaphroditus*	Asian palm civet	Viverridae	Arboreal (Scansorial)
113	*Poiana richardsonii*	African linsang	Viverridae	Arboreal
114–118	*Potos flavus*	Kinkajou	Procyonidae	Arboreal
119	*Prionodon linsang*	Banded linsang	Viverridae	Arboreal (Scansorial)
120–123	*Procyon lotor*	Racoon	Procyonidae	Scansorial
124–127	*Proteles cristatus*	Aardwolf	Hyaenidae	Terrestrial
128–129	*Pteronura brasiliensis*	Giant otter	Mustelidae	Semiaquatic (Fossorial)
130–132	*Suricata suricatta*	Meerkat	Herpestidae	Fossorial (Terrestrial)
133–136	*Urocyon cinereoargenteus*	Grey fox	Canidae	Terrestrial (Scansorial)
137–139	*Ursus americanus*	American black bear	Ursidae	Terrestrial
140	*Ursus arctos*	Brown bear	Ursidae	Terrestrial
141–142	*Viverra tangalunga*	Malayan civet	Viverridae	Terrestrial
143–145	*Viverra zibetha*	Large Indian civet	Viverridae	Terrestrial
146–149	*Viverricula indica*	Small Indian civet	Viverridae	Terrestrial (Scansorial)
150–151	*Vulpes macrotis*	Kit fox	Canidae	Terrestrial
152–155	*Vulpes vulpes*	Red fox	Canidae	Terrestrial

Numbers correspond to Figs 18 and 19. Species included in this analysis were assigned locomotor categories based on Van Valkenburg [65], Samuels et al. [64], Nowak [66], and cross-referenced with http://animaldiversity.org [67]. Carnivorans are not easily confined to these broad locomotor categories, and the table includes secondary locomotor classifications from the literature for some species. Only primary locomotor categories were used in the DFA.

**Table 6:** Hyaenodont locomotor categories.

Taxon	Specimen	Age	Locality	Primary locomotion (%)	Secondary locomotion (%)
?*Masrasector nananubis*	DPC 10831	latest Eocene	L-41, Fayum, Egypt	Terrestrial (99.5%)	Arboreal (0.5%)
?*Masrasector nananubis*	DPC 11670	latest Eocene	L-41, Fayum, Egypt	Terrestrial (99.9%)	Arboreal (0.1%)
?*Masrasector nananubis*	DPC 15436	latest Eocene	L-41, Fayum, Egypt	Terrestrial (95.1%)	Arboreal (4.4%)
*Arfia shoshoniensis*	AMNH 15515	early Eocene	Willwood Fm., Wyoming	Terrestrial (100.0%)	Scansorial (0.0%)
*Apterodon langebadreae*	BNHM M85318	late Eocene or early Oligocene	Dur At Talah, Libya	Scansorial (43.5%)	Semiaquatic (27.7%)
*Apterodon macrognathus*	BNHM M9257	early Oligocene	Quarry A, Fayum, Egypt	Terrestrial (78.4%)	Scansorial (21.2%)
*Apterodon macrognathus*	BNHM M8440B	early Oligocene	Quarry A, Fayum, Egypt	Terrestrial (37.2%)	Scansorial (34.1%)
*Hyaenodon horridus*	AMNH 1381	early Oligocene	White River Fm., SD	Terrestrial (99.5%)	Scansorial (0.04%)
*Limnocyon verus*	AMNH 12155	middle Eocene	Bridger Fm., Wyoming	Scansorial (99.8%)	Semiaquatic (0.01%)
*“Pterodon” africanus*	BNHM M8886	early Oligocene	Jebel Qatrani, Egypt	Terrestrial (86.7%)	Scansorial (13.1%)
*Sinopa* sp.	AMNH 11533	middle Eocene	Bridger Fm., Wyoming	Scansorial (96.7%)	Semiaquatic (2.0%)
*Tritemnodon hians*	AMNH 16821	middle Eocene	Bridger Fm., Wyoming	Scansorial (58.9%)	Semiaquatic (28.0%)

Hyaenodont specimens with locomotor category determined by discriminant function analysis. Primary locomotion is the most likely locomotor category for the humerus given the DFA; Secondary locomotion is the second-most likely locomotor category for the humerus.

**Table 7:** Pooled within-groups correlations.

Measurement	DF1 (52.7%)	DF2 (23.0%)	DF3 (18.0%)
AW	0.22	0.14	-0.57*
CDW	-0.18	-0.10	0.04
CW	0.15*	0.03	-0.08
LatPT	0.12	0.08	-0.08
LEL	0.05	-0.10	0.06
LEW	0.03	0.10*	0.05
MaxAT	-0.39*	**0.30**	-0.19
MaxC	**-0.35**	-0.06	-0.19
MEA	0.16*	-0.11	-0.12
MedPT	-0.08	0.30*	0.19
MEL	0.48*	-0.10	**0.32**
MEW	-0.17	-0.01	0.01
MinAT	-0.35*	0.11	-0.16
MinDT	-0.44*	**-0.21**	-0.08
MinPT	0.02	0.03	0.03
OFH	-0.22*	0.04	0.07
OFW	0.16	-0.39*	-0.10
PTW	0.12	-0.20*	-0.03
SCW	0.18	-0.14	0.31*
TDW	-0.55*	0.08	-0.17
TW	0.06	0.12	-0.46*

Pooled within-group correlations between humeral measurements and discriminant functions.

* indicates largest correlation with discriminant function. Bolded eigenvalues are the most heavily loaded four measurements for each discriminant function.

**Table 8:** Confusion matrix.

Observed group (Total)	Arboreal	Scansorial	Terrestrial	Fossorial	Semiaquatic	% Correct
Arboreal (33)	27 (22)	1 (4)	3 (4)	0 (0)	2 (1)	81.8 (66.7)
Scansorial (25)	4 (4)	20 (7)	0 (2)	0 (0)	1 (1)	80.0 (72)
Terrestrial (81)	2 (4)	4 (7)	72 (62)	2 (5)	1 (3)	88.9 (76.5)
Fossorial (5)	0 (0)	0 (0)	0 (0)	5 (4)	0 (1)	100 (80.0)
Semiaquatic (11)	1 (1)	2 (2)	0 (0)	0 (1)	8 (7)	72.7 (63.6)
					Total	85.2 (72.9)

Confusion matrix with taxa sorted into correct group (taxa sorted into correct group using leave-one-out cross-reference analysis). Parentheses indicate number of specimens sorted into group in leave-one-out cross-validation analysis.

Discriminant Function 1 (DF1) accounts for 52.7% of the total variance in the sample. The most heavily loaded variables are the maximum anteroposterior depth of the trochlea (TDW, eigenvalue = -0.55), the mediolateral width of the medial epicondyle (MEL, eigenvalue = 0.48), the minimum anteroposterior depth of the trochlea (MinDT, eigenvalue = -0.44), and the maximum proximodistal height of the capitulum (MaxC, eigenvalue = -0.35). DF1 primarily discriminates taxa classified as Terrestrial and Fossorial from taxa classified as Scansorial, Arboreal, and Semiaquatic. At the extreme negative end of DF1 are *Canis latrans* (coyote, specimen 20, Terrestrial), *Crocuta crocuta* (spotted hyena, specimen 29, Terrestrial), *Lycaon pictus* (African painted dog, specimen 73, Terrestrial), and *Panthera leo* (lion, specimen 106, Terrestrial). At the extreme positive end of DF1 are *Potos flavus* (kinkajou, specimen 114, Arboreal), *Arctogalidia trivirgata* (small-toothed palm civet, specimen 9, Arboreal), *Arctictis binturong* (binturong, specimen 9, Arboreal), and *Nasua nasua* (coati, specimen 98, Scansorial). Along DF1, all three L-41 humerus specimens fall within the area of the axis occupied by terrestrial and fossorial carnivorans. DPC 11670 has a score of -3.52 and is closest to the hyaenodont *Arfia shoshoniensis* and the carnivoran *Canis latrans* (coyote, specimen 20, Terrestrial); DPC 10831 has a score of -2.45 and is closest to the hyaenodont *Arfia shoshoniensis* and the carnivorans *Canis latrans* (coyote, specimen 19, Terrestrial) and *Atilax paludinosus* (marsh mongoose, specimen 10, Terrestrial); and DPC 15436 has a score of -1.35 and is closest to the hyaenodont *Hyaenodon horridus* and the carnivoran *Canis lupus* (wolf, specimen 23, Terrestrial).

Discriminant Function 2 (DF2) accounts for 23.0% of the total variance in the sample. The most heavily loaded variables are the maximum mediolateral width of the olecranon fossa (OFW, eigenvalue = -0.39), the proximodistal height of the medial and posterior margin of the trochlea (MedPT, eigenvalue = -0.30), the maximum proximodistal height of the trochlea (MaxAT, eigenvalue = 0.30), and the minimum anteroposterior width of the trochlea (MinDT, eigenvalue = -0.21). DF2 primarily discriminates taxa classified as Terrestrial, Scansorial, and Arboreal from taxa classified as Fossorial and Semiaquatic. At the extreme negative end of DF2 are *Suricata suricatta* (meerkat, specimen 130), *Pteronura brasiliensis* (giant Brazilian otter, specimen 128), *Lontra canadensis* (North American river otter, specimen 69), and *Meles meles* (European badger, specimen 82). At the extreme positive end of DF2 are *Panthera leo* (lion, specimen 107), *Nasua nasua* (coati, specimen 96), *Eira barbara* (tayra, specimen 37), and *Proteles cristatus* (aardwolf, specimen 124). DPC 11670 has a score of -2.41 and occupies a region of the axis that is shared with Fossorial and Semiaquatic carnivorans. The closest carnivorans to DPC 11670 are *Neovison vison* (American mink, specimen 101, Semiaquatic) and *Lontra longicaudus* (Neotropical otter, specimen 71, Semiaquatic) and the closest hyaenodont is *Arfia shoshoniensis*. DPC 10831 has a score of -1.18 and occupies a region of DF2 that is shared with the negative range of the Terrestrial group and the negative range of the Arboreal group and the positive range of the Semiaquatic group. The closest carnivorans to DPC 10831 are *Bassariscus astutus* (ring-tailed cat, specimen 15, Arboreal) and *Arctictis binturong* (binturong, specimen 6, Arboreal) and the hyaenodont *Hyaenodon horridus*. DPC 15436 has a score of -0.20 and is closest to the carnivorans *Neovision vison* (American mink, specimen 102, Semiaquatic) and *Vulpes vulpes* (red fox, specimen 155, Terrestrial) and the hyaenodont *Apterodon langebadreae*.

Discriminant Function 3 (DF3) accounts for 18.0% of the total variance in the sample. The most heavily loaded variables are the mediolateral width of the distal articulation (AW, eigenvalue = -0.57), the mediolateral width of the trochlea (TW, eigenvalue = -0.46), the mediolateral length of the medial epicondyle (MEL, eigenvalue = 0.32), and the maximum width of the supinator crest proximal to the medial epicondyle (SCW, eigenvalue = 0.31). DF3 doesn’t completely discriminate between the locomotor categories designated for this study, with the Terrestrial group spread across the range of the axis along with the Scansorial group. The Semiaquatic group clusters near the positive range of DF3 and the Arboreal group clusters closer to the negative range. The carnivorans distributed at the extreme negative end of DF3 are *Arctogalidia trivirgata* (small-toothed palm civet, specimen 7, Arboreal), *Arctictis binturong* (binturong, specimen 4, Arboreal), *Bassaricyon gabbi* (Northern olingo, specimen 14, Arboreal), and *Arctogalidia trivirgata* (small-toothed palm civet, specimen 9, Arboreal). The carnivorans at the extreme positive range of DF3 are *Procyon lotor* (raccoon, specimen 121, Scansorial), *Lontra longicaudus* (neotropical otter, specimen 70, Semiaquatic), *Explores goudotii* (falanouc, specimen 39, Terrestrial), and *Nasua nasua* (coati, specimen 97, Scansorial). All three L-41 humeri cluster in the negative region of DF1, a region occupied by Terrestrial and Arboreal taxa and the closest hyaenodont to all three L-41 specimens on DF3 is *Apterodon macrognathus* (BNHM M8440B). DPC 10831 has a score of -2.71 on DF3. Carnivorans with a score closest to DPC 10831 along DF3 are *Arctogalidia trivirgata* (small-toothed palm civet, specimen 7, Arboreal) and *Arctictis binturong* (binturong, specimen 4, Arboreal). DPC 11670 has a score of -1.78 and the carnivoran specimens with the closest DF3 scores are *Ailurus fulgens* (red panda, specimen 146778, Arboreal) and *Nandinia binotata* (African palm civet, specimen, 93, Scansorial). DPC 15436 has a score of -1.49 on DF3 and the carnivorans with the closest scores are *Prionodon linsang* (specimen 119, Arboreal) and *Bassariscus astutus* (specimen 16, Arboreal).

Using the scores for each of the L-41 specimens along all three discriminant function axes, the Euclidean distances between each specimen and all other specimens in the analysis were calculated. In this three-dimensional space (DF1, DF2, and DF3), the carnivoran specimens in closest proximity to DPC 10831 are *Atilax paludinosus* (marsh mongoose, specimen 10, Terrestrial), *Canis lupus* (gray wolf, specimen 23, Terrestrial), *Hyaena hyaena* (brown hyena, specimen 65, Terrestrial), and *Panthera leo* (lion, specimen 106, Terrestrial). Overall, the shortest Euclidean distances in three-dimensional space between DPC 11670 and carnivoran specimens are between DPC 11670 and *Atilax paludinosus* (marsh mongoose, specimen 10, Terrestrial), *Crocuta crocuta* (spotted hyena, specimen 30, Terrestrial), *Panthera leo* (lion, specimen 106, Terrestrial), and *Crocuta crocuta* (spotted hyena, specimen 28, Terrestrial). Finally, the closest carnivoran specimens to DPC 15436 in DF1-DF3 space are *Vulpes vulpes* (red fox, specimen 155, Terrestrial), *Canis lupus* (gray wolf, specimen 23, Terrestrial), and *Potos flavus* (kinkajou, specimen 118, Arboreal).

The discriminant function analysis correctly classified 85.2% of the sample (72.9% in leave-one-out cross-validation analysis). The analysis most accurately predicted the Fossorial group with 100% of the specimens correctly classified in the full analysis and only one specimen misclassified as Semiaquatic in the cross-validation analysis. 88.9% of Terrestrial specimens were correctly classified (76.5% in cross-validation analysis), with most misclassifications being in the Scansorial category. 81.8% of the Arboreal specimens were correctly classified (66.7% in cross-validation analysis) with most misclassifications being in the Arboreal category. 80.0% of the Scansorial specimens were accurately classified, but only 72.7% of the Semiaquatic specimens were correctly classified (63.6% in cross-validation analysis).

All three L-41 specimens were classified as Terrestrial with greater than 95% probability. The secondary classification of each specimen was Arboreal but the probability of their inclusion in the Arboreal category was less than 5%. The other Afro-Arabian humeri included in this analysis were each categorized as Terrestrial but with low probabilities (*Apterodon langebadreae*, 43.5% probability Terrestrial and 27.7% scansorial; *Apterodon macrognathus* [BNHM M8440B], 37.2% Terrestrial, 34.1% Scansorial, and 28.6% Arboreal; *Apterodon macrognathus* [BNHM M9257] 78.4% Terrestrial, 21.2% Scansorial; “*Pterodon*” *africanus* [BNHM M8886], 86.7% Terrestrial, 13.1% Scansorial category). Of the five North American hyaenodonts that were also included in the DFA, *Arfia shoshoniensis* was unambiguously classified as Terrestrial (100%), as was *Hyaenodon horridus* (99.5%). *Limnocyon verus* (99.8%), *Sinopa* (96.7%), and *Tritemnodon hians* (58.9%) were all classified as Scansorial.

## Discussion

### Phylogenetic position of *Masrasector nananubis*

Phylogenetic hypotheses related to *Masrasector* have been complicated since the genus was first described over four decades ago. In their initial description, Simons and Gingerich [23] hypothesized that *Masrasector* was more derived than *Sinopa* from North America (at the time *Sinopa* was only known from North America) and *Proviverra* from Europe, and was possibly the ancestor of the younger African genera *Metasinopa* and *Anasinopa*. Lewis and Morlo [8] tentatively placed *Masrasector* in Hyainailourinae based on the Simons and Gingerich [23] hypothesis that *Masrasector* is the ancestor of *Metasinopa* and *Anasinopa*, though other authors have posed alternatives. Barry’s [27] analysis resolved *Masrasector aegypticum* as the sister taxon of *Proviverra*, although he did not place *Masrasector aegypticum* close to *Anasinopa*. Egi et al.’s [28] analysis placed *Masrasector* as the sister taxon of *Kyawdawia*; those authors constructed a composite *Masrasector* OTU that included the specimens that Holroyd [78] referred to *Masrasector ligabuei* from L-41 (here assigned to *Masrasector nananubis*). Egi et al. [28] found *Anasinopa* and *Dissopsalis* to be sister taxa and part of a larger clade that included *Masrasector* + *Kyawdawia*, *Metasinopa*, African ‘*Sinopa*’ (*Brychotherium ephalmos* in [27]), and *Paratritemnodon* as the so-called “Afroasian proviverrines.” Peigné et al. [84] disputed this hypothesis by describing new features for *Kyawdawia* based on more material from the Pondaung Formation in Myanmar. Peigné et al. [84] placed *Masrasector* as the sister taxon to *Prototomus* and *Sinopa*, though they did not base this placement on a reproducible analysis of a character-taxon matrix. In the Peigné et al. [84] hypothesis, *Masrasector* was unrelated to their “Hyainailourinae” (African “*Sinopa*” [= *Brychotherium*], *Metasinopa*, *Dissopsalis*, and *Anasinopa*) and its sister group “*Arfia-*like South Asia Proviverrinae” (*Kyawdawia* and *Paratritemnodon*). In the first cladistic analysis to include *Masrasector* since Egi et al. [28], Solé et al. [22] placed *Masrasector* as the sister taxon to *Teratodon* within Teratodontinae, which was the sister group of Proviverrinae and *Arfia*. In contrast to the previous studies, the hypothesis of Solé et al. [22] implies dispersal between Europe and Afro-Arabia, rather than between Asia and Afro-Arabia. Finally, Rana et al. [29] recovered Teratodontinae as a clade, but with *Glibzegdouia* placed as the sister taxon of *Masrasector*. The *Masrasector* + *Glibzegdouia* clade was deeply nested within Teratodontinae, and *Dissopsalis*, a taxon with no lower molar metaconids and a very small talonid on M_3_, was placed as the earliest lineage to branch from Teratodontinae, implying an extensive ghost lineage for that genus that stretches from the early Eocene to the middle Miocene. They also found indohyaenodontines (*Indohyaenodon*, *Paratritemnodon*, and *Kyawdawia*) to be paraphyletic with respect to Hyainailouroidea.

The Bayesian phylogenetic analyses presented here support the monophyly of *Masrasector*. Tip-dating analysis recovered the divergence of the *Masrasector* lineage from the rest of Teratodontinae as having occurred in the Bartonian (late middle Eocene), suggesting that additional *Masrasector*-like species should be present in other late middle Eocene, late Eocene, and early Oligocene localities in Afro-Arabia, a hypothesis borne out by the descriptive work of Solé et al. [25] who identified *Masrasector* dental material in the latest Bartonian or earliest Priabonian locality of Bir el-Ater in Algeria. This analysis also supports the distinction of *Masrasector nananubis* from *Masrasector ligabuei*, which are not consistently recovered as sister taxa using Bayesian methods. This contradicts Holroyd’s [78] hypothesis that the same species of *Masrasector* were present at L-41 (Priabonian, late Eocene, Egypt) and at Taqah (Rupelian, early Oligocene, Oman).

The results of the standard Bayesian analysis show similarities to the parsimony-based results of both Solé et al. [22] and Rana et al. [29] in placing *Masrasector* in a clade with *Glibzegdouia* and *Teratodon* to the exclusion of other teratodontines, but the broader placement of Teratodontinae differs substantially from Solé et al. [22] by resolving Teratodontinae as a sister group of Hyainailouridae rather than of Proviverrinae and *Arfia*. These results also differ from those of Rana et al. [29] by placing Apterodontinae, rather than Teratodontinae, as the sister group of Hyainailourinae.

There is additional conflict between the topologies depending on which Bayesian method is employed to reconstruct the tree. Relationships within Teratodontinae differ between the two Bayesian phylogenetic methods, with the Miocene teratodontines resolved in a single clade with *Masrasector* as their sister clade in the tip-dating analysis, and the Miocene teratodontines placed as sister taxa of different Eocene teratodontines in the standard Bayesian topology. Especially relevant to discussions of the origin and dispersal of Afro-Arabian hyaenodonts are the phylogenetic positions of the southern Asian “indohyaenodontines” (*Kyawdawia*, *Paratritemnodon*, and *Indohyaenodon*), which differ in the Bayesian analyses presented here.

The maximum parsimony analysis does not recover a monophyletic *Masrasector*. Instead, different MPTs resolve different relationships among *Masrasector* species, but these species are closely clustered together in every MPT as part of Teratodontinae and as more closely related to *Anasinopa*, *Furodon*, *Dissopsalis*, and *Brychotherium* than to *Glibzegdouia* and *Teratodon*. Like in the Bayesian analyses, the “indohyaenodontines” do not find a consistent placement relative to hyainailouroids.

The maximum parsimony analysis, summarized using the Adams consensus tree and agreement subtree, reveals similarities with the standard Bayesian topology. The structure of Hyainailouroidea is similar, and the predominantly North American clades that include Limnocyoninae and *Sinopa* are more closely related to Hyainailouroidea than Hyaenodontinae. *Arfia* is more deeply nested within Hyaenodonta using maximum parsimony than it is in the Bayesian summary trees, but the genus is consistently resolved as a clade.

### Phylogeny of Afro-Arabian Hyaenodonta

The early diverging lineages in both Bayesian phylogenetic analysis have very low posterior probability support, similar to the results presented by Borths et al. [9]. Overall, the hyaenodont relationships resolved in the maximum parsimony analysis are similar the results found in the standard Bayesian analysis with the “wild card” taxa noted in the results section (i. e. *Pyrocyon*, *Preregidens*, *Boritia*, *Parvagula* etc.) creating the sharpest differences between the topologies. The composition of the clades supported by early diverging branches differ between the standard and tip-dating Bayesian analysis. The lack of a clear, well-supported resolution of relationships at the base of Hyaenodonta is consistent with the pattern that would be expected for a clade that is undergoing a rapid adaptive radiation across several continents [85]. Weak support at the basal nodes may also be influenced by limited character sampling that is possible for most early hyaenodonts, such as *Eoproviverra*, *Lahimia*, *Boualitomus*, *Tinerhodon*, and *Prolimnocyon chowi*, which are only known from isolated teeth and fragmentary dentaries. The ancestral morphology of the hyaenodont dentition is relatively simple, with a basic tribosphenic pattern, and adaptations for increased carnivory further simplify this tribosphenic pattern [86]. Whereas carnivorans only evolved one carnassial pair, leaving the other molars the potential to evolve greater dental complexity (e.g., Ursidae, Procyonidae, Canidae) or severely reduce or even lose their upper molars (e.g., Hyaenidae, Felidae) [87], hyaenodonts with multiple carnassial pairs tend to have all molars simplified as dental shear increases [9]. The cranial and postcranial characters employed here, and by Rana et al. [29], provide improved resolution of the hyaenodont tree, and we expect that more information from cranial and postcranial fossils of hyaenodonts and potential relatives of Hyaenodonta will further aid investigations of this group’s phylogeny.

Tip-dating analysis suggests that (given the constraints of our age priors) there were many splits in the Paleocene that occurred when morphological evolution was particularly rapid, prior to the appearance of the major, better resolved, lineages in Hyaenodonta. There is only very weak support for most of these early branches. This rapid period of evolution might reflect the opening of carnivorous niche space after the K-Pg extinction event and the extinction of many carnivorous, terrestrial archosaurs [51, 55] that was to be filled ultimately not only by hyaenodonts but also members of Carnivora, Mesonychia, and Oxyaenida in North America, Europe, and Asia. In Afro-Arabia, the hyaenodont immigrants from Europe [9] rapidly filled carnivorous niches, especially occupying specialized carnivorous niches, as exemplified by *Lahimia* and *Koholia* [88].

### Cranial anatomy of Hyaenodonta

The addition of detailed cranial information from a teratodontine supports the position of *Masrasector* and its closest relatives as part of a clade that includes Apterodontinae and Hyainailourinae (i.e., Hyainailouroidea), and contradicts the tentative placement of Teratodontinae in Hyaenodontidae, as recently suggested by Solé et al. [30]. The most easily recognized cranial trait that unites Apterodontinae and Hyainailourinae is the wedge-shaped nuchal crest that slopes medially toward the foramen magnum [6, 30]. The nuchal crest of *Masrasector* is not as clearly wedged as the crests of *Pterodon dasyuroides*, *Hemipsalodon*, *Kerberos*, *Akhnatenavus*, and *Apterodon*, but the nuchal crest of *Masrasector* nevertheless more closely resembles the caudally deflected crests of these hyainailourids than the tall nuchal crests of *Hyaenodon*, *Cynohyaenodon*, and *Eurotherium* that are oriented toward the mastoid processes. As Polly [6] first noted, the differences in nuchal crest morphology between these groups indicates very different arrangements of the nuchal musculature in the two distinct hyaenodont lineages. *Hyaenodon* and other Proviverrinae/Hyaenodontinae taxa have nuchal morphology that is more characteristic of other mammals (e. g., [6, 89]), with a broad nuchal line and broad attachment sites for the cranial insertions of trapezius, semispinalis capitis, splenius capitis, and suboccipital muscles. *Pterodon*, *Apterodon*, and other hyainailourids must have organized these muscles in a different manner than Proviverrinae/Hyaenodontinae, with a division between muscles that attached to the narrowed nuchal crest and muscles that attached to the broad paroccipital process. The narrowing of the nuchal crest in Hyainailouridae also suggests a restructuring of the origin of some caudal fibers of the temporalis muscle that originated in part from the narrow nuchal crest. The morphology seen in *Masrasector* may represent a kind of intermediate stage between the nuchal morphology of basal hyaenodontids and that of Hyainailouridae. Given this hypothesis of intermediacy, and the phylogenetic hypotheses presented here, the nuchal morphology of an “indohyaenodontine” would be particularly important for our understanding of hyainailouroid nuchal morphology. Alternatively, the less wedge-shaped nuchal crest of *Masrasector* may be related to the small body size of the taxon with the muscular arrangement that unites Hyainailouridae expressed on a smaller cranium. Both *Sinopa* and *Tritemnodon* are closer in body size to *Masrasector*, and both have nuchal crests that are wide across their dorsal margin but narrow ventrally, trending medially toward the foramen magnum, but preserve a thin nuchal line to the mastoid process. Small taxa from the Proviverrinae/Hyaenodontinae clade, such as *Cynohyaenodon*, *Eurotherium*, and *Allopterodon* have nuchal morphology similar to that of *Hyaenodon*.

The morphology of the jugular foramen and the wide gap between the petrosal and exoccipital that is well preserved in *Pterodon dasyuroides* (first discussed by Lange-Badré [14]) is present in *Masrasector nananubis*. Many of the other features that link *Masrasector* to Hyainailouridae are illustrated by Solé et al. [30], such as the robust zygomatic arch with an extensive jugal-squamosal suture, reduced postorbital processes, multiple mental foramina, and a distinct preglenoid process. Mellett [4] detailed the morphological transitions that led to the very specialized cranial anatomy of *Hyaenodon*, including the low coronoid process, rounded mandibular condyle that sits ventral to the tooth row, and the delicacy of the zygomatic arch. Mellett [4] concluded that these cranial features were the result of a shift to the lateral pterygoid musculature as the dominant mandibular adductor, and diminution of the masseter muscle, which would have potentially been overly stretched and weak when the animal opened its jaws with a very wide gape (compared to those of carnivorans). Mellett [4] saw the posterior extension of the narial tube as an adaptation for a broad area of origin for the pterygoid musculature. He did not discuss the deeply excised and well-defined masseteric fossa on the dentary that complicates this pterygoid-dominated adductor model. A deep masseteric fossa with a deeply excised ventral margin expands the surface area for the insertion of the masseter, so perhaps there was still a substantial function for the masseter but its fibers were arranged in a different way than they are in Carnivora and Hyainailouridae. *Pterodon dasyuroides*, *Apterodon macrognathus*, and *Masrasector nananubis* arranged the adductor musculature differently; these taxa have dorsoventrally deep zygomatic arches, indicative of a more substantial role for the masseter in jaw adduction. These taxa also do not have narial tubes that are sutured to the basicranium. The broad palatines do meet medially in *Apterodon* and *Pterodon dasyuroides*, but they separate and flare laterally and ventrally, orienting the origin for the lateral pterygoids at a different angle than those of *Hyaenodon*.

### Locomotor diversity among Afro-Arabian hyaenodonts

The humeral specimens referred to *Masrasector* are the smallest hyaenodont postcrania ever described from Afro-Arabia, and all three are classified by discriminant analysis as belonging to a predominantly terrestrial taxon. All other hyaenodont distal humeri known from Afro-Arabia are also classified in the Terrestrial group, aside from *Apterodon langebadreae*, which was classified as scansorial or possibly semiaquatic. Based on distal humeral morphology, there is no evidence for primarily fossorial or arboreal hyaenodonts in the Paleogene of Afro-Arabia. The North American hyaenodont sample includes taxa that were placed in both the Terrestrial and Scansorial groups, with *Hyaenodon horridus* placed in the Terrestrial group, as hypothesized by Mellett [4] based on comparisons to canids, while *Limnocyon verus* was classified into the Scansorial group. Gebo and Rose [13] hypothesized that *Prolimnocyon atavus* was a scansorial hyaenodont based on comparisons with scansorial carnivorans, particularly procyonids and mustelids, and hypothesized that other limnocyonines like *Thinocyon* and *Limnocyon* were also capable of arboreal locomotion. The results of the DFA support their hypothesis that Limnocyoninae was a clade of small-bodied scansorial to arboreal hyaenodonts.

In their description of *A*. *langebadreae*, a taxon known from multiple postcranial elements including the ulna, radius, femur, calcaneum, astragalus, and pelvis, Grohé et al. [11] compared the postcranial anatomy to Carnivora. They hypothesized that *Apterodon langebadreae* was a semiaquatic or fossorial taxon based on the ratio of the short ulna to the longer humerus, and the long olecranon process of the ulna, both forelimb features characteristic of mammals that are capable of powerful extension of the forearm [72], among other features. The results of this study do not fully contradict their findings. The supinator crest is relatively broad and the medial epicondyle is massive, providing large attachment sites for the manual and digital flexors and extensors, traits expected in both scansorial and semiaquatic taxa. The humeri referred to *Apterodon langebadreae* and *Apterodon macrognathus* are both robust, with deltopectoral crests that extend distally and give the humeral shaft a sinusoidal curvature and thick, triangular cross-section. The distal humerus of *Apterodon* also has a large medial epicondyle compared to the articular surface of the humerus. The robust humeral morphology of *Apterodon* contrasts with the elongate humeral shaft and small medial epicondyle of *“Pterodon” africanus*.

Carnivorans appear to be an appropriate comparative group for evaluation of hyaenodont locomotion, as the discriminant functions produced by the analysis largely placed hyaenodont humeri in carnivoran morphospace. The distal humeri from L-41 resemble cursorial and terrestrial distal humeri of *Crocuta crocuta* (spotted hyena) and *Atilax paludinosus* (marsh mongoose), though there were hyaenodont outliers, particularly *Arfia* and *Limnocyon* along DF3 and *Arfia* and DPC 11670 along DF1. There are morphological differences between hyaenodont and carnivoran humeri—in general, hyaenodont humeri are more robust than those of comparably sized carnivorans, and the medial epicondyle tends to be relatively large and project more directly medially. Based on the results of this study and comparisons between the L-41 humeral specimens and carnivorans, *Masrasector* was a small, terrestrial hyaenodont with a relatively stable elbow joint that supported a habitually extended elbow and limited the ability of the animal to supinate [68, 73]. Like a hyena, marsh mongoose, or wolf, *Masrasector* would have been a fast moving and possibly even cursorial animal.

Carbone et al. [90] demonstrated that carnivorans smaller than 21.5 kg tend to focus on prey less than half their own mass. Given its estimated body mass (~1.16 kg), *M*. *nananubis* likely pursued the hystricognathous rodents preserved at L-41 such as *Gaudeamus aslius*, *Gaudeamus hylaeus*, and *Acritophiomys bowni* [91], though the locomotor diversity of these taxa is currently unknown. Terrestrial *M*. *nananubis* may have also have been capable of pursuing the small-bodied and likely terrestrial hyracoids that are extraordinarily abundant at Quarry L-41 (*Saghatherium bowni* and *Thyrohyrax meyeri*, [92]). It seems unlikely that *Masrasector* would have been capable of efficiently pursuing acrobatic arboreal primates, such as *Proteopithecus* [93] and *Wadilemur* [94].

## Conclusions

*Masrasector nananubis* is a new species from the late Eocene locality of L-41 (Priabonian, ~34 Ma) in Egypt. The small species is about the size of a skunk (*Mephitis mephitis*) or genet (*Genetta genetta*) and retains the broad talonid basins and connate metaconids, short upper molar metastyles, and broad upper molar protocones of a generalist carnivore, comparable to those of a skunk or mongoose that supplement a diet of vertebrate prey with arthropods and some fruits, nuts, and other plant material [66, 67]. The distal humeri that are likely attributable to *Masrasector nananubis* preserve morphology that is consistent with the species having been a fast-moving and largely terrestrial carnivore; this conclusion is supported by multivariate morphometric analysis of distal humeral morphology.

*Masrasector* is resolved by Bayesian and parsimony phylogenetic analysis within the Afro-Arabian clade Teratodontinae. Teratodontines are closely related to Hyainailouridae (Apterodontinae + Hyainailourinae) and “indohyaenodontines,” but the relationships between Hyainailouridae, Teratodontinae, and Indohyaenodontinae differ depending on the phylogenetic method. A closer relationship of Teratodontinae to Hyainailouridae than to Hyaenodontinae or Proviverrinae is supported by the shared presence of a distinctive nuchal crest that narrows to the foramen magnum, an elongate neurocranium that lacks distinct postorbital processes, and a mid-cranium constriction of the parietals.

The cranium of *Masrasector nananubis* is the oldest known from an Afro-Arabian hyaenodont. Coupled with the extensive sample of dentaries and referred humeral specimens, *Masrasector nananubis* is one of the most complete Afro-Arabian hyaenodonts aside from *Apterodon*. With a large sample of specimens and a detailed record of multiple anatomical regions, *Masrasector* now shifts from being a fragmentary problem taxon to a cornerstone of character development for all future studies that explore the evolutionary history and ecological diversity of Hyaenodonta.

## Supporting information

S1 Table Character descriptions. Descriptions of characters and character states used in this analysis.
(DOCX)

S2 Table Hyaenodonta date references. A list of each OTU used in this analysis with sources for the geological age ranges used in the tip-dating Bayesian analysis and the specimens observed to score each OTU.
(DOCX)

S3 Table *Masrasector* tip-dating statistics. The most important statistical results for the discussion of the tip-dating analysis. The node code corresponds to Fig 16. The table includes support values for each clade, divergence age estimates that have been properly adjusted, and evolutionary rates for every lineage in the analysis.
(XLSX)

S4 Table Humerus measurements. Each raw measurement for each specimen used in the discriminant function analysis.
(CSV)

S5 Table DFA results. The discriminant function scores for each specimen in the analysis performed in SPSS and the likely locomotor classification for each specimen.
(XLSX)

S1 Dataset *Masrasector* mesquite matrix. The character-taxon matrix used in this analysis, formatted in the phylogenetics program Mesquite.
(NEX)

S2 Dataset MrBayes *Masrasector* standard Bayes input file. The MrBayes input file with parameters used for the standard Bayesian analysis.
(NEX)

S3 Dataset Standard Bayes *Masrasector* tree. The “allcompat” consensus tree output by MrBayes that is illustrated in Fig 15.
(TRE)

S4 Dataset MrBayes *Masrasector* tip-dating input file. The MrBayes input file with parameters used for the standard Bayesian analysis.
(NEX)

S5 Dataset Tip-dating *Masrasector* tree. The “allcompat” consensus tree output by MrBayes that is illustrated in Fig 16. This tree file contains additional statistical output not illustrated in Fig 16. The calculations necessary to derive absolute % change/Ma and the absolute median age are included in the discussion.
(TRE)

S6 Dataset *Masrasector* parsimony matrix. The TNT input file used for the maximum parsimony analysis.
(TNT)

S7 Dataset *Masrasector* most parsimonious trees. 1262 most parsimonious trees formatted for PAUP, the program used to calculate the Adams consensus and agreement subtrees.
(TRE)

S1 Appendix Species mean DFA results. Species means rather than individual measurements used to conduct a discriminant function analysis to classify hyaenodont distal humeri.
(DOCX)
