# Supplementary material for: Craniodental and humeral morphology of a new species of Masrasector (Teratodontinae, Hyaenodonta, Placentalia) from the late Eocene of Egypt and locomotor diversity in hyaenodonts
Source: PLoS One. 2017 Apr 19;12(4):e0173527. doi: 10.1371/journal.pone.0173527 (PMC5396875; doi:10.1371/journal.pone.0173527)
Supplement: S1 Table — Descriptions of characters and character states used in this analysis. (DOCX) [file pone.0173527.s001.docx]

Supplemental Table 1. Description of characters used in phylogenetic analysis

***Lower Dentition***

1. First mental foramen position: inferior to P_1_ (0); inferior to P_2_ (1). (Solé et al., 2014; Character 0)
2. Second mental foramen position: inferior to P_3_ (0); inferior to P_4_ (1). (Solé et al., 2014; Character 1)
3. P_1_: present (0); absent (1). (Solé et al., 2014; Character 2)
4. P_1_ root number: two roots (0); one root (1). (Polly, 1996; Character 13; Zack, 2011; Character 2; Solé et al., 2014; Character 2)
5. P_2_ talonid mesiodistal length: absent to short (0); elongate with distinct inflection separating postprotocristid from talonid (1). (modified Polly, 1996; Character 3; Egi et al., 2005; Character 34; Solé et al., 2014; Character 5)
6. P_2_ to P_3_ relative mesiodistal length: P_2_ shorter than P_3_ (0); P_2_ as long or longer than P_3_ (1). (Egi et al., 2005; Character 29; Solé et al., 2014; Character 6)
7. P_3_ inclination: perpendicular to horizontal ramus, tooth forms isosceles triangle in buccal view (0); tooth inclines distally, preprotocristid mesially convex (1).
8. P_3_ buccolingual width relative to mesiodistal length: width 33% of length (0); width 50% of length (1); width more than 50% of length (2).
9. P_3_ paraconid morphology: absent or small (0); developed with distinct postparacristid (1). (modified Polly, 1996; Character 6; Egi et al., 2005; Character 35; Solé et al., 2014; Character 7)
10. P_3_ talonid mesiodistal length: short, cusp-like (0); long, distinct inflection separating postprotocristid from talonid (1). (modified Polly, 1996; Character 4; Egi et al., 2005; Character 37; Solé et al., 2014; Character 8)
11. P_3_ entoconid: absent (0); present (1). (Solé et al., 2014; Character 9)
12. P_3_ to P_4_ relative mesiodistal length: P_3_ shorter than P_4_ (0); P_3_ as long or longer than P_4_ (1). (Egi et al., 2005; Character 31; Solé et al., 2014; Character 10)
13. P_4_ inclination: perpendicular to horizontal ramus, tooth forms isosceles triangle in buccal view (0); tooth inclines distally, preprotocristid mesially convex (1).
14. P_4_ paraconid morphology: present but poorly developed (0); paraconid well-developed (1); paraconid indistinct to absent (2). (modified Polly, 1996; Character 7; Solé et al., 2014; Character 11)
15. P_4_ metaconid: absent (0); present, usually weakly developed or ridge-like (1). (modified Solé et al., 2014; Character 12)
16. P_4_ entoconid: absent (0); present (1). (Solé et al., 2014; Character 14)
17. P_4_ hypoconid height: short, less than 33% of protoconid height (0); tall, more than 33% protoconid height (1). (modified Solé et al., 2014; Character 16)
18. P_4_ talonid basin: buccolingually compressed and shallow (0); buccolingually wide and deep (1); absent (2).
19. P_4_ precingulid and postcingulid: absent (0); present (1). (modified Solé et al., 2014; Character 18)
20. P_4_ relative height: mesiodistally longer than height (0); mesiodistally shorter than height (1); mesiodistal length and height subequal (2).
21. P_4_ height relative to molars: shorter than all molars (0); taller than M_1_ only (1); taller than M_2_ (2).
22. P_5_ presence: present (0); absent (1).
23. M_1_ and M_2_ entoconid morphology: well developed or bulbous (0); crestiform with visible apex (1); undifferentiated entocristid (2). (modified Zack, 2011; Charcter 23; Solé et al., 2014; Character 27)
24. M_1_ and M_2_ talonid depth: deep (0); shallow (1). (modified Zack, 2011; Character 25; Solé et al., 2014; Character 29)
25. M_2_ entocristid in lingual view: parallels hypocristid (0); present, stops before metaconid (lower than hypocristid) (1); weak ridge or absent (2).
26. M_3_ entocristid: parallels hypocristid (0); present, stops before metaconid (lower than hypocristid) (1); weak ridge or absent (2).
27. M_1_ and M_2_ talonid buccolingual width: narrow, less than 80% width of trigonid (0); wide, greater than 80% trigonid (1). (modified Solé et al., 2014; Character 28)
28. M_1_ mesiodistal length relative to M_2_: M_1_ length subequal or longer than M_2_ (0); M_1_ length less than M_2_ (1). (modified Zack, 2011; Character 26; Solé et al., 2014; Character 31)
29. M_1_–M_3_ trigonid height relative to talonid: trigonid tall on all molars, talonid less than 50% of trigonid height (0); trigonid low on all molars, talonid more than 50% of trigonid height (1); trigonid low on M_1_ and M_2_ (2). (modified Solé et al., 2014; Character 32)
30. M_3_ postprotocristid distal trend in buccal view: slopes mesial to distal (0); perpendicular to alveolus (1); slopes distal to mesial (overhangs talonid) (2).
31. M_2_ cristid obliqua orientation relative to mesiodistal axis: lingual to buccal trend (0); parallel to mesiodistal axis (1); buccal to lingual trend (2). (compare to Zack, 2011; Character 21)
32. M_2_ and M_3_ paraconid position relative to protoconid, angle defined relative to mesiodistal axis of mandible: directly mesial to protoconid,15 degrees (0); slightly lingual paraconid, 15.1 to 45 degree angle (1); strong lingual position, 45.1 to 60 degrees (2). *Ordered*
33. M_3_ postparacristid mesial to distal trend: steep slope to preprotocristid (“V” shaped acute angle) (0); shallow slope to preprotocristid (forms right angle with preprotocristid) (1); forms obtuse angle with preprotocristid (2). *Ordered*
34. M_2_ and M_3_ paraconid height relative to protoconid: paraconid significantly shorter than protoconid (0); paraconid slightly shorter than protoconid (1); paraconid and protoconid subequal in height (2).
35. M_3_ postparacristid to premetacristid in lingual view: postparacristid shorter than premetacristid (0); postparacristid subequal to premetacristid (1); postparacristid longer than premetacristid (2).
36. M_3_ postparacristid length to preprotocristid in buccal view (carnassial blade proportions): postparacristid much shorter than preprotocristid (30%) (0); postparacristid half length of preprotocristid (1); postparacristid more than half preprotocristid length (2); subequal lengths (3).
37. M_2_ and M_3_ metaconid expression: connate and connects to paraconid base (0); connate, separated from paraconid (1); fold or ridge (2); absent (3). *Ordered*
38. M_1_ metaconid: taller than paraconid (0); subequal to paraconid (1); shorter than paraconid or absent (2). *Ordered* (compare to Polly, 1996; Character 18)
39. M_2_ metaconid: taller than paraconid (0); subequal to paraconid (1); shorter than paraconid or absent (2). *Ordered* (compare to Polly, 1996; Character 18)
40. M_3_ metaconid: taller than paraconid (0); subequal to paraconid (1); shorter than paraconid or absent (2). *Ordered* (compare to Polly, 1996; Character 19)
41. M_2_ mesiodistal length to M_3_ length: M_2_ shorter than M_3_ (0); M_2_ subequal to M_3_ (1); M_2_ longer than M_3_ (2); M_3_ absent (3). *Ordered* (compare to Zack, 2011; Character 30)
42. M_2_ talonid mesiodistal length (% of total mesiodistal length): >40% (0); 40% to 30% (1); 29% to 21% (2); <20% (3). *Ordered*
43. M_3_ talonid mesiodistal length (% of total mesiodistal length): >40% (0); 40% to 30% (1); 29% to 21% (2); <20% (3). *Ordered*
44. M_3_ talonid: present, bears hypoconid and hypoconulid (0); present, only one distinct cusp (1); absent (2). *Ordered*
45. M_2_ buccal talonid margin: steep slope distal to mesial (0); shallow slope distal to mesial (1); parallel to alveolus (2); slopes mesial to distal (3). *Ordered*
46. M_3_ buccal talonid margin angle from highest point to lowest: steep slope distal to mesial (0); shallow slope distal to mesial (1); parallel to alveolus (2); slopes mesial to distal (3). *Ordered*
47. M_1_–M_3_ ectocingulid: weakly expressed to absent (0); distinct (1). (modified Solé et al., 2014; Character 34)
48. M_1_–M_3_ postcingulid: absent (0); present (1). (modified Solé et al., 2014; Character 35)
49. M_1_–M_3_ ectocingulid to postcingulid connection: separated (0); fused (1). (modified Solé et al., 2014; Character 36)
50. M_3_ talonid buccolingual width relative to M_2_ talonid width: equal (0); narrower (1).
51. Mandible inflection anterior to angular process (Solé et al., in press): present (0); absent (1).
52. Angular process morphology: distinct process with medial inflection (0); gently curved process in line with mandibular corpus (1); ventral inflection (2).
53. Mandibular condyle position: superior to M_3_ alveolus (0); directly distal to M_3_ alveolus (1); inferior to M_3_ alveolus (2).
54. Coronoid process shape: tall, anterior and posterior slopes similar (0); tall, posterior slope concave (1); low, rounded (2).
55. Anterior coronoid angle relative to horizontal ramus: near vertical, 90 to 100 degrees (0); slight posterior inclination, 100 to 110 degrees (1); strong posterior inclination, greater than 110 degrees (2).
56. Masseteric fossa depth: deeply excavated with strong anterior angle, inferior margin well-defined (0); rounded anterior margin, little inferior definition (1); deep fossa but poorly defined inferior margin (2).

***Upper Dentition***

*Deciduous Upper Dentition*

1. dP^3^ parastyle mesiodistal length: more than half metastyle length (0); less than half metastyle length (1).
2. dP^3^ metacone-paracone fusion: metacone distinct cusp (premetacrista slopes to metacone apex; See *Pterodon dasyuroides*) (0); metacone fused to paracone (premetacrista sub-horizontal; See *Apterodon*) (1).
3. dP^3^ paracone morphology: pre- and postparacrista similar in slope (apex isosceles triangle in buccal view) (0); preparacrista distally inclined (apex closer to right triangle in buccal view) (1).
4. dP^3^ metastyle notch: Small inflection between postmetacrista and metastyle (0); deep notch between postmetacrista and metastyle (1).
5. dP^3^ Protocone prominence: mesiodistal length shorter than buccolingual width (narrow) (0); mesiodistal length equal to or longer than buccolingual width (wide) (1). (modified from Bastl et al. 2014; Character 2)
6. dP^3^ lingual cingulum: present (distinct lingual connection between parastyle and protocone) (0); absent (faint or no connection between parastyle and protocone) (1). (modified from Bastl et al. 2014; Character 4)
7. dP^4^ main cusp height: paracone taller than metacone (0); Paracone subequal to metacone (1); Paracone shorter than metacone (2). *Ordered* (Modified from Bastl et al., 2014; Character 6)
8. dP^4^ protocone orientation: Protocone projects mesially to parastyle margin (0); Protocone projects lingually and does not align with parastyle (1).
9. dP^4^ ectoflexus: Deep and distinct inflection between metacone and metastyle (0); Indistinct or shallow inflection between metacone and metastyle (1).

*Adult Upper Dentition*

1. Upper incisor count: 4 or more (0); 3 or fewer (1).
2. Lateral-most upper incisor: incisiform, similar to mesial incisor (0); caniniform (1).
3. P^3^ lobe of the protocone: absent (0); present but small (1); protocone well-developed, individuated (2). (modified Polly, 1996; Charcter 9; Egi et al., 2005; Character 4; Solé et al., 2014; Character 39)
4. P^3^ root number: two roots (0); three roots (1). (Solé et al., 2014; Character 40)
5. P^3^ contact with P^4^ parastyle: P^3^ contacts or aligned with P^4^ parastyle (0); P^3^ framed by P^4^ parastyle (1).
6. P^4^ parastyle: distinct (0); very reduced to absent (1). (modified Egi et al., 2005; Character 8; Zack, 2011; Character 34; Solé et al., 2014; Character 41)
7. P^4^ protocone alignment: transversely aligned with paracone (0); mesially shifted relative to paracone (1). (Zack, 2011; Character 36; Solé et al., 2014; Character 42)
8. P^4^ protocone morphology: bulbous and distinct from paracone (0); weak separation from paracone, shelf to cingulum-like (1). (modified Polly, 1996; Character 10; Egi et al., 2005; Character 6/7; Solé et al., 2014; Character 43)
9. P^4^ metastylar blade (=postmetacrista): short (0); elongate (1). (modified Egi et al., 2005; Character 9; Zack, 2011; Character 35; Solé et al., 2014; Character 45)
10. P^4^ metastyle contact with M^1^: P^4^ metastyle braced buccally by M^1^ parastyle (0); P^4^ metastyle contacts mesial aspect of M^1^ parastyle (1).
11. M^1^ and M^2^ metastyle blade curvature: straight with carnassial notch (0); postmetacrista arcuate, no carnassial notch (1). (Zack, 2011; Character 42; Solé et al., 2014; Character 46)
12. M^1^ and M^2^ metastyle blade length: short, carnassial blade shorter than postmetacrista (0); intermediate, subequal to slightly longer than postmetacrista (1); elongate, greater than 1.5x length of postmetacrista (2). *Ordered* (Egi et al., 2005; Character 21; Solé et al., 2014; Character 47)
13. M^1^ mesiodistal length relative to M^2^: M^1^ subequal or longer than M^2^ (0); M^1^ shorter than M^2^ (1). (modified Solé et al., 2014; Character 50)
14. M^1^ and M^2^ premetaconule crista: present (0); absent (1). (modified Solé et al., 2014; Character 51)
15. M^1^ and M^2^ conules: metaconule and paraconule present (0); only paraconule present (1); metaconule and paraconule absent (2) (modified Solé et al., 2014; Character 53)
16. M^1^ and M^2^ precingulum and postcingulum: absent (0); present (1). (modified from Polly, 1996; Character 20; Egi et al., 2005; Character 26; Solé et al., 2014; Character 54)
17. M^1^ and M^2^ precingulum and postcingulum connection: separated (0); fused lingually (1). (Solé et al., 2014; Character 55)
18. M^1^ and M^2^ protocone morphology: Triangular, mesial and distal margins angled (0); parallel mesial and distal margins (1). (modified Egi et al., 2005; Character 14)
19. M^1^ and M^2^ protocone position relative to paracone and metacone: centered (0); mesially shifted (1). (modified Egi et al., 2005; Character 15; Solé et al., 2014; Character 56)
20. M^1^ and M^2^ metacone mesiodistal length relative to paracone: shorter (0); subequal (1); longer (2). (modified Egi et al., 2005; Character 12)
21. M^1^ and M^2^ metacone coronal cross section: circular (0); ovoid, slightly compressed buccolingually (1); ellipsoid, strong buccolingual compression (2).
22. M^2^ paracone compression: conical, rounded base (0); ovoid (slight buccolingual compression) (1); ellipsoid (strong buccolingual compression) (2).
23. M^1^ and M^2^ paracone and metacone separation: separated to base (0); fused between base and half of height (1); almost completely fused (2). *Ordered* (modified Polly, 1996; Character 28; Egi et al., 2005; Character 10; Zack, 2011; Character 40)
24. M^1^ and M^2^ paracone height: paracone taller than metacone (0); paracone as tall as metacone (1); paracone shorter than metacone (2). *Ordered* (modified Egi et al., 2005; Character 11; Zack, 2011; Character 41; Solé et al., 2014; Character 48)
25. M^1^ and M^2^ protocone height: shorter than paracone/metacone separation (0); same height as paracone/metacone separation (1); subequal to paracone/metacone apices (2). (compare to Zack, 2011; Character 49)
26. M^2^ parastyle: absent (0); present and shelf-like (1); present and distinct cusp (2). (modified Egi et al., 2005; Character 19)
27. M^1^ parastyle compared to M^2^: M^1^ parastyle relatively shorter than M^2^ parastyle (0); parastyle on M^1^ and M^2^ similar (1).
28. M^1^ and M^2^ buccal cingulum: absent (0); weak ridge along metastyle base (1); prominent shelf forms shallow basin between metastyle and cingulum (2). (modified Egi et al., 2005; Character 16/17)
29. M^2^ ectoflexus: strong, strong indentation (0); weak, slight curve (1); absent, straight (2). (modified Egi et al., 2005; Character 18)
30. Ectoflexus depth on M^1^ and M^2^: M^1^ ectoflexus shallower than M^2^ (0); same relative depth between M^1^ and M^2^ (1); M^2^ lacks metastyle (no ectoflexus) (2). *Ordered* (compare to Zack, 2011; Character 52)
31. M^3^ metacone: present (0); absent (1). (modified Polly, 1996; Character 15; Zack, 2011; Character 53; Solé et al., 2014; Character 58)

***Cranial Characters***

1. Nuchal crest morphology: medial to lateral trend from apex to mastoid (0); dorsolateral margin tapers medially with thin connection to exoccipital (1); lateral margins trend medially, very weak ridge connects to exoccipital (2). *Ordered* (modified Polly, 1996; Character 35)
2. Facial wing of the lacrimal: extensive (larger than orbit diameter) (0); moderate (slightly longer than orbit diameter) (1); reduced (shorter than orbit diameter) (2). (modified Polly, 1996; Character 36)
3. Foramen rotundum size: slightly larger than foramen ovale (0); much larger than foramen ovale (1). (Polly, 1996; Character 39)
4. Palatal rugosity or torus at distal margin of palate: well-expressed (0); smooth (1).
5. Zygomatic arch contact: short contact between zygomatic and squamosal (0); extensive contact between zygomatic and squamosal (1).
6. Superior squamosal morphology: superior and inferior margins parallel (0); torsion along superior margin (1).
7. Foramen ovale orientation: anterior orientation (0); palatal orientation (1).
8. Exoccipital condyle position: tall, lateral placement around foramen magnum (0); ventral placement around foramen magnum (1).
9. Notch between occipital condyles: ring-like with no rostral excavation (0); rounded indentation with condyles meeting medially below foramen magnum (1); deep excavation with occipital “processes” following notch (2).
10. Postmandibular process: vertical orientation (0); strong anterior curvature (1).
11. Posterior orbital process: present, strong expression (0); present, weak with frontal “peaked” (1); absent (2).
12. Frontal furrow: absent or indistinct (0); present and well-defined (1).
13. Palatine and pterygoid medial contact: parallel posterior to palatine torus (0); palatines trend medially or partially fuse (1); fused entire extent of palatines (2).
14. Pterygoid size: broad, ventral projection anteriorly extensive (0); short anterior extent, trends medially (1).
15. Frontoparietal suture in dorsal view: steep constriction (0); gentle curvature (1).
16. Lateral expansion of the mastoid process (Solé et al., in press): projects to midpoint of mandibular fossa (0); projects beyond mandibular fossa (1).
17. Mastoid/paroccipital process: short tubercle (0); well-defined, prong-like process (1).
18. Process at maxilla/jugal suture (Solé et al., in press): present (0); absent (1).
19. Squamosal constriction in dorsal view: present, squamosal does not extend laterally (0); absent, squamosal expanded laterally (1).
20. Squamosal ventral projection: same transverse plane as petrosal (0); ventral to petrosal (1).
21. Posterior braincase: broad lateral expansion (0); narrow (1).
22. Subarcuate fossa morphology (Polly, 1996): cup-shaped (see *Pterodon*) (0); shallow and horseshoe-shaped (see *Hyaenodon*) (1).
23. Bridge of the stylomastoid foramen primitivum: absent or slender (0); robust (1); roofed over with secondary stylomastoid foramen (2). (Polly, 1996; Character 40)
24. Mastoid sinus lateral to foramen stylomastoid primitivum: absent (0); present (1). (Polly, 1996; Character 41)
25. Ridge of bone dividing posterior petrosal sinus from foramen stylomastoid primitivum (Polly, 1996): present (0); reduced to absent (1). (Polly, 1996; Character 42)
26. Posterior petrosal sinus: absent (0); small (1); greatly inflated (2). (Polly, 1996; character 43)

***Postcrania***

*Humerus*

1. Humerus cross-section above brachial flange: triangular (0); rounded (1). (Polly, 1996; Character 44)
2. Brachial flange expression: medium (0); enlarged (1); reduced (2). (Polly, 1996; Character 47)
3. Entepicondylar foramen: present, rounded (0); present, elongate (1); absent (2).
4. Medial epicondyle: large (bulbous) (0); reduced (elongate) (1).
5. Capitulum morphology: rounded, clearly separated (0); cylindrical (1).
6. Greater tubercle of the humerus: prominent, taller than humeral head (0); subequal to height of humeral head (1).

*Ulna*

1. Proximal trochlear notch orientation: lateral position on shaft (0); medial position on shaft (1).
2. Radial notch orientation: curved and faces laterally (0); flattened and faces anteriorly (1). (Polly, 1996; Character 47)
3. Olecranon process length: longer than trochlear notch (0); subequal or shorter than trochlear notch (1).
4. Olecranon process orientation: projects medially (0); projects ventrally (1).

*Femur*

1. Third trochanter of femur: large (0); small (1). (modified Polly, 1996; Character 50)

*Astragalus*

1. Astragalar foramen: large (0); reduced (0). (Polly, 1996; Character 51)
2. Astragalar condyles divided by: shallow depression (0); well-defined fossa or groove (1)
3. Astragalar condyle orientation: oblique relative to astragalar neck (0); parallel to astragalar neck (1).
4. Astragalar head relative to condyles: horizontal orientation (0); slight vertical orientation (1). (Polly, 1996; Character 53)
5. Sustentacular facet connection to astraglar head: clearly separated from astragalar head (0); grades into articulation of astragalar head (1).
6. Sustentacular facet position on astragalar neck: plantar astragalar neck (0); medial astragalar neck (1).

*Calcaneum*

1. Peroneal tubercle: distinct and separated from cuboid facet (0); part of a flange that grades to cuboid facet (1).
2. Cuboid facet inclination: perpendicular to calcaneal neck (0); plantar inclination (1).
3. Calcaneal neck trend: dorsal and plantar margins parallel (0); tapers proximally to calcaneal tuberosity (1).
4. Astragalar facet angle: oblique orientation to calcaneal neck (0); parallels calcaneal neck (1). (modified Polly, 1996; Character 58)

LITERATURE CITED

Bastl, K., Nagel, D., Peigné, S. 2014. Milk tooth morphology of small-sized *Hyaenodon* (Hyaenodontidae, Mammalia) from the European Oligocene – evidence of a *Hyaenodon* lineage in Europe. Palaeontographica, Abt. A: Palaeozoology – Stratigraphy 303:61–84.

Egi, N., P. A. Holroyd, T. Tsubamoto, A. N. Soe, M. Takai, R. L. Ciochon. 2005. Proviverrine hyaenodontids (Creodonta: Mammalia) from the Eocene of Myanmar and a phylogenetic analysis of the proviverrines from the para-Tethys area. Journal of Systematic Palaeontology 3:337–358.

Polly, P. D. 1996. The skeleton of *Gazinocyon vulpeculus* gen. et comb. nov. and the cladistic relationships of Hyaenodontidae (Eutheria, Mammalia). Journal of Vertebrate Paleontology 16:303–319.

Solé, F., J. Lhuillier, M. Adaci, M. Bensalah, M. Mahboubi, and R. Tabuce. 2014b. The hyaenodontidans from the Gour Lazib area (?early Eocene, Algeria): implications concerning the systematics and the origin of the Hyainailourinae and Teratodontinae. Journal of Systematic Paleontology 12:303–322.

Zack, S. P. 2011. New species of the rare early Eocene creodont *Galecyon* and the radiation of the early Hyaenodontidae. Journal of Paleontology 85:315–336.
