# Supplementary material for: Craniodental and humeral morphology of a new species of Masrasector (Teratodontinae, Hyaenodonta, Placentalia) from the late Eocene of Egypt and locomotor diversity in hyaenodonts
Source: PLoS One. 2017 Apr 19;12(4):e0173527. doi: 10.1371/journal.pone.0173527 (PMC5396875; doi:10.1371/journal.pone.0173527)
Supplement: S1 Appendix — Species means rather than individual measurements used to conduct a discriminant function analysis to classify hyaenodont distal humeri. (DOCX) [file pone.0173527.s013.docx]

**S1 Appendix: Discriminant Function Analysis Conducted with Species Means**

In the analysis presented in the main text of this analysis, the discriminant function analysis (DFA) conducted to place the three isolated humeri from L-41 referred to *Masrasector nananubis* in a quantitative comparative context was performed using individual humerus specimens of extant carnivorans to build the comparative sample. Here, we present the same discriminate function analysis, but instead of using individual specimens (IS) for the comparative sample, we use species means (SM).

**Species Means Materials and Methods**

The species means for the analysis were calculated by taking the average of the transformed values (see main Materials and Methods section for transformation details) for each of the 21 measurements used in the IS analysis for each species (Fig 2). The individual hyaenodont specimens remained as individual cases. The average score for each species for each measurement is shown in SM-Table 1. Like the IS analysis, the SM DFA was executed in the statistical package IBM SPSS 22 using the same parameters. The extant carnivoran sample includes 55 species. The discriminate function scores were visualized in PAST 3.04.

**Species Means Results**

The discriminate function scores for the carnivoran species mean sample are shown in SM-Fig 1 and SM-Table 2. In SM-Fig 1 species means and hyaenodont distal humeri are plotted along DF1, which explains 46.1% of the variance in the extant sample, DF2, which explains 28.1% of the variance in the extant sample, and DF3, which explains 21.5% of the variance in the extant sample. Not visualized in SM-Figure 1 is the axis for DF4, which explains 4.3% of the variance in the sample. SM-Table 2 also shows the assigned locomotor group for each species in the sample and predicted group for each species based on the DFA, along with the predicted locomotor category for each hyaenodont humerus in the sample. SM-Table 3 is the structure matrix for the SM analysis with the eigenvalue for each measurement for each DF. SM-Table 4 shows the confusion matrix with the percentage of correctly classified species for the DFA and the accuracy of the analysis using leave-one-out cross-validation analysis.

**Discussion**

Compared with the individual specimen analysis presented in the main body of this study, the species mean analysis shows the mean values for each species in each locomotor category more closely clustered around the centroid for each locomotor group. This is expected is a variability in size for some of the species included in this analysis. Some of this size variability may reflect sexual dimorphism in a given species. Unfortunately, the sex associated with each of the of the individual humeri measured for this analysis was not recorded. Such sources of variability raise one of the issues with using species means for such a morphometric analysis: in a given population of a species of Carnivora, there may be separate mean sizes associated with males and females or with different populations in different regions of a species’s range. Pooling all variability into a single species means creates a hypothetical species mean that may not reflect the biology of any known specimen of that species. In the main body of this text, we elected to present a DFA based on individual specimens because this method treats each humerus as a separate morphological occurrence and we prefer to predict hyaenodont locomotor behavior based on a directly observable rather than hypothetical comparative sample.

In the SM DFA the hyaenodont sample falls far outside of the groups defined by the centroids of each locomotor group. This highlights the morphological differences present between hyaenodonts and carnivorans, though there is greater overlap between the hyaenodont sample and the carnivoran sample when the full range of morphological variability in the sample is compared with the fossil sample, as it is in the IS DFA.

Despite the differences between the species mean and individual specimen discriminate function scores for some carnivorans and the resulting position of hyaenodonts outside the group convex hulls, the predicted locomotion for all distal humeri referred to *Masrasector* in this study (DPC 10831, DPC 11670, DPC 15436) is Terrestrial (100% probability) as it was in the individual sample. The predicted locomotor behavior of all other hyaenodonts are also the same in both analyses, except for *Hyaenodon horridus* and *“Pterodon” africanus*. Both taxa in the IS DFA are classified as Terrestrial with high probabilities. In the SM DFA both taxa are classified as Scansorial (*Hyaenodon horridus*, 62.5%; *“Pterodon” africanus*, 100%) with their secondary classifications as Terrestrial.

In the SM DFA, the hyaenodont sample, including the distal humeri referred to *Masrasector,* occupy a different region of morphospace than many carnivorans, though the IS DFA demonstrates this is an artifact of constraining the variance in the comparative sample and carnivoran and hyaenodont distal humeri do occupy overlapping morphospace when the range of carnivoran morphology is considered as part of the classification analysis.

**SM-Table 1. Species Means for each measurement before transformation**

| **Fig Code** | **Name** | **AW** | **TW** | **CW** | **MaxAT** | **MinAT** | **MaxC** | **SCW** |
| --- | --- | --- | --- | --- | --- | --- | --- | --- |
| 1 | *Ailurus fulgens* | 15.92 | 5.33 | 9.09 | 9.08 | 6.44 | 8.19 | 5.99 |
| 2 | *Arctictis binturong* | 20.64 | 8.74 | 10.17 | 9.05 | 5.51 | 8.93 | 5.55 |
| 3 | *Arctogalidia trivirgata* | 14.05 | 6.27 | 6.68 | 6.11 | 4.08 | 5.42 | 4.73 |
| 4 | *Atilax paludinosus* | 10.24 | 3.9 | 5.28 | 6 | 4.83 | 5.6 | 3.64 |
| 5 | *Bassaricyon gabbii* | 10.58 | 4.8 | 5.71 | 4.4 | 3.06 | 4.5 | 3.09 |
| 6 | *Bassariscus astutus* | 9.11 | 3.5 | 5.05 | 4.76 | 3.17 | 4.37 | 2.49 |
| 7 | *Canis latrans* | 22.74 | 11.11 | 9.84 | 16.32 | 10.66 | 13.93 | 5.64 |
| 8 | *Canis lupus* | 30.53 | 15.82 | 12.98 | 20.46 | 14.73 | 18.96 | 7.63 |
| 9 | *Civettictis civetta* | 18.89 | 8.03 | 10.59 | 13.21 | 8.02 | 10.21 | 6.6 |
| 10 | *Crocuta crocuta* | 38.35 | 20.5 | 17.67 | 25.24 | 14.7 | 20.3 | 11.75 |
| 11 | *Cryptoprocta ferox* | 16.71 | 7.48 | 9.08 | 8.77 | 6.22 | 8.31 | 5.07 |
| 12 | *Cynictis penicillata* | 6.95 | 2.96 | 3.38 | 4.5 | 3.23 | 3.99 | 2.29 |
| 13 | *Eira barbara* | 18.74 | 6.59 | 11.68 | 10.45 | 6.79 | 9.16 | 7.21 |
| 14 | *Eupleres goudotii* | 9.33 | 3.99 | 5.06 | 5.93 | 3.67 | 4.81 | 3.75 |
| 15 | *Felis pardalis* | 21.14 | 9.07 | 11.92 | 12.66 | 8.17 | 10.45 | 6.43 |
| 16 | *Felis wiedii* | 12.58 | 5.66 | 6.92 | 8.26 | 4.95 | 6.52 | 4.25 |
| 17 | *Fossa fossana* | 8.43 | 3.45 | 4.8 | 6.04 | 3.99 | 4.97 | 2.63 |
| 18 | *Galerella sanguineus* | 5.9 | 2.26 | 3.4 | 3.87 | 2.3 | 3.09 | 1.88 |
| 19 | *Genetta genetta* | 10.04 | 3.32 | 6.45 | 6.13 | 3.73 | 4.95 | 3.2 |
| 20 | *Genetta maculata* | 9.53 | 3.55 | 5.75 | 5.36 | 3.32 | 4.38 | 3.36 |
| 21 | *Gulo gulo* | 23.82 | 8.72 | 14.94 | 12.9 | 9.15 | 13.3 | 10.47 |
| 22 | *Herpestes ichneumon* | 9.92 | 4.1 | 5.75 | 7.67 | 5.05 | 6.26 | 3.02 |
| 23 | *Hyaena brunnea* | 33.46 | 13.4 | 19.91 | 23.21 | 13.11 | 18.15 | 11.83 |
| 24 | *Hyaena hyaena* | 30.04 | 10.92 | 19.02 | 21.55 | 14.2 | 18.4 | 11.07 |
| 25 | *Lontra canadensis* | 15.12 | 5.37 | 9.66 | 6.96 | 5 | 7.91 | 8.7 |
| 26 | *Lontra longicaudus* | 13.43 | 3.83 | 9.46 | 7.46 | 5.84 | 7.45 | 7.64 |
| 27 | *Lycaon pictus* | 24.85 | 8.91 | 14.73 | 18.02 | 12.72 | 16.28 | 9.22 |
| 28 | *Lynx rufus* | 18 | 7 | 10.88 | 11.6 | 7.1 | 10.08 | 6.15 |
| 29 | *Martes americana* | 7.88 | 2.95 | 4.99 | 4.49 | 3.33 | 4.3 | 3 |
| 30 | *Martes pennanti* | 11.76 | 4.27 | 7.36 | 6.99 | 4.49 | 6.4 | 5 |
| 31 | *Meles meles* | 15.77 | 5.34 | 10.59 | 9.38 | 6.49 | 9.05 | 9.3 |
| 32 | *Melogale moschata* | 7.99 | 3.39 | 4.55 | 4.51 | 3.53 | 4.24 | 3.68 |
| 33 | *Mephitis mephitis* | 10.56 | 4.34 | 6.04 | 4.75 | 3.6 | 4.8 | 5.64 |
| 34 | *Mungos mungo* | 7.7 | 2.38 | 5.24 | 4.9 | 3.33 | 4.09 | 3.66 |
| 35 | *Nandinia binotata* | 12.48 | 5.24 | 7.05 | 5.7 | 4.15 | 5.45 | 4.7 |
| 36 | *Nasua nasua* | 13.94 | 5.4 | 8.54 | 7.94 | 4.91 | 6.6 | 7.04 |
| 37 | *Neovison vison* | 7 | 2.64 | 4.07 | 3.88 | 2.67 | 3.65 | 2.58 |
| 38 | *Paguma larvata* | 14.39 | 5.66 | 8.4 | 8.28 | 5.39 | 6.96 | 4.19 |
| 39 | *Panthera leo* | 53.9 | 25.11 | 29.2 | 31.61 | 16.7 | 28.2 | 19.63 |
| 40 | *Paradoxurus hermaphroditus* | 13.97 | 5.45 | 8.3 | 6.94 | 4.5 | 6.2 | 5.3 |
| 41 | *Poiana richardsoni* | 6.85 | 2.71 | 4.1 | 4.05 | 2.47 | 3.2 | 1.87 |
| 42 | *Potos flavus* | 14.16 | 6.05 | 8.05 | 5.68 | 4.14 | 6.09 | 5.73 |
| 43 | *Prionodon linsang* | 9.46 | 4.03 | 5.13 | 5.55 | 3.44 | 4.71 | 3.42 |
| 44 | *Procyon lotor* | 15.48 | 5.51 | 9.91 | 9.14 | 5.97 | 7.8 | 8.29 |
| 45 | *Proteles cristatus* | 18.36 | 6.25 | 11.84 | 12.67 | 8.76 | 11.84 | 6.57 |
| 46 | *Pteronura brasiliensis* | 19.93 | 7.62 | 11.98 | 9.95 | 7.41 | 12.62 | 10.3 |
| 47 | *Suricata suricatta 3* | 6.83 | 2.65 | 4.17 | 3.54 | 2.61 | 3.55 | 2.71 |
| 48 | *Urocyon cinereoargenteus* | 12.48 | 3.6 | 8.8 | 8.88 | 7 | 8.11 | 4.4 |
| 49 | *Ursus americanus* | 49.9 | 19.23 | 30.6 | 20.75 | 12.63 | 19.83 | 20.5 |
| 50 | *Ursus arctos* | 66.06 | 29.38 | 36.26 | 29.55 | 16.21 | 24.18 | 26.55 |
| 51 | *Viverra tangalunga* | 14.62 | 5.92 | 8.66 | 8.39 | 5.37 | 7.05 | 4.63 |
| 52 | *Viverra zibetha* | 15.5 | 5.43 | 10.1 | 10.46 | 6.56 | 8.78 | 6.4 |
| 53 | *Viverricula indica* | 9.27 | 3.31 | 5.71 | 6.4 | 4.2 | 5.04 | 3.29 |
| 54 | *Vulpes macrotis* | 9.1 | 3.26 | 5.66 | 6.73 | 4.87 | 6.14 | 2.9 |
| 55 | *Vulpes vulpes* | 12.22 | 4.26 | 7.82 | 9.48 | 6.478 | 8.67 | 4.65 |
|  | *Apterodon langebadreae* | 27.33 | 11.44 | 15.89 | 14.96 | 10.47 | 12.69 | 11.97 |
|  | *Hyaenodon horridus* | 27.68 | 12.08 | 14.06 | 22.93 | 15.82 | 19.64 | 11.42 |
|  | *Limnocyon verus* | 14.94 | 5.41 | 9.33 | 7.98 | 5.83 | 7.67 | 9.63 |
|  | *Sinopa* sp. | 14.92 | 5.86 | 9.32 | 10.69 | 7.04 | 7.27 | 7.38 |
|  | *Tritemnodon hians* | 19.06 | 8.3 | 10.62 | 7.25 | 7.1 | 8.1 | 8.34 |
|  | *Arfia shoshoniensis* | 10.57 | 5.97 | 4.08 | 10.24 | 7.29 | 9.08 | 4.41 |
|  | *Apterodon macrognathus* | 38.36 | 14.59 | 22.94 | 25.53 | 17.88 | 22.47 | 23.77 |
|  | *Apterodon macrognathus* | 34.95 | 12.59 | 22.58 | 16.72 | 13.7 | 16.07 | 17.72 |
|  | *Pterodon africanus* | 41.04 | 19.12 | 21.46 | 30.28 | 18.71 | 23.04 | 14.51 |
|  | DPC 10831 | 10.59 | 5.08 | 4.77 | 4.69 | 4.34 | 3.71 | 5 |
|  | DPC 11670 | 9.22 | 4.48 | 4.54 | 6.43 | 6.66 | 5.57 | 5.66 |
|  | DPC 15436 | 9.79 | 4.9 | 4.79 | 4.95 | 5.2 | 4.95 | 4.69 |
| **Fig Code** | **Name** | **LatPT** | **MedPT** | **MinPT** | **OFW** | **OFH** | **CDW** | **TDW** |
| 1 | *Ailurus fulgens* | 7.88 | 6.81 | 5.05 | 6.21 | 5.5 | 9.41 | 9.27 |
| 2 | *Arctictis binturong* | 9.88 | 7.65 | 5.62 | 10.96 | 7.32 | 11.4 | 10.49 |
| 3 | *Arctogalidia trivirgata* | 5.45 | 4.8 | 3.26 | 6.7 | 2.9 | 8.46 | 6.74 |
| 4 | *Atilax paludinosus* | 4.68 | 4.29 | 2.98 | 5.96 | 4.4 | 7.81 | 7.36 |
| 5 | *Bassaricyon gabbii* | 4.04 | 3.89 | 3.62 | 4.66 | 1.83 | 4.62 | 4.65 |
| 6 | *Bassariscus astutus* | 4.83 | 4.16 | 3.28 | 4.24 | 1.57 | 6.31 | 5.39 |
| 7 | *Canis latrans* | 11.24 | 13.6 | 7.65 | 10.94 | 13.13 | 21.71 | 24.4 |
| 8 | *Canis lupus* | 15.54 | 18.08 | 12.28 | 14.14 | 14.06 | 26.89 | 27.8 |
| 9 | *Civettictis civetta* | 9.66 | 9.57 | 6.23 | 9.08 | 6.88 | 12.63 | 14.45 |
| 10 | *Crocuta crocuta* | 15.91 | 14.27 | 11.05 | 19.9 | 21.17 | 23.08 | 28.42 |
| 11 | *Cryptoprocta ferox* | 8.53 | 5.98 | 5.77 | 9.43 | 6.61 | 10.82 | 9.47 |
| 12 | *Cynictis penicillata* | 3.6 | 3.47 | 2.4 | 3.36 | 3 | 4.72 | 4.93 |
| 13 | *Eira barbara* | 9.34 | 8.67 | 5.83 | 8.73 | 7.19 | 13.9 | 11.3 |
| 14 | *Eupleres goudotii* | 5.56 | 5.47 | 4.08 | 4.74 | 3.62 | 7.32 | 7.85 |
| 15 | *Felis pardalis* | 10.42 | 9.98 | 6.64 | 8.96 | 8.65 | 12.98 | 14.34 |
| 16 | *Felis wiedii* | 5.8 | 6.81 | 4.16 | 5.48 | 5.4 | 8.46 | 8.92 |
| 17 | *Fossa fossana* | 4.48 | 4.81 | 3.21 | 4.26 | 3.53 | 6 | 6.89 |
| 18 | *Galerella sanguineus* | 3.43 | 3.48 | 2.27 | 2.9 | 2.97 | 4.7 | 4.25 |
| 19 | *Genetta genetta* | 4.92 | 5.14 | 3.29 | 5.17 | 4.41 | 6.75 | 6.55 |
| 20 | *Genetta maculata* | 4.05 | 4.6 | 3.19 | 4.57 | 3.44 | 6.4 | 6.01 |
| 21 | *Gulo gulo* | 13.14 | 9.37 | 7.42 | 12.23 | 11.1 | 18.6 | 15.04 |
| 22 | *Herpestes ichneumon* | 5.38 | 5.46 | 3.51 | 6.04 | 4.95 | 8.4 | 7.79 |
| 23 | *Hyaena brunnea* | 13.36 | 14.64 | 9.74 | 18.42 | 18.42 | 21.9 | 21.07 |
| 24 | *Hyaena hyaena* | 13.61 | 14.46 | 9.62 | 14.06 | 19 | 22.95 | 23.44 |
| 25 | *Lontra canadensis* | 7.87 | 6.57 | 5.45 | 9.84 | 5.35 | 11.22 | 9.4 |
| 26 | *Lontra longicaudus* | 7.15 | 6.7 | 4.94 | 8.5 | 6.35 | 11.57 | 9.38 |
| 27 | *Lycaon pictus* | 12.9 | 13.16 | 9.16 | 13.33 | 17.64 | 19.49 | 22.71 |
| 28 | *Lynx rufus* | 9.63 | 8.7 | 5.95 | 9.94 | 11.78 | 12.81 | 13.59 |
| 29 | *Martes americana* | 4.42 | 3.29 | 2.99 | 5.2 | 4.4 | 6.04 | 5.09 |
| 30 | *Martes pennanti* | 6.26 | 4.93 | 4.34 | 5.6 | 8.9 | 8.13 | 7.26 |
| 31 | *Meles meles* | 7.96 | 6.15 | 5.26 | 10.91 | 10.28 | 12.7 | 12.54 |
| 32 | *Melogale moschata* | 4.73 | 4.49 | 3.51 | 5.45 | 4.23 | 5.48 | 4.62 |
| 33 | *Mephitis mephitis* | 4.68 | 5.16 | 3.78 | 6.93 | 4.64 | 6.61 | 7.21 |
| 34 | *Mungos mungo* | 3.61 | 3.59 | 2.63 | 4.44 | 3.92 | 5.21 | 5.33 |
| 35 | *Nandinia binotata* | 6.33 | 5.77 | 3.96 | 6.19 | 5.52 | 6.95 | 6.49 |
| 36 | *Nasua nasua* | 6.56 | 7.08 | 4.72 | 7.37 | 7 | 8.93 | 8.87 |
| 37 | *Neovison vison* | 3.92 | 3.46 | 2.8 | 4.01 | 3.46 | 5.49 | 4.23 |
| 38 | *Paguma larvata* | 7.23 | 6.54 | 4.71 | 7.55 | 6.56 | 9.71 | 9 |
| 39 | *Panthera leo* | 23.58 | 24.37 | 14.86 | 28.2 | 31.57 | 35.39 | 39.57 |
| 40 | *Paradoxurus hermaphroditus* | 6.49 | 5.43 | 4.3 | 7.39 | 7.32 | 8.42 | 8.24 |
| 41 | *Poiana richardsoni* | 3.57 | 3.27 | 2.41 | 3.23 | 2.64 | 4.08 | 4.27 |
| 42 | *Potos flavus* | 6.21 | 5.72 | 4.38 | 7.9 | 6.16 | 8.24 | 8.16 |
| 43 | *Prionodon linsang* | 4.51 | 3.34 | 2.97 | 6.02 | 3.22 | 5.82 | 6.12 |
| 44 | *Procyon lotor* | 9.2 | 9.32 | 6.1 | 8.45 | 10.94 | 10.79 | 10.76 |
| 45 | *Proteles cristatus* | 8.56 | 8.85 | 6.6 | 8.57 | 16.08 | 13.7 | 14.29 |
| 46 | *Pteronura brasiliensis* | 10.91 | 11.84 | 7.77 | 13.24 | 10.92 | 15.02 | 13.9 |
| 47 | *Suricata suricatta 3* | 3.21 | 2.91 | 2.38 | 4.56 | 3.97 | 4.69 | 4.8 |
| 48 | *Urocyon cinereoargenteus* | 7.65 | 8.57 | 5.33 | 6.42 | 8.36 | 9.9 | 10.51 |
| 49 | *Ursus americanus* | 16.84 | 17.7 | 12.11 | 20.72 | 27.95 | 28.52 | 27.74 |
| 50 | *Ursus arctos* | 21.64 | 17.52 | 13.65 | 32.39 | 34.68 | 32.67 | 38.31 |
| 51 | *Viverra tangalunga* | 6.6 | 7.58 | 4.85 | 6.61 | 6.1 | 8.33 | 9.58 |
| 52 | *Viverra zibetha* | 8.02 | 6.39 | 5.45 | 8.73 | 7.6 | 11.1 | 12.42 |
| 53 | *Viverricula indica* | 4.68 | 4.01 | 3.4 | 5.52 | 5.86 | 7.21 | 7.41 |
| 54 | *Vulpes macrotis* | 5.45 | 6.21 | 4.58 | 4.78 | 5.78 | 7.42 | 8.26 |
| 55 | *Vulpes vulpes* | 7.93 | 7.94 | 5.87 | 6.1 | 8.56 | 10.44 | 11.5 |
|  | *Apterodon langebadreae* | 11.51 | 15.17 | 8.95 | 18.98 | 13.3 | 15.81 | 18.16 |
|  | *Hyaenodon horridus* | 15.51 | 18.41 | 12.7 | 18.33 | 17.05 | 20.89 | 21.78 |
|  | *Limnocyon verus* | 8.91 | 10.22 | 7.61 | 9.85 | 11.81 | 11.05 | 10.19 |
|  | *Sinopa* sp. | 7.97 | 9.75 | 5.67 | 8.85 | 7.62 | 9.35 | 10.03 |
|  | *Tritemnodon hians* | 9.17 | 11.77 | 7.75 | 12.08 | 10.21 | 11.64 | 11.49 |
|  | *Arfia shoshoniensis* | 8.31 | 9.1 | 6.85 | 6.8 | 6.08 | 10.74 | 10.76 |
|  | *Apterodon macrognathus* | 20.28 | 24.48 | 14.5 | 20.23 | 13.18 | 23.77 | 29.4 |
|  | *Apterodon macrognathus* | 16.19 | 16.01 | 10.12 | 19.38 | 11.52 | 22.17 | 24.89 |
|  | *Pterodon africanus* | 22.23 | 31.46 | 18.6 | 27.37 | 17.58 | 26.86 | 30.1 |
|  | DPC 10831 | 3.91 | 3.8 | 2.3 | 5.84 | 2.83 | 4.99 | 6.91 |
|  | DPC 11670 | 3.95 | 4.93 | 3.5 | 5.47 | 3.13 | 6.11 | 6.06 |
|  | DPC 15436 | 4.61 | 5.47 | 3.43 | 5.55 | 2.81 | 5.51 | 6.54 |
| **Fig Code** | **Name** | **MinDT** | **MEW** | **LEW** | **LEL** | **MEL** | **PTW** | **MEA** |
| 1 | *Ailurus fulgens* | 6.38 | 6.67 | 7.62 | 3.36 | 7.26 | 7.43 | 156.63 |
| 2 | *Arctictis binturong* | 7.07 | 6.88 | 9.76 | 4.38 | 6.56 | 10.12 | 159.69 |
| 3 | *Arctogalidia trivirgata* | 4.54 | 4.26 | 6.65 | 2.91 | 5.34 | 6.7 | 160.68 |
| 4 | *Atilax paludinosus* | 5.04 | 5.17 | 7.99 | 2.67 | 4.4 | 6.1 | 161.14 |
| 5 | *Bassaricyon gabbii* | 3.76 | 4.03 | 4.46 | 2.03 | 3.4 | 4.11 | 155.29 |
| 6 | *Bassariscus astutus* | 3.81 | 3.24 | 6.52 | 1.59 | 3.1 | 4.68 | 158.21 |
| 7 | *Canis latrans* | 12.8 | 13.06 | 13.22 | 3.86 | 7.6 | 11.54 | 160.81 |
| 8 | *Canis lupus* | 17.48 | 14.7 | 16.45 | 5.49 | 9.99 | 12.92 | 161.62 |
| 9 | *Civettictis civetta* | 8.57 | 12.21 | 10.76 | 2.27 | 6.36 | 9.24 | 153.01 |
| 10 | *Crocuta crocuta* | 17.88 | 28.31 | 21.48 | 3.89 | 7.64 | 17.95 | 146.97 |
| 11 | *Cryptoprocta ferox* | 7.06 | 6.48 | 7.27 | 1.98 | 7.23 | 9.19 | 145.76 |
| 12 | *Cynictis penicillata* | 3.11 | 3.88 | 3.98 | 1.6 | 3.24 | 4.24 | 148.37 |
| 13 | *Eira barbara* | 8.07 | 7.61 | 13.76 | 2.81 | 7.86 | 10.6 | 136.77 |
| 14 | *Eupleres goudotii* | 4.95 | 5.05 | 5.94 | 1.81 | 5.41 | 4.94 | 143.53 |
| 15 | *Felis pardalis* | 9.13 | 9.56 | 12.66 | 4.94 | 7.36 | 10.73 | 145.34 |
| 16 | *Felis wiedii* | 5.74 | 6.14 | 7.1 | 2.48 | 5.98 | 6.36 | 151.37 |
| 17 | *Fossa fossana* | 4.55 | 4.74 | 4.94 | 1.96 | 3.74 | 4.42 | 137 |
| 18 | *Galerella sanguineus* | 2.82 | 3.32 | 4.22 | 1.54 | 2.78 | 3.42 | 147.41 |
| 19 | *Genetta genetta* | 4.06 | 4.42 | 5 | 1.58 | 4.2 | 5.05 | 139 |
| 20 | *Genetta maculata* | 3.69 | 4.4 | 5.25 | 1.92 | 4.39 | 4.98 | 140.96 |
| 21 | *Gulo gulo* | 10.65 | 10.7 | 16.96 | 5.3 | 10.17 | 12.11 | 126.4 |
| 22 | *Herpestes ichneumon* | 5.48 | 6 | 8.52 | 3.16 | 4.08 | 5.91 | 140.28 |
| 23 | *Hyaena brunnea* | 15.39 | 14 | 12.64 | 4.74 | 2.08 | 17.76 | 115 |
| 24 | *Hyaena hyaena* | 15.35 | 11.34 | 15.03 | 7.67 | 2.8 | 13.4 | 143.5 |
| 25 | *Lontra canadensis* | 6.97 | 6.51 | 9.2 | 5.4 | 8.03 | 8.97 | 147.87 |
| 26 | *Lontra longicaudus* | 7.21 | 5.62 | 8.85 | 4.11 | 8.86 | 7.65 | 145.55 |
| 27 | *Lycaon pictus* | 14.78 | 7.99 | 9.13 | 1.87 | 2.04 | 10.98 | 160.48 |
| 28 | *Lynx rufus* | 8.4 | 8.61 | 7.14 | 1.84 | 7.36 | 9.68 | 130.83 |
| 29 | *Martes americana* | 4 | 3.39 | 3.15 | 0.69 | 3.51 | 3.95 | 140.7 |
| 30 | *Martes pennanti* | 5 | 4.81 | 4.78 | 0.84 | 6.44 | 6.18 | 142.9 |
| 31 | *Meles meles* | 9 | 9.19 | 6.83 | 2.46 | 9.21 | 10.45 | 142.9 |
| 32 | *Melogale moschata* | 3.63 | 3.77 | 3.29 | 1.05 | 4.96 | 4.85 | 142.79 |
| 33 | *Mephitis mephitis* | 4.57 | 3.74 | 5 | 2.14 | 5.06 | 6.23 | 154.28 |
| 34 | *Mungos mungo* | 3.42 | 3.51 | 3.02 | 0.75 | 3.88 | 4.49 | 161.99 |
| 35 | *Nandinia binotata* | 4.4 | 4.98 | 5.1 | 1.37 | 5.96 | 6.21 | 157.63 |
| 36 | *Nasua nasua* | 5.36 | 6.13 | 6.26 | 1.64 | 9.72 | 6.7 | 151.43 |
| 37 | *Neovison vison* | 3.24 | 3.02 | 3.33 | 0.79 | 3.72 | 4.08 | 147.76 |
| 38 | *Paguma larvata* | 6.04 | 5.64 | 5.31 | 1.25 | 5 | 7.31 | 147.5 |
| 39 | *Panthera leo* | 22.26 | 17.22 | 18.47 | 3.87 | 7.18 | 29.68 | 119.09 |
| 40 | *Paradoxurus hermaphroditus* | 5.39 | 5.71 | 5.03 | 1.62 | 6.76 | 6.55 | 153.65 |
| 41 | *Poiana richardsoni* | 2.56 | 3.22 | 2.17 | 0.66 | 2.79 | 3.58 | 152.51 |
| 42 | *Potos flavus* | 5.01 | 4.52 | 4.21 | 1.34 | 6.3 | 7.57 | 164.11 |
| 43 | *Prionodon linsang* | 3.89 | 4.14 | 3.35 | 0.92 | 4.11 | 4.75 | 136.47 |
| 44 | *Procyon lotor* | 7.36 | 5.98 | 5.02 | 1.32 | 7.54 | 8.1 | 156.35 |
| 45 | *Proteles cristatus* | 9.37 | 6.47 | 6 | 1.58 | 1.15 | 8.71 | 150.53 |
| 46 | *Pteronura brasiliensis* | 9.79 | 8.7 | 5.95 | 1.77 | 11.64 | 13.1 | 161.5 |
| 47 | *Suricata suricatta 3* | 3.51 | 3.67 | 2.92 | 0.89 | 3.3 | 3.92 | 163.08 |
| 48 | *Urocyon cinereoargenteus* | 7.1 | 4.9 | 4.16 | 1.51 | 1.66 | 6.5 | 169.72 |
| 49 | *Ursus americanus* | 18.38 | 24.02 | 15.37 | 6 | 21.6 | 27.31 | 135.45 |
| 50 | *Ursus arctos* | 20.45 | 28.63 | 16.38 | 4 | 23.6 | 32.21 | 117.44 |
| 51 | *Viverra tangalunga* | 5.89 | 5.87 | 4.52 | 1.8 | 4.2 | 6.81 | 142.34 |
| 52 | *Viverra zibetha* | 7.3 | 8.64 | 5.63 | 1.66 | 6.9 | 8.59 | 130.25 |
| 53 | *Viverricula indica* | 5.01 | 4.24 | 2.9 | 0.67 | 3.65 | 4.94 | 123.81 |
| 54 | *Vulpes macrotis* | 5.6 | 2.53 | 2.62 | 0.67 | 0.78 | 4.87 | 158.89 |
| 55 | *Vulpes vulpes* | 7.68 | 3.15 | 3.21 | 1.13 | 1.48 | 6.29 | 167.23 |
|  | *Apterodon langebadreae* | 10.83 | 13.06 | 10.48 | 4.11 | 16.26 | 16.18 | 165.75 |
|  | *Hyaenodon horridus* | 15.8 | 15.26 | 9.33 | 2.52 | 11.59 | 16.62 | 145.26 |
|  | *Limnocyon verus* | 6.35 | 5.25 | 5.36 | 2.3 | 8.22 | 8.54 | 167.31 |
|  | *Sinopa* sp. | 6.75 | 5.82 | 3.24 | 0.93 | 7.13 | 7.14 | 165.71 |
|  | *Tritemnodon hians* | 8.28 | 6.33 | 4.8 | 1.56 | 8.57 | 11.19 | 164.13 |
|  | *Arfia shoshoniensis* | 7.62 | 6.95 | 3.62 | 0.73 | 1.9 | 8.24 | 141.12 |
|  | *Apterodon macrognathus* | 16.21 | 15.02 | 8.69 | 2.92 | 20.11 | 26.18 | 161.19 |
|  | *Apterodon macrognathus* | 12.54 | 14.93 | 8.84 | 3.42 | 18.62 | 21.99 | 159.02 |
|  | *Pterodon africanus* | 20.2 | 24.2 | 13.04 | 3.74 | 7.87 | 23.11 | 162.8 |
|  | DPC 10831 | 4.22 | 3.29 | 2.24 | 2.38 | 3.78 | 6.94 | 159.23 |
|  | DPC 11670 | 5.36 | 3.23 | 2.5 | 1.64 | 2.81 | 6.42 | 163.61 |
|  | DPC 15436 | 4.62 | 3.51 | 2.63 | 0.95 | 3.96 | 5.23 | 161.48 |

Measurement abbreviations from Fig 2. Locomotor classification for each species is presented in Table 5. Fig Code corresponds with SM-Fig 1.


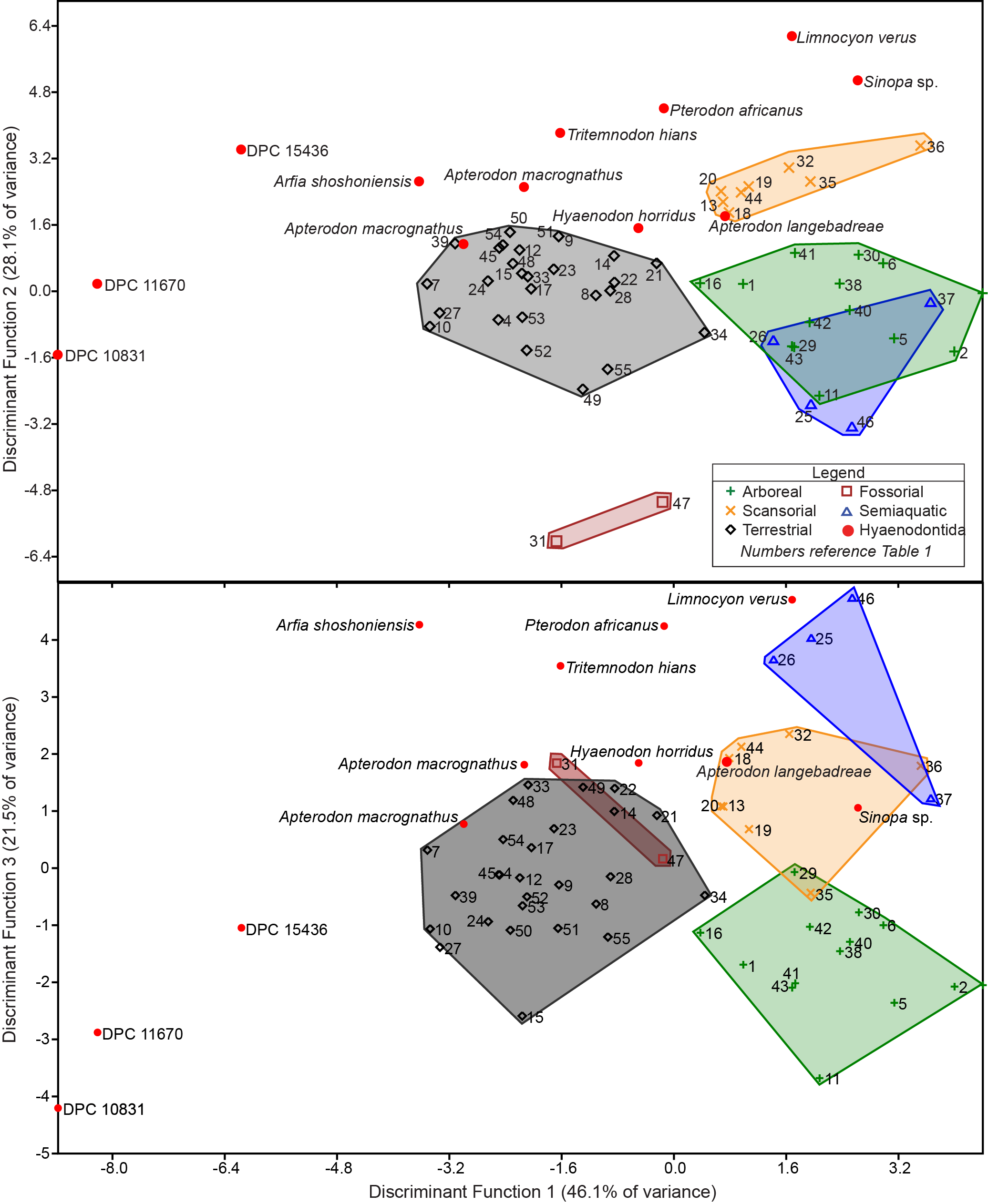


**SM-Fig 1. Locomotor categorization of hyaenodontidan humeri on DF1, DF2, and DF3 based on species means.** The results of discriminant function analysis for DF1 (explains 46.1% of variance) plotted against DF2 (explains 28.1% of variance) and DF3 (explains 21.5% of variance). The analysis is based on 21 measurements (see Fig 2) collected for 55 carnivoran species and 12 hyaenodont specimens. Numbers correspond to specimen numbers in SM-Table 1. **Green plus**, Arboreal carnivoran species; **Yellow X**, Scansorial carnivoran species; **Black diamond**, Terrestrial carnivoran species; **Red squares**, Fossorial carnivoran species; **Blue triangles**, Semiaquatic carnivoran species; **Red dots**, hyaenodonts with unassigned locomotor behaviors.

**SM-Table 2. Discriminate Function scores and predicted locomotor groups**

| Fig Code | Names | Assigned group | Predicted group | Prob. | 2nd group | 2nd prob. | DF1 | DF2 | DF3 | DF4 |
| --- | --- | --- | --- | --- | --- | --- | --- | --- | --- | --- |
| 1 | *Ailurus fulgens* | A | A | 98.6 | T | 1.4 | 0.988 | 0.179 | -1.691 | 1.064 |
| 2 | *Arctictis binturong* | A | A | 100.0 | Se | 0.0 | 3.998 | -1.450 | -2.075 | 0.403 |
| 3 | *Arctogalidia trivirgata* | A | A | 100.0 | S | 0.0 | 4.401 | -0.046 | -2.050 | 1.748 |
| 4 | *Atilax paludinosus* | T | T | 100.0 | A | 0.0 | -2.498 | -0.684 | -0.110 | -0.691 |
| 5 | *Bassaricyon gabbii* | A | A | 100.0 | T | 0.0 | 3.140 | -1.134 | -2.358 | 1.124 |
| 6 | *Bassariscus astutus* | A | A | 99.6 | S | 0.4 | 2.984 | 0.676 | -0.999 | 0.562 |
| 7 | *Canis latrans* | T | T | 100.0 | S | 0.0 | -3.513 | 0.184 | 0.318 | 1.076 |
| 8 | *Canis lupus* | T | T | 99.8 | A | 0.2 | -1.109 | -0.095 | -0.626 | 1.668 |
| 9 | *Civettictis civetta* | T | T | 99.7 | S | 0.3 | -1.639 | 1.326 | -0.290 | -0.498 |
| 10 | *Crocuta crocuta* | T | T | 100.0 | F | 0.0 | -3.474 | -0.846 | -1.063 | -1.295 |
| 11 | *Cryptoprocta ferox* | A | A | 100.0 | T | 0.0 | 2.075 | -2.517 | -3.679 | -0.132 |
| 12 | *Cynictis penicillata* | T | T | 100.0 | S | 0.0 | -2.196 | 1.000 | -0.167 | 1.398 |
| 13 | *Eira barbara* | S | S | 99.8 | T | 0.1 | 0.704 | 2.156 | 1.080 | -1.105 |
| 14 | *Eupleres goudotii* | T | T | 90.9 | S | 9.1 | -0.850 | 0.857 | 0.997 | -0.635 |
| 15 | *Felis pardalis* | T | T | 100.0 | A | 0.0 | -2.162 | 0.433 | -2.589 | -0.980 |
| 16 | *Felis wiedii* | A | A | 71.5 | T | 26.0 | 0.377 | 0.196 | -1.130 | -1.251 |
| 17 | *Fossa fossana* | T | T | 100.0 | S | 0.0 | -2.031 | 0.065 | 0.363 | 0.299 |
| 18 | *Galerella sanguineus* | S | S | 99.9 | T | 0.1 | 0.788 | 1.900 | 1.929 | -0.723 |
| 19 | *Genetta genetta* | S | S | 99.9 | T | 0.0 | 1.069 | 2.528 | 0.684 | -0.680 |
| 20 | *Genetta maculata* | S | S | 99.7 | T | 0.3 | 0.674 | 2.413 | 1.086 | 0.085 |
| 21 | *Gulo gulo* | T | T | 94.4 | S | 5.0 | -0.243 | 0.678 | 0.926 | 1.607 |
| 22 | *Herpestes ichneumon* | T | T | 97.4 | S | 2.5 | -0.844 | 0.216 | 1.400 | -0.198 |
| 23 | *Hyaena brunnea* | T | T | 99.9 | S | 0.1 | -1.709 | 0.535 | 0.695 | 0.627 |
| 24 | *Hyaena hyaena* | T | T | 100.0 | A | 0.0 | -2.646 | 0.250 | -0.935 | 1.124 |
| 25 | *Lontra canadensis* | Se | Se | 100.0 | A | 0.0 | 1.956 | -2.715 | 4.039 | 2.601 |
| 26 | *Lontra longicaudus* | Se | Se | 100.0 | S | 0.0 | 1.420 | -1.174 | 3.661 | 0.306 |
| 27 | *Lycaon pictus* | T | T | 100.0 | A | 0.0 | -3.334 | -0.518 | -1.383 | 0.148 |
| 28 | *Lynx rufus* | T | T | 99.6 | A | 0.3 | -0.904 | 0.016 | -0.147 | -0.323 |
| 29 | *Martes americana* | A | A | 99.3 | Se | 0.3 | 1.718 | -1.346 | -0.068 | -0.518 |
| 30 | *Martes pennanti* | A | A | 95.0 | S | 5.0 | 2.635 | 0.881 | -0.774 | -0.519 |
| 31 | *Meles meles* | F | F | 100.0 | Se | 0.0 | -1.670 | -6.027 | 1.842 | -1.415 |
| 32 | *Melogale moschata* | S | S | 100.0 | Se | 0.0 | 1.643 | 2.982 | 2.352 | -0.193 |
| 33 | *Mephitis mephitis* | T | T | 99.8 | S | 0.2 | -2.078 | 0.357 | 1.461 | -1.439 |
| 34 | *Mungos mungo* | T | A** | 73.2 | T | 26.0 | 0.439 | -0.994 | -0.474 | -1.860 |
| 35 | *Nandinia binotata* | S | S | 98.4 | A | 1.6 | 1.948 | 2.647 | -0.431 | -0.715 |
| 36 | *Nasua nasua* | S | S | 100.0 | A | 0.0 | 3.517 | 3.512 | 1.794 | -0.597 |
| 37 | *Neovison vison* | Se | A** | 53.3 | Se | 35.7 | 3.660 | -0.252 | 1.230 | -0.276 |
| 38 | *Paguma larvata* | A | A | 99.9 | S | 0.1 | 2.365 | 0.186 | -1.454 | -0.752 |
| 39 | *Panthera leo* | T | T | 100.0 | S | 0.0 | -3.114 | 1.154 | -0.476 | 1.292 |
| 40 | *Paradoxurus hermaphroditus* | A | A | 100.0 | S | 0.0 | 2.507 | -0.453 | -1.290 | -1.256 |
| 41 | *Poiana richardsoni* | A | A | 99.7 | S | 0.2 | 1.728 | 0.924 | -2.016 | 0.091 |
| 42 | *Potos flavus* | A | A | 99.9 | T | 0.0 | 1.936 | -0.747 | -1.027 | -0.098 |
| 43 | *Prionodon linsang* | A | A | 100.0 | T | 0.0 | 1.685 | -1.327 | -2.094 | 0.850 |
| 44 | *Procyon lotor* | S | S | 100.0 | T | 0.0 | 0.960 | 2.386 | 2.128 | -2.251 |
| 45 | *Proteles cristatus* | T | T | 100.0 | S | 0.0 | -2.484 | 1.047 | -0.115 | 1.185 |
| 46 | *Pteronura brasiliensis* | Se | Se | 100.0 | F | 0.0 | 2.540 | -3.256 | 4.742 | 1.844 |
| 47 | *Suricata suricatta* | F | F | 100.0 | T | 0.0 | -0.159 | -5.080 | 0.164 | -2.906 |
| 48 | *Urocyon cinereoargenteus* | T | T | 99.9 | S | 0.1 | -2.291 | 0.671 | 1.191 | -0.393 |
| 49 | *Ursus americanus* | T | T | 73.0 | F | 26.8 | -1.295 | -2.360 | 1.421 | -0.872 |
| 50 | *Ursus arctos* | T | T | 100.0 | S | 0.0 | -2.332 | 1.427 | -1.085 | 0.361 |
| 51 | *Viverra tangalunga* | T | T | 99.6 | S | 0.4 | -1.652 | 2.035 | -1.051 | -0.230 |
| 52 | *Viverra zibetha* | T | T | 100.0 | A | 0.0 | -2.094 | -1.420 | -0.499 | 1.036 |
| 53 | *Viverricula indica* | T | T | 100.0 | A | 0.0 | -2.157 | -0.621 | -0.656 | -0.751 |
| 54 | *Vulpes macrotis* | T | T | 100.0 | S | 0.0 | -2.434 | 1.120 | 0.505 | 1.567 |
| 55 | *Vulpes vulpes* | T | T | 97.0 | A | 3.0 | -0.941 | -1.876 | -1.205 | 1.485 |
| 56 | *Apterodon macrognathus* | ? | T | 98.9 | S | 1.1 | -2.133 | 2.515 | 1.813 | 2.661 |
| 57 | *Apterodon macrognathus* | ? | T | 100.0 | S | 0.0 | -2.995 | 1.139 | 0.773 | 2.083 |
| 58 | *Arfia shoshoniensis* | ? | T | 100.0 | S | 0.0 | -3.628 | 2.650 | 4.266 | 5.356 |
| 59 | *Hyaenodon horridus* | ? | S | 62.5 | T | 37.5 | -0.503 | 1.524 | 1.844 | 0.624 |
| 60 | DPC 10831 | ? | T | 100.0 | F | 0.0 | -8.776 | -1.530 | -4.203 | -0.383 |
| 61 | DPC 11670 | ? | T | 100.0 | F | 0.0 | -8.213 | 0.181 | -2.878 | -0.018 |
| 62 | DPC 15436 | ? | T | 100.0 | S | 0.0 | -6.163 | 3.419 | -1.045 | 0.466 |
| 63 | *Limnocyon verus* | ? | S | 100.0 | Se | 0.0 | 1.684 | 6.153 | 4.704 | -1.012 |
| 64 | *Pterodon africanus* | ? | S | 100.0 | T | 0.0 | -0.140 | 4.412 | 4.242 | -0.816 |
| 65 | *Sinopa* sp. | ? | S | 100.0 | A | 0.0 | 2.622 | 5.087 | 1.058 | -1.968 |
| 66 | *Tritemnodon hians* | ? | S | 99.9 | T | 0.1 | -1.617 | 3.819 | 3.545 | -1.924 |
| 72 | *Apterodon langebadrae* | ? | S | 100.0 | T | 0.0 | 0.75 | 1.808 | 1.847 | -2.165 |

**A**, Arboreal; **F**, Fossorial; **S**, Scansorial; **Se**, Semiaquatic; **T**, Terrestrial; **prob.**, Probability (%); ****,** Indicates a misclassified species

**SM-Table 3. Pooled within-group correlations with species means**

| Measurement | DF1 (46.1%) | DF2 (28.1%) | DF3 (21.5%) | DF4 (4.3%) |
| --- | --- | --- | --- | --- |
| TDW | -.444^*^ | -0.016 | -0.105 | 0.233 |
| MEL | .339^*^ | -0.041 | 0.189 | -0.327 |
| MaxAT | -.308^*^ | 0.157 | -0.235 | 0.169 |
| MinDT | -.290^*^ | -0.159 | 0.003 | 0.184 |
| MinAT | -.252^*^ | 0.052 | -0.124 | 0.199 |
| OFH | -.202^*^ | -0.007 | 0.044 | -0.109 |
| MEA | .109^*^ | -0.084 | -0.034 | -0.045 |
| CW | .071^*^ | 0.003 | -0.059 | -0.059 |
| MedPT | -0.086 | .241^*^ | 0.100 | 0.130 |
| OFW | 0.115 | -.230^*^ | 0.043 | -0.059 |
| PTW | 0.076 | -.113^*^ | 0.047 | -0.037 |
| LEW | 0.038 | .086^*^ | 0.015 | 0.021 |
| AW | 0.104 | 0.056 | -.390^*^ | 0.142 |
| TW | 0.030 | 0.053 | -.378^*^ | 0.142 |
| SCW | 0.089 | -0.107 | .274^*^ | -0.092 |
| MaxC | -0.221 | -0.057 | -0.096 | .427^*^ |
| CDW | -0.138 | -0.041 | 0.088 | .313^*^ |
| MEW | -0.119 | -0.040 | -0.030 | -.249^*^ |
| LEL | 0.050 | -0.023 | 0.081 | .221^*^ |
| LatPT | 0.116 | 0.088 | -0.082 | .147^*^ |
| MinPT | 0.044 | 0.054 | -0.011 | .140^*^ |

Pooled within-group correlations between humeral measurements and discriminant functions. *****, largest correlation with discriminant function.

**SM-Table 4. Species Mean Confusion Matrix**

| Observed group (Total) | Arboreal | Fossorial | Scansorial | Semiaquatic | Terrestrial | % Correct |
| --- | --- | --- | --- | --- | --- | --- |
| Arboreal (14) | 14 (10) | 0 (0) | 0 (2) | 0 (0) | 0 (2) | 100 (71.4) |
| Fossorial (2) | 0 (0) | 2 (2) | 0 (0) | 0 (0) | 0 (0) | 100 (100) |
| Scansorial (8) | 0 (1) | 0 (0) | 8 (6) | 0 (0) | 0 (1) | 100 (75) |
| Semiaquatic (4) | 1 (1) | 0 (1) | 0 (0) | 3 (2) | 0 (0) | 75 (50) |
| Terrestrial (27) | 1 (2) | 0 (2) | 0 (3) | 0 (1) | 26 (19) | 96.2 (70.4) |
|  |  |  |  |  | Total | 96.4 (70.9) |

Confusion matrix with species sorted into correct group (taxa sorted into correct group using leave-one-out cross-reference analysis). Parentheses indicate number of species sorted into group in leave-one-out cross-validation analysis.
